# A Multilevel Meta-Analysis of Single-Case Research on Interventions for Externalizing Behavior Problems in Children and Adolescents

**DOI:** 10.1016/j.jaacop.2025.12.002

**Published:** 2025-12-18

**Authors:** Shawn I. Kok, Laura M. Fetz, Yvonne A.J. Stikkelbroek, Patty Leijten, Janneke Staaks, Marija Maric

**Affiliations:** a University of Amsterdam, Amsterdam, the Netherlands; b Utrecht University, Utrecht, the Netherlands

**Keywords:** externalizing problem behaviors, children and adolescents, treatment effectiveness, single-case designs, meta-analysis

## Abstract

**Objective:**

The overall effectiveness of interventions for youth externalizing behavior problems was studied using a review and a meta-analysis of published single-case research in children and adolescents.

**Method:**

Scientific databases and gray literature were searched for quantitative single-case studies concerned with the treatment of externalizing behavior problems in children and adolescents. Study and case characteristics were extracted, and the studies were rated for quality. Raw graph data from individual cases were aggregated and analyzed by means of multilevel meta-analysis for single-case research.

**Results:**

We identified 78 studies including 270 cases (mean age = 8.70 years; 71.48% male individuals). Overall, positive within-person changes during the treatment as opposed to baseline were observed. Reductions in symptoms did not carry through the follow-up phase. However, variations in treatment effects were observed, with larger variations among studies than among cases. Furthermore, studies using observational assessment methods yielded stronger results than studies using questionnaires to assess outcomes. Although the scores for the external validity of the studies were above average, the scores for internal validity were below average.

**Conclusion:**

Although part of the internal validity result can be attributed to underreporting certain quality standards in the studies, it is of great importance for the field of single-case research to start implementing existing methodological guidelines and to comprehensively report case-relevant information. This will, in addition, facilitate our understanding of the variability in treatment outcomes for specific children, and will enable us to learn more about the effects of interventions in diverse youth populations.

**Study registration information:**

Preregistration Meta-analysis of Single-Case Research on Interventions for Externalizing Behavioral Problems in Children and Adolescents; https://doi.org/10.17605/osf.io/4bewa

## Plain language summary

Single-case studies of youth with externalizing behaviors allow consideration of treatment effects within individuals over time, but meta-analyses of this research have been limited. This study aggregated and summarized the results from 78 published studies of 270 single-case research in the area of youth externalizing problems. Overall, positive changes in externalizing problems were found, and the studies that used observational methods to depict externalizing behaviors reported more changes than studies that used questionnaire methods. Results suggest the quality of single-case studies can be improved.

Externalizing behavior problems in youth cover a wide range of oppositional and deviant behaviors, such as aggression, defiance, and rule-breaking behavior.^1^ The prevalence of these behaviors is concerning, given their significant interference with daily functioning and their profound consequences. In the short term, children with externalizing problems often struggle academically, face peer rejection, and experience family conflict. Over the long term, if left untreated, these behaviors can escalate into more severe issues, including criminal behavior, substance abuse, and significant mental health challenges in adulthood.^2^^,^^3^ Although children may exhibit externalizing behaviors within environments such as home and school, these same settings can foster positive change by offering structure and support that enable effective interventions.^4^^,^^5^ Tailoring interventions to make use of the strengths of these settings, or addressing specific environmental stressors, can significantly improve treatment outcomes and prevent further escalation of externalizing behaviors. In this sense, the immediate environment often acts as a crucial moderator of intervention success.

A broad range of such interventions have been developed over the past decades.^3^^,^^6^ The repertoire now comprises multiple interventions targeting caregivers, school environments, and children directly. For instance, parent management training programs teach caregivers effective strategies to reduce disruptive behavior, such as consistent discipline, setting clear expectations, and using positive reinforcement to encourage desired behavior.^7^ In school settings, interventions such as the Good Behavior Game aim to improve classroom conduct by reinforcing prosocial behaviors while decreasing disruptive acts.^8^ At an individual level, children benefit from cognitive–behavioral therapy (CBT) and social skills training focusing on helping them to develop self-control, problem-solving abilities, and emotional regulation, by addressing behaviors such as aggression, defiance, and impulsivity.^9^ These programs have been shown to be effective in reducing externalizing behaviors and lowering the risk of developing serious emotional and antisocial problems later in life when they are implemented in environments, and the techniques—such as positive reinforcement, consistent discipline, and clear communication—are consistently practiced by caregivers, teachers, therapists, and youths themselves.^10^

At the same time, most evidence supporting these programs is based on group studies (ie, randomized controlled trials [RCTs]) that focus primarily on overall treatment effects across populations.^11^^,^^12^ Although these studies provide valuable insights, they often overlook how treatment effects vary within individuals over time.^13^^,^^14^ This limitation makes it difficult to account for the nuanced, individual treatment responses that are critical in personalizing interventions. Children with externalizing behavior problems may share some common traits, but there are substantial individual differences in how these behaviors manifest and in the contextual factors that influence them.^15^

Quantitative single-case studies have long been seen as a valuable tool in addressing this absence of within-subject comparisons in RCTs and reviews thereof.^13^ Interventions for externalizing behavior problems, in particular, have been thoroughly studied using single-case studies for decades, following renewed interest from researchers and clinicians.^16^^,^^17^ For instance, a recent theoretical review of single-case group contingency on targeting prosocial and antisocial behavior included 22 studies.^18^ Using single-case studies rather than RCTs has the advantage that this allows for a thorough inspection of case-level characteristics. This is especially relevant given the large heterogeneity across participants: although some interventions may work well for certain individuals, the same interventions may yield different results for others. However, despite the rising popularity of single-case studies, there have only been a handful of meta-analyses examining the findings from single-case studies on externalizing problems in youth, with most of them being limited to only a single type of intervention.^19^, ^20^, ^21^, ^22^ Although narrowing the scope in this way can be valuable given the high heterogeneity of samples, it overlooks the fact that most interventions for externalizing problems share a behavioral foundation. As a result, analyzing data from studies using a broad range of interventions makes sense and provides a unique opportunity to investigate critical moderators such as the context in which the intervention occurs. In addition, these previous meta-analyses have not harnessed single-case methodology to its fullest, investigating only the effect size based on differences in means between phases (eg, computing the Hedges' *g* for the difference between the treatment phase average and the baseline average scores). Single-case studies offer a methodological advantage by enabling the application of multilevel modeling techniques.^23^ This approach allows for a more direct analysis of data on the level of an individual client, providing a more nuanced understanding of how interventions work across different contexts, rather than relying solely on aggregated effect sizes typically used in traditional meta-analyses. This ability to capture both overall patterns and individual variations leads to more robust and comprehensive findings that can inform more personalized treatment approaches.

In light of the above, this study presents the first meta-analysis of single-case studies that includes a broad scope of intervention programs and treatment moderators and that analyzes data with multilevel meta-analysis for single-case research. Our primary aims are as follows: (1) to provide a comprehensive overview of published single case research in the field of single-case studies on externalizing behavior in children and adolescents and the quality of these studies; (2) to examine overall efficacy of psychosocial interventions in this area; (3) to explore variations in effects over time among different interventions; and (4) to examine how different intervention settings and participants characteristics moderate these effects. We emphasize that this study is exploratory in nature, and that we did not specify concrete hypotheses beyond a general reduction in symptoms following treatment.

## Method

This study was pre-registered on the Open Science Framework (OSF) in April 2024 at https://doi.org/10.17605/osf.io/4bewa. However, because of the unknown nature of potential studies, we finalized our analysis plan during the data extraction phase. The meta-analysis was conducted in accordance with the Preferred Reporting Items for Systematic Reviews and Meta-Analyses (PRISMA) guidelines (Supplement 1, available online).^24^

### Literature Search

**Figure 1:**
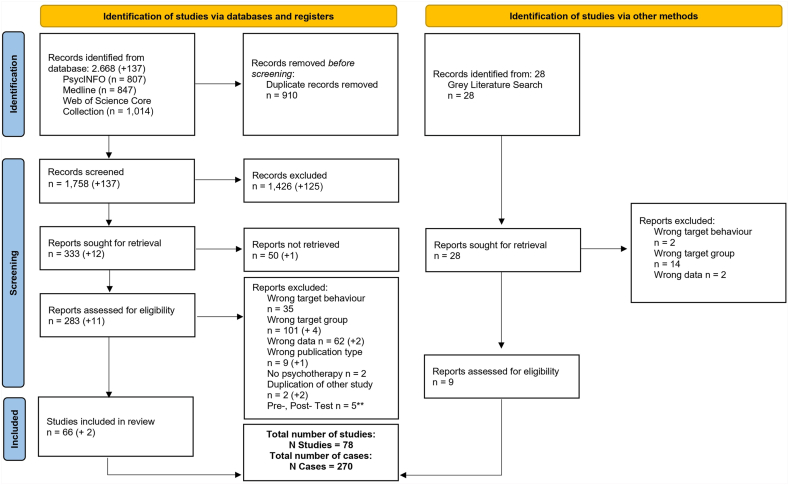
PRISMA Flow Diagram of Included Studies and Cases ***Note:****The numbers in parentheses reflect the updated search results conducted in November 2024. This figure is based on the Preferred Reporting Items for Systematic Reviews and Meta-Analyses (PRISMA) guidelines in Page* et al.*^24^**∗∗Studies that reported only pre- and post-test data were initially included. However, because of the limited number of such studies, they were excluded from the analysis for this article.*

We developed a search strategy targeting studies within 4 major categories: (1) single-case (experimental) designs (SC[E]Ds), (2) children and adolescents, (3) externalizing behavior, and (4) interventions. A detailed list of search terms used is provided in Supplement 2, available online. We conducted the initial search across PsycINFO, Medline, and Web of Science in January 2024, yielding a total of 2,668 records. To ensure the inclusion of the most recent literature, we repeated the search in September 2024, resulting in an additional 137 records. To further enhance our review, we conducted a manual screening of the reference lists from included studies and searched preprint platforms such as PsyArXiv and OSF for other relevant studies. In addition, we reached out to corresponding authors to obtain access to manuscripts not publicly available. The details of our literature search are presented in Figure 1.

### Study Selection

Abstracts and full-texts were independently screened by 2 raters, with 20% of the abstracts subjected to double screening to ensure reliability. Disagreements were resolved during discussions between the raters, and in case the raters could not reach agreement, the study was discussed during meetings with the senior team members. Studies were included if they used quantitative SCED methods and focused on children and adolescents 2 to 17 years of age with externalizing behavior problems determined during an initial screening. We targeted intervention studies that used psychosocial interventions for curative purposes, and that reported symptom severity for individual children multiple times across baseline and intervention phases. Exclusion criteria were as follows: cases involving participants with intellectual disabilities (IQ <70) and/or medical conditions (eg, traumatic brain injury) that could interfere with externalizing problems conditions or treatment of it. Initially, we considered studies with only pre- and post-test assessments; however, a lack of sufficient data in this subset led to its exclusion.

### Outcome Variables and Moderators

Outcome variables consisted of repeated assessments, in the form of graph data (Data Extraction section). In studies with multiple outcome variables, the most relevant ones to the primary problem behavior of the individual participant were selected. In most studies, the main outcome variable was assessed using observations (73%), compared to questionnaires (27%). Previous research has shown that certain moderators may have an impact on the effectiveness of youth interventions.^25^ Therefore, we tested for the potential influence of the following moderators: type of externalizing problem (aggressive behavior, antisocial behavior, noncompliance behavior, oppositional and stubborn behavior, quick-tempered and irritable behavior, and other problems), treatment context (clinic, home, school, and both home and school), and type of assessment (observation, or report). To classify the type of externalizing behavior, we used the guidelines provided by the Netherlands Youth Institute based on previous empirical research.^26^^,^^27^ The subcategory other problems included off-task behavior, social difficulties, negative vocalizations (sometimes with self-injury), disruptive responses in structured settings, and general behavioral issues.

### Data Extraction

Study characteristics were extracted manually in Microsoft Excel. Graph data were extracted using Digitzelt.^28^^,^^29^ Three independent raters performed extraction of graph data and characteristics. Prior to analysis, all extracted data and study characteristics were cross-checked.

### Quality Rating of the Studies

Quality was assessed using 15-item Risk of Bias in N-of-1 Trials (RoBiNT) scale, which contains internal validity (IV; 7 items, eg, “design”), external validity and interpretation (EVI; 8 items, eg respectively, “therapeutic setting”, “data analyses”).^30^ Items are rated on a 3-point scale (0-2), with a maximum total score of 30, and subscale maximums of 14 for IV and 16 for EVI. Author YS and MM rated independently 20% of the studies resulting in interrater reliability (intracorrelation coefficient [ICC]) of 0.73. Differences were resolved through discussions.

### Statistical Analyses

Unlike traditional meta-analyses, we did not compute summary effect sizes for each study. Instead, we pooled the raw data from all cases and analyzed these using multilevel mixed-effects models in R 4.2.2 for Windows.^23^^,^^31^ In particular, we applied the functionality provided with the *lme4* package.^32^

#### Data Preparation

Data preparation consisted of a multi-tiered process in which data from each study were harmonized and prepared for analysis. First, time was centered around the transition between the baseline and treatment phase, so that treatment for each case began at time = zero. Negative values thereby represent values from the baseline phase. Second, we rescaled time for each study to a daily scale to minimize bias regarding the number of sessions. This meant that studies with weekly measurements, for instance, were assigned intervals of 7 (eg, 0, 7, 14). Follow-up data (eg, 6-month follow-up) were also rescaled accordingly. Finally, we recoded the phase variable into a dummy code indicating whether treatment was in progress at a given time point (0 = no treatment, 1 = treatment). In this way, all observations prior to the treatment onset were coded as 0, and all observations during the treatment phase were coded as 1.

#### Standardization of Outcome Variables

As the outcome variables across studies were reported in different formats (eg, standard scores, percentages, frequencies, and ratios), we standardized the data before analysis using the method proposed by Van Den Noortgate and Onghena.^23^ This standardization method involves 2 steps. First, for each study we fitted a 2-level model predicting the outcome variable using phase (baseline phase, treatment phase, and if available follow-up phase) and time, with random effects for all predictors across each case. Second, the resulting scores from each case were divided by the residual standard deviation of the respective model. For studies reporting only a single case, we applied an adapted approach, fitting a linear ordinary regression model and dividing scores by the estimated residual standard deviation.

#### Analysis of Average Effects and Moderators

Using the standardized data, we conducted a 3-level mixed-effects linear regression model to meta-analyze the outcomes across cases and studies. The outcome variable (ie, the severity of externalizing problems) was explained by phase, time, and their interaction. In this model, the intercept and the effect of time represent the expected outcome level at the start of the baseline phase and the time trend during baseline, respectively. The phase effects indicate the immediate treatment and follow-up effects, and the interactions between time and the phase terms indicate the change in trend throughout the phase over time. Random effects were included at both the case and study levels. Finally, we ran separate models for each of the moderators described above. To achieve this, we added interaction terms to the model between the moderators and the following: (1) time, (2) phase, and (3) the interaction between time and phase. As we ran multiple analyses for both the type of externalizing problem and treatment context, we applied Bonferroni corrections to adjust for the multiple comparisons problem.

## Results

A total of 78 SCED studies, comprising 270 cases, were included in the final multilevel analyses (overview provided in Supplements 3 and 4, available online).^33^, ^34^, ^35^, ^36^, ^37^, ^38^, ^39^, ^40^, ^41^, ^42^, ^43^, ^44^, ^45^, ^46^, ^47^, ^48^, ^49^, ^50^, ^51^, ^52^, ^53^, ^54^, ^55^, ^56^, ^57^, ^58^, ^59^, ^60^, ^61^, ^62^, ^63^, ^64^, ^65^, ^66^, ^67^, ^68^, ^69^, ^70^, ^71^, ^72^, ^73^, ^74^, ^75^, ^76^, ^77^, ^78^, ^79^, ^80^, ^81^, ^82^, ^83^, ^84^, ^85^, ^86^, ^87^, ^88^, ^89^, ^90^, ^91^, ^92^, ^93^, ^94^, ^95^, ^96^, ^97^, ^98^, ^99^, ^100^, ^101^, ^102^, ^103^, ^104^, ^105^, ^106^, ^107^, ^108^, ^109^, ^110^ The studies span the years 1968 to 2024, with 14.10% published before 1990, 14.10% in the 1990s, 12.82% in the 2000s, 38.46% in the 2010s, and 20.51% in the 2020s. This distribution indicates that single-case research has gained increasing attention over time, with a notable rise in publications during the past 2 decades. Because for the majority of studies (64.10%) follow-up data were available, we decided to include these in our final analyses. Some studies also provided more than 3 phases (eg, ABAB designs). In these cases, we decided to analyze data from only the baseline phase, (first) treatment phase, and (first) follow-up/maintenance phase. An overview of the data and syntax files can be found in Supplements 5 to 7, available online.

### Study and Case Characteristics

The included studies had an average of 4.51 cases per study (SD = 4.46). Participant age was reported on an individual level in 64.82% of studies, with a mean age of 8.70 years (SD = 3.84). The majority of participants were male (71.48% male; 17.78% female; 10.74% not reported or reported only at the study sample level). With respect to ethnicity, 16.30% of participants were identified as Afro-American, 3.33% as East Asian, 26.30% as European American, 3.70% as Latin American, and 1.48% as having mixed ethnic ancestry. Ethnicity was not reported in 48.89% of cases. Participants exhibited the following types of problem behavior: (1) aggressive behavior (37.41%), (2) antisocial behavior (1.48%), (3) noncompliant behavior (23.33%), (4) oppositional and stubborn behavior (21.11%), (5) quick-tempered and irritable behavior (9.63%), and (6) other problems (7.04%). Problem behaviors were most often identified through direct observation (72.59%), followed by parent report (18.89%) and teacher report (8.52%). It was reported that most participants experienced multiple co-occurring behavioral problems; comorbidities were explicitly reported in 18.15% of cases. The vast majority of included studies (85.9%) originated from North America and Europe, with 6.4% originating from Asia and the other 7.7% from other regions. Treatment length ranged from 4 days (eg, brief component evaluations) to 7 months (eg, long-term physical training exercises), with an average duration of 9.43 weeks. The included interventions consisted primarily of behavioral and cognitive–behavioral approaches, including parent behavior training, behavior modification, cognitive–behavioral therapy, and social skills training, sometimes combined with self-monitoring or mindfulness techniques. In addition, a smaller number of studies evaluated relationship-based, conjoint behavioral, and body-oriented (eg, haptotherapy) interventions. Overall, average scores for the total RoBiNT scale and for the internal and external validity subscales were 14.55 (SD = 3.69; score range 8-23), 4.94 (SD = 2.35; score range 1-12), and 9.61 (SD = 2.06; score range 5-15), respectively. An overview of the study and case characteristics can be found in Table 1.

**Table 1:** Characteristics of the Included Studies and Cases

	No. of studies	No. of cases
Geographical origin of studies		
North America	60	215
South America	2	7
Europe	7	12
Asia	5	19
Africa	1	9
Unknown	3	8
Study setting		
Clinical	21	48
Home	13	41
School	41	172
Home and School	3	9
Type of SCED		
Multiple-baseline	53	209
A-B	10	25
A-B-C	3	8
A-B-A	1	3
A-B-A-B	6	14
A-B-C-A	1	3
A-B-A-C	2	3
Other designs	2	5
Treatment provider		
Clinician	36	102
Caregiver	14	41
Teacher	25	120
Teacher + clinician	2	4
Teacher + caregiver	1	3
Treatment target		
Child	56	209
Teacher	1	6
Caregiver + child	20	52
Caregiver + teacher	1	3
Type of problem behaviors		
Quick tempered and irritable	9	26
Aggressive	29	101
Non-compliant	16	63
Oppositional and stubborn	15	57
Antisocial	3	4
Other behaviors	6	19
Type of outcome variable		
Observation	53	197
Reported	25	73

Note: SCED = single-case experimental design.

### Within-Person Change of Symptoms

#### Average Treatment Effects

To obtain the average treatment effects, we ran a 3-level regression analysis — providing the best model fit after sensitivity analyses — over a total of 8,977 datapoints, which were nested in 270 cases from 78 studies. We thereby specified both change in trend and change in level between the baseline and treatment phases and between the treatment and follow-up phases. The outcomes indicated that, across cases and studies, scores were on average stable in the baseline phase (b = 0.00, SE = 0.01, 95% CI = − 0.01 to 0.01, *p* = .828). At the beginning of the treatment phase, symptom severity immediately reduced (b = −1.39, SE = 0.17, 95% CI = − 1.72 to −1.06, *p* <.001). Symptom severity further reduced during the treatment phase by 0.05 SDs per day (SE = 0.01, 95% CI = − 0.07 to −0.03, *p* < .001). Symptoms did not further reduce after the treatment. In fact, the treatment effects tended to fade out, as indicated by the 0.04 increase in SDs per day during the follow-up phase, relative to the treatment phase (SE = 0.01, 95% CI = 0.02 to 0.06, *p* < .001). Finally, there was no significant immediate change in symptom severity between the treatment phase and follow-up phase (b = −0.54, SE = 0.29, 95% CI = −1.11 to 0.03, *p =* .065).

#### Heterogeneity Among Cases and Studies

**Figure 2:**
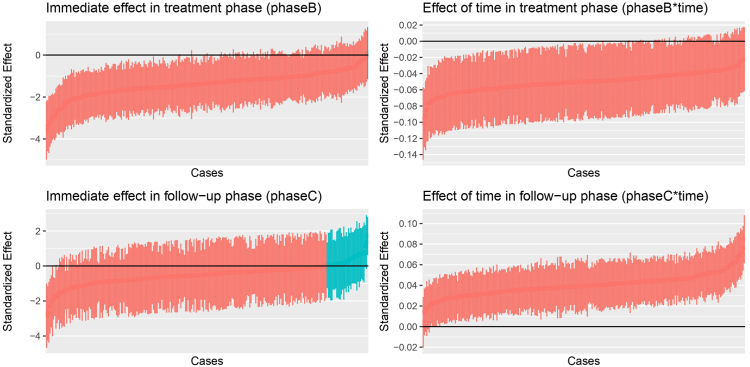
Case-Level Predictor Outcomes ***Note:****Each dot represents a case-level empirical Bayes estimate, with the lines representing their 95% confidence intervals. Estimates shown in red indicate a negative (ie, decreasing) case-level effect estimate, whereas estimates shown in turquoise indicate a positive (ie, increasing) case-level effect estimate.*

**Figure 3:**
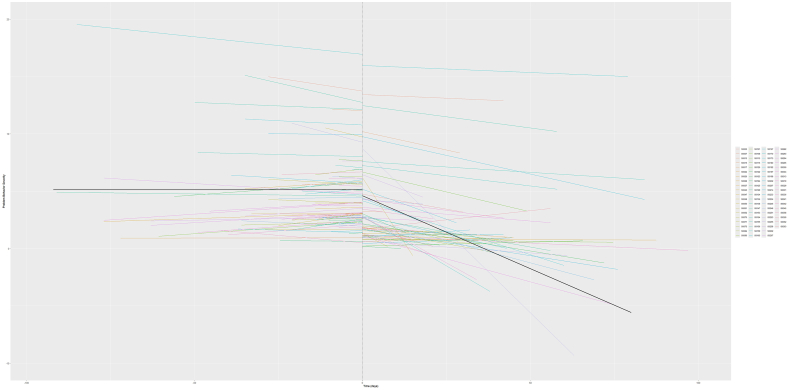
Study-Level Estimates of the Model for the Baseline and Treatment Phases ***Note:****Black lines represent the average effects across all studies, with time < 0 representing the baseline phase and time > 0 representing the treatment phase. The drop at time = 0 indicates the immediate treatment effect. Estimates are computed using the empirical Bayes method.*

The estimated treatment and follow-up effects for each case are displayed in Figure 2, and the baseline and treatment effects for each study are depicted in Figure 3. As is further supported by the model outcomes (Supplement 7, available online), the variation in intercepts within cases (σ = 1.00) is notably lower than among cases (with SD τ = 1.30) and among studies (τ = 4.68). Especially the latter highlights that there were major differences in the degree of symptom severity at the start of the treatment phase among studies. We furthermore observed that there was notable variation in treatment and follow-up effects among cases and studies. The immediate treatment effect (ie, the phaseB effect) varied with a factor of τ = 0.83 among cases and a factor of τ = 1.26 among studies. For the follow-up phase, the immediate effect (ie, phaseC) also varied slightly larger among studies (τ = 1.79) compared to the variation among cases (τ = 1.07). The variation in the effect of time during the baseline, its change during the treatment phase (ie, time∗phaseB), and its change during the follow-up phase (ie, time∗phaseC) each showed less variation (τ < 0.08) among cases and studies.

Despite the variation in computed estimates among cases, Figure 2 shows that nearly all cases had an immediate and gradual reduction in symptoms in the treatment phase. At follow-up, 87.10% of cases had a decrease in the individuals’ symptoms. The gradual change of symptom severity over time during the follow-up phase, however, showed a stable increasing effect for all cases. Further inspections of the case-and study-level correlations were also conducted. Across studies, we found a large negative correlation of *r* = −0.92 between the random change in trend during the treatment phase and random change in trend during the follow-up phase. This implies that the studies with steeper declines in symptoms throughout the treatment phase show a greater additive increasing effect throughout the follow-up phase. At the case-level, we observed a large positive correlation (*r* = 0.63) between the random intercept and random change in trend in the follow-up phase. Thus, for cases of individuals with more severe symptoms at the end of the baseline, the positive change in trend during the follow-up was larger. Finally, we found large negative correlations between the random immediate effect and change in trend for both the treatment phase (*r* = −0.73) and follow-up phase (*r* = −0.77). This indicates that cases with generally higher immediate effects showed less change throughout the phases.

#### Treatment Moderators

We also performed multiple 3-level regression analyses including moderators, to examine differences among the types of externalizing problems, treatment contexts, and form of assessment. Studies that used observational data had a stronger immediate effect during the treatment phase compared to those using report data (b = −1.23, SE = 0.35, 95% CI = − 1.91 to −0.55, *p* < .001). In addition, studies using observational data also showed a steeper declining trend during the treatment phase when compared with data reported by teachers, parents, and children themselves (b = −0.05, SE = 0.02, 95% CI = − 0.08 to −0.01, *p* = .014). We did not observe any significant differences for the follow-up phase. None of the treatment context variables (eg, school or home) significantly predicted the variation of each treatment effects from the non-moderated model (phase, time, and their interactions). We hereby accounted for the multiple comparisons using Bonferroni corrections (ie, an adapted significance level of 0.05/6 = 0.008 for treatment setting and 0.05/15 = 0.003 for type of problem behavior). Likewise, treatment and follow-up effects were similar for the different types of externalizing problem behavior. A full display of the results from these analyses can be found in Supplement 8, available online.

## Discussion

This is, to our knowledge, the first overall multilevel meta-analytic study that aims to analyze the results from published single-case research in children and adolescents with externalizing behavior problems. We found that, following a stable baseline, symptoms were reduced during treatment. However, participants who showed a stronger immediate symptom reduction during treatment were less likely to show continued improvement over time. Variations in treatment effects among the studies were large, and those among the cases were modest. Symptom reduction did not carry through during the follow-up phase. In fact, we observed that the treatment effect tended to fade out during the follow-up phase. Of the 3 moderators tested, only the type of assessment method (observation vs report) moderated treatment outcomes. We did not distinguish between parent and teacher reports, as both reflect proxy informant evaluations of child behavior rather than self-report or direct observation. Given their conceptual similarity and their relatively small proportion in the dataset (less than 30% of assessments), we analyzed them jointly as a single “report” category. Treatment effects were larger for observational than for reported data.

Our research hence indicates that, overall, interventions as studied in single-case research in youth with externalizing behavior problems effectively reduce externalizing problems during the treatments. This finding corresponds to those from between-group studies (ie, RCTs).^111^^,^^112^ At the same time, and similar to meta-analyses of RCTs, variability in treatment effects among the studies was large, pointing to the need to continue using idiosyncratic (ie, single-case design and data analyses) methods to learn about these treatment effects. We observed, furthermore, that most studies tested multi-component (eg, combing behavioral modification therapy and self-monitoring, or parent behavior training and mindfulness therapy) interventions while targeting specific symptoms such as aggression or disobedience. This is similar to interventions evaluated in RCTs.^113^ When adopting a single-case experimental design, however, we might expect a more personalized approach (eg, selectively using components such as time-out procedures or reward systems). Personalization seems to be implemented in several studies mainly through means of delivery of treatment (for example, the use of video conferencing, modeling, and storyteling).^34^^,^^36^^,^^39^^,^^50^

Overall, no consistent further decreases were found during the follow-up phase, indicating that in the absence of an intervention, and in other contexts, it seems difficult to retain treatment gains (ie, decreases in symptom severity) during the follow-up phase, reinforcing the idea that, in the absence of an intervention, it is difficult to retain treatment gains over time. These findings underscore the importance of ongoing support or intervention to maintain treatment effects. Moreover, these findings suggest that, without professional intervention or guidance (such as from clinicians or intervention coaches), parents or teachers may struggle to maintain behavioral improvements. In addition, the limited data available for our follow-up analysis may have affected our results, which should be considered when interpreting these findings.

In terms of moderators, we found evidence for only the type of assessment method. Interventions in studies that used observational methods seemed to be more effective than interventions in studies that used data reported by parents, teachers, or children themselves. Although this result can be explained in a methodological way (eg, a lower degree of assessment bias), it is also possible that changes in the externalizing symptoms can better be depicted during frequent observations of a specific behavioral target, as situations during which the children are observed look like the situations during which the intervention was offered (eg, compliance training during play). These measurements might therefore reveal treatment effects in a more accurate way.^111^ The fact that we did not find any effects for the moderator “type of externalizing problem” is perhaps surprising. At the same time, it is plausible that single-case interventions were tailored to specific problems and therefore were similarly effective. In this line, it would be interesting to address comorbid diagnoses and medication use, as these have been well documented in the literature.^114^^,^^115^ Although such information was available for some cases in our meta-analysis, this portion still made up 16.3% of the total sample. We acknowledge that this might be a characteristic of the current state of single-case research on the topic, focusing more on self-contained instances of externalizing problems. However, as we further argue that the absence of a moderating effect for problem type could also be allocated to a lack of statistical power (providing that some subcategories had limited numbers), further subdividing cases at this point in time would have provided an even more severe lack of power.

Next to this issue of power, we identified a few other limitations of this study. The most important is related to the quality of the included single-case studies. We rated the studies using the RoBiNT scale that allows for the analysis of both internal and external validity.^30^ Although average scores on external validity were above the mean, internal validity scores were below the average. Information on several items of this questionnaire was frequently missing in the studies included, or from the information present it was clear that that specific criterion was not fulfilled. We were familiar with this instrument from previous studies (eg, Maric *et al.*^116^), and, although we acknowledge that not all quality indicators may be easy to achieve in clinical youth research, we do believe that lack of information about “blinding of assessors” or “treatment adherence” diminishes the confidence with which we can draw conclusions about positive effects of treatments for externalizing problems as studied in single-case research. Another important limitation relates to the lack of information about the diversity of the clients who participated in specific studies. For example, in many studies, ethnicity was underreported, or ethnicity and race were used interchangeably.

Following the above, we recommend that, in future research, the SCED reporting guidelines by Tate *et al.*^117^ be applied when designing and conducting single-case research in youth. High-quality SCED studies will help us to understand treatment effects in the best possible way and will allow us to draw conclusions that are better grounded. This is especially important in cases where findings are implemented in regular clinical practice. Our second recommendation is to work toward a standardized method of reporting and classifying externalizing behavior problems. As mentioned above, we observed notable differences among studies in the operationalization of externalizing behavior. Furthermore, it is beyond doubt whether, based on the descriptions, cases in these studies fulfill diagnostic criteria for externalizing disorders. However, clinical problems remain described at the behavioral problem levels and not in terms of diagnostic symptoms. Providing guidelines for both the scientific and clinical fields may help to reduce these discrepancies. Finally, we want to highlight that further research into moderating factors in single-case studies on externalizing behavior problems is vital, given the high heterogeneity in this group of children and adolescents. Based on the moderating result regarding observational vs report methods, clinical practice would greatly benefit from knowledge and guidelines concerning measurement- vs observational-based care.

In conclusion, we have presented what is, to our knowledge, the first comprehensive meta-analytical review of published single-case studies on externalizing behavior problems. Using a 3-level multilevel approach, we found that treatment for these problems yields promising results. At the same time, however, we acknowledge that more research is necessary to uncover the factors that underlie the substantial variability among studies and cases. Despite this, we are hopeful that single-case studies can play a role in further untangling individual differences, and thereby in tailoring personalized interventions.

## CRediT authorship contribution statement

**Shawn I. Kok:** Writing – review & editing, Writing – original draft, Visualization, Validation, Software, Project administration, Methodology, Formal analysis, Data curation, Conceptualization. **Laura M. Fetz:** Writing – review & editing, Writing – original draft, Visualization, Validation, Resources, Project administration, Methodology, Investigation, Data curation, Conceptualization. **Yvonne A.J. Stikkelbroek:** Writing – review & editing, Supervision, Investigation, Data curation, Conceptualization. **Patty Leijten:** Writing – review & editing, Supervision, Investigation, Conceptualization. **Janneke Staaks:** Software, Resources, Project administration, Methodology, Data curation. **Marija Maric:** Writing – review & editing, Writing – original draft, Supervision, Funding acquisition, Data curation, Conceptualization.
