# Supplementary material for: A Multilevel Meta-Analysis of Single-Case Research on Interventions for Externalizing Behavior Problems in Children and Adolescents
Source: JAACAP Open. 2025 Dec 18;4(2):220–31. doi: 10.1016/j.jaacop.2025.12.002 (PMC13043497; doi:10.1016/j.jaacop.2025.12.002)
Supplement: Supplemental Data [file mmc2.pdf]

Supplementary Material #1 – Literature Search Terms

PsycINFO (Ovid, APA PsycINFO, from 1806 to present)

1. (case series or multiple baseline design or n=1 design* or n=1 stud* or n=1 trial* or n-of-1 design* or n-of-1 method* or n-of-1 stud* or n-of-1 trial* or single case* exp* or single case* design* or single case* stud* or sced or sceds).ti,ab,id.
2. (preschool age 2 5 yrs OR school age 6 12 yrs or adolescence 13 17 yrs).ag. OR child psychiatry/ OR orthopsychiatry/ OR child psychopathology/ OR child psychology/ OR child psychotherapy/ OR adolescent psychiatry/ OR adolescent psychopathology/ OR adolescent psychology/ OR adolescent psychotherapy/ or (child* or kid or kids or preschool* OR puberty or pubescen* or teen* or young* or youth* or minors* or under ag* or underag* or juvenile* or girl* or boy* or preadolesc* or adolesc*).ti,ab,id.
3. acting out/ OR aggressive behavior/ OR antisocial behavior/ OR behavior disorders/ OR conduct disorder/ OR criminal behavior/ OR disruptive behavior disorders/ OR explosive disorder/ OR externalization/ OR externalizing symptoms/ OR oppositional defiant disorder/ OR rebelliousness/ OR tantrums/ OR juvenile delinquency/ OR (acting out OR aggress* OR anger OR angry OR antisocial OR antisocial* OR anti-social* OR behavi* difficult* OR conduct* problem* OR ((conduct OR behavio* OR defiant OR disruptive OR dysfunctional* OR explosiv* OR maladaptiv* OR oppositional) ADJ3 disorder*) OR ((defiant OR disruptive OR dysfunctional* OR explosiv* OR maladaptiv* OR oppositional OR problem*) ADJ3 behavio*) OR crime OR criminal* OR delinq* OR devian* OR externali* OR impulse control disorder* OR impulsiv* OR misbehavio* OR misconduct OR noncomplian* OR ODD OR offen* OR rule breaking OR tantrum*).ti,ab,id.
4. (therapy OR intervention* OR treatment* OR program* OR training OR psychotherapy).ti,ab,id.
5. 1 AND 2 AND 3 AND 4

Key: / = subject heading, ti = title, ab = abstract, id = key concepts (other keywords added by PsycINFO indexers to supplement the subject headings), ag = age group, ADJn = word distance of maximum n words

Medline (Ovid MEDLINE ALL, including Epub Ahead of Print, In-Process, In-Data-Review & Other Non-Indexed Citations and Daily, from 1946 to present)

1. single-case studies as topic/ OR (case series or multiple baseline design or n=1 design* or n=1 stud* or n=1 trial* or n-of-1 design* or n-of-1 method* or n-of-1 stud* or n-of-1 trial* or single case* exp* or single case* design* or single case* stud* or sced or sceds).ti,ab,kf.
2. child psychiatry/ OR orthopsychiatry/ OR child psychology/ OR child behavior disorders/ OR child, preschool/ OR child/ OR puberty/ OR adolescent/ OR adolescent psychiatry/ OR adolescent psychology/ OR (child* or kid or kids or preschool* OR puberty or pubescen* or teen* or young* or youth* or minors* or under ag* or underag* or juvenile* or girl* or boy* or preadolesc* or adolesc*).ti,ab,kf.
3. acting out/ OR agression/ OR conduct disorder/ OR criminal behavior/ OR problem behavior/ OR juvenile delinquency/ OR (acting out OR aggress* OR anger OR angry OR antisocial OR antisocial* OR anti-social* OR behavi* difficult* OR conduct* problem* OR ((conduct OR behavio* OR defiant OR disruptive OR dysfunctional* OR explosiv* OR maladaptiv* OR oppositional) ADJ3 disorder*) OR ((defiant OR disruptive OR

dysfunctional* OR explosiv* OR maladaptiv* OR oppositional OR problem*) ADJ3 behavio*) OR crime OR criminal* OR delinq* OR devian* OR externali* OR impulsiv* OR misbehavio* OR misconduct OR noncomplian* OR ODD OR offen* OR rule breaking OR tantrum*).ti,ab,kf.
4. (therapy OR intervention* OR treatment* OR program* OR training OR psychotherapy).ti,ab,kf.
5. 1 AND 2 AND 3 AND 4

Key: / = medical subject heading (MeSH), ti = title, ab = abstract, kf = author supplied keywords, ADJn = word distance of maximum n words

Web of Science Core Collection (Web of Science Core Collection Editions: Science Citation Index Expanded (SCI-EXPANDED), 1975 - present, Social Sciences Citation Index (SSCI), 1975 - present, Arts & Humanities Citation Index (A&HCI), 1975 - present, Emerging Sources Citation Index (ESCI), 2005 - present))

1. TS=("case series" OR "multiple baseline design" OR "n=1 design*" OR "n=1 stud*" OR "n=1 trial*" OR "n- of-1 design*" OR "n-of-1 method*" OR "n-of-1 stud*" OR "n-of-1 trial*" OR "single case* exp*" OR "single case* design*" OR "single case* stud*" OR "sced" OR "sceds")
2. TS=("child*" OR "kid" OR "kids" OR "preschool" OR "puberty" OR "pubescen*" OR "teen*" OR "young*" OR "youth*" OR "minors*" OR "under ag*" OR "underag*" OR "juvenile*" OR "girl*" OR "boy*" OR "preadolesc*" OR "adolesc*")
3. TS=("acting out" OR "aggress*" OR "anger" OR "angry" OR "antisocial" OR "antisocial*" OR "anti-social*" OR "behavi* difficult*" OR "conduct* problem*" OR (("conduct" OR "behavio*" OR "defiant" OR "disruptive" OR "dysfunctional*" OR "explosiv*" OR "maladaptiv*" OR "oppositional") NEAR/2 "disorder*") OR (("defiant" OR "disruptive" OR "dysfunctional*" OR "explosiv*" OR "maladaptiv*" OR "oppositional" OR "problem*") NEAR/2 "behavio*") OR "crime" OR "criminal*" OR "delinq*" OR "devian*" OR "externali*" OR "impulsiv*" OR "misbehavio*" OR "misconduct" OR "noncomplian*" OR "ODD" OR "offen*" OR "rule breaking" OR "tantrum*")
4. TS=("therapy" OR "intervention*" OR "treatment*" OR "program*" OR "training" OR "psychotherapy")
5. #1 AND #2 AND #3 AND #4

Key: TS = topic, which includes title, abstract, author keywords and Web of Science Keywords Plus, NEAR/n = word distance of maximum n words
