# Supplementary material for: A Multilevel Meta-Analysis of Single-Case Research on Interventions for Externalizing Behavior Problems in Children and Adolescents
Source: JAACAP Open. 2025 Dec 18;4(2):220–31. doi: 10.1016/j.jaacop.2025.12.002 (PMC13043497; doi:10.1016/j.jaacop.2025.12.002)
Supplement: Supplemental Data [file mmc5.pdf]

# Supplement #7 – Complete Model Outcomes from R

December 2025

## Outcomes of the Model Without Moderators

```
## Linear mixed model fit by REML ['lmerMod']
## Formula: outcome_std ~ 1 + s.time_centered + phaseB + s.time_centered *
##     phaseB + phaseC + s.time_centered * phaseC + (1 + s.time_centered +
##     phaseB + s.time_centered * phaseB + phaseC + s.time_centered *
##     phaseC | study_id/child_id)
##    Data: GraphData
## Control: lmerControl(optimizer = "bobyqa", optCtrl = list(maxfun = 5e+05))
##
## REML criterion at convergence: 23426.8
##
## Scaled residuals:
##     Min      1Q  Median      3Q     Max
## -3.9976 -0.5604 -0.1055  0.4200  6.3471
##
## Random effects:
##  Groups            Name                   Variance  Std.Dev. Corr
##  child_id:study_id (Intercept)            1.680e+00 1.29605
##                    s.time_centered        4.180e-04 0.02045   0.09
##                    phaseB                 6.838e-01 0.82690  -0.41 -0.40
##                    phaseC                 1.155e+00 1.07476  -0.21  0.56 -0.13
##                    s.time_centered:phaseB 6.084e-04 0.02467  -0.42 -0.73  0.33
##                    s.time_centered:phaseC 2.332e-04 0.01527   0.63 -0.23 -0.12
##  study_id          (Intercept)            2.188e+01 4.67737
##                    s.time_centered        1.028e-03 0.03206  -0.45
##                    phaseB                 1.583e+00 1.25806  -0.03 -0.20
##                    phaseC                 3.212e+00 1.79209  -0.05 -0.25 -0.23
##                    s.time_centered:phaseB 4.521e-03 0.06724  -0.08  0.01  0.35
##                    s.time_centered:phaseC 4.928e-03 0.07020   0.18 -0.37 -0.21
##  Residual                                 9.921e-01 0.99606
##
##
##
##
##
##  -0.17
##  -0.77 -0.39
##
##
##
##
##   0.46
##  -0.43 -0.92
```

```
##
## Number of obs: 7410, groups:  child_id:study_id, 271; study_id, 78
##
## Fixed effects:
##                          Estimate Std. Error t value
## (Intercept)              5.124317   0.540904   9.474
## s.time_centered         -0.001208   0.005551  -0.218
## phaseB                  -1.388187   0.167111  -8.307
## phaseC                  -0.538053   0.291182  -1.848
## s.time_centered:phaseB -0.049839   0.009174  -5.433
## s.time_centered:phaseC  0.040352   0.008790   4.590
##
## Correlation of Fixed Effects:
##             (Intr) s.tm_c phaseB phaseC s.t_:B
## s.tim_cntrd -0.237
## phaseB      -0.080 -0.307
## phaseC      -0.042 -0.063 -0.175
## s.tm_cntr:B -0.107 -0.308  0.318  0.251
## s.tm_cntr:C  0.167 -0.267 -0.117 -0.422 -0.791
## optimizer (bobyqa) convergence code: 0 (OK)
## boundary (singular) fit: see help('isSingular')
```

### Outcomes of the Model With Assesment Method as Moderator (0 = reports)

```
## Linear mixed model fit by REML ['lmerMod']
## Formula:
## outcome_std ~ 1 + s.time_centered * phaseB * method + s.time_centered *
##     phaseC * method + (1 + s.time_centered + phaseB + s.time_centered *
##     phaseB + phaseC + s.time_centered * phaseC | study_id/child_id)
##    Data: GraphData
## Control: lmerControl(optimizer = "bobyqa", optCtrl = list(maxfun = 5e+05))
##
## REML criterion at convergence: 23330.5
##
## Scaled residuals:
##     Min      1Q  Median      3Q     Max
## -3.9976 -0.5594 -0.1067  0.4191  6.3436
##
## Random effects:
##  Groups            Name                   Variance  Std.Dev. Corr
##  child_id:study_id (Intercept)            1.683e+00 1.29713
##                    s.time_centered        4.396e-04 0.02097   0.10
##                    phaseB                 6.757e-01 0.82200  -0.41 -0.38
##                    phaseC                 1.165e+00 1.07945  -0.22  0.51 -0.12
##                    s.time_centered:phaseB 6.417e-04 0.02533  -0.42 -0.75  0.31
##                    s.time_centered:phaseC 2.434e-04 0.01560   0.63 -0.18 -0.13
##  study_id          (Intercept)            2.083e+01 4.56441
##                    s.time_centered        7.745e-04 0.02783  -0.35
##                    phaseB                 1.367e+00 1.16907  -0.20 -0.03
##                    phaseC                 3.270e+00 1.80843  -0.04 -0.33 -0.23
##                    s.time_centered:phaseB 4.160e-03 0.06450  -0.19  0.22  0.25
##                    s.time_centered:phaseC 5.198e-03 0.07210   0.20 -0.48 -0.17
##  Residual                                 9.917e-01 0.99585
```

```
##
##
##
##
##
##  -0.13
##  -0.78 -0.42
##
##
##
##
##   0.53
##  -0.44 -0.95
##
## Number of obs: 7381, groups:  child_id:study_id, 270; study_id, 77
##
## Fixed effects:
##                               Estimate Std. Error t value
## (Intercept)                    6.99866    0.93781   7.463
## s.time_centered               -0.02301    0.00879  -2.617
## phaseB                        -0.53318    0.29004  -1.838
## method                        -2.74298    1.13841  -2.409
## phaseC                        -0.66132    0.55706  -1.187
## s.time_centered:phaseB        -0.01925    0.01515  -1.271
## s.time_centered:method         0.03081    0.01081   2.850
## phaseB:method                 -1.22900    0.34656  -3.546
## s.time_centered:phaseC         0.03122    0.01554   2.009
## method:phaseC                  0.17146    0.65485   0.262
## s.time_centered:phaseB:method -0.04584    0.01871  -2.450
## s.time_centered:method:phaseC  0.01603    0.01915   0.837
##
## Correlation of Fixed Effects:
##             (Intr) s.tm_c phaseB method phaseC s.t_:B s.tm_: phsB:m s.t_:C
## s.tim_cntrd -0.162
## phaseB      -0.237 -0.228
## method      -0.824  0.133  0.195
## phaseC      -0.037 -0.144 -0.154  0.030
## s.tm_cntr:B -0.200 -0.202  0.251  0.165  0.316
## s.tm_cntrd:  0.132 -0.813  0.186 -0.162  0.117  0.164
## phaseB:mthd  0.198  0.191 -0.837 -0.232  0.129 -0.210 -0.224
## s.tm_cntr:C  0.195 -0.319 -0.092 -0.161 -0.435 -0.819  0.260  0.077
## method:phsC  0.031  0.123  0.131 -0.037 -0.851 -0.269 -0.121 -0.162  0.370
## s.tm_cnt:B:  0.162  0.163 -0.203 -0.196 -0.256 -0.810 -0.204  0.244  0.663
## s.tm_cnt::C -0.158  0.259  0.075  0.192  0.353  0.664 -0.319 -0.089 -0.811
##             mthd:C s._:B:
## s.tim_cntrd
## phaseB
## method
## phaseC
## s.tm_cntr:B
## s.tm_cntrd:
## phaseB:mthd
## s.tm_cntr:C
## method:phsC
```

```
## s.tm_cnt:B:  0.308
## s.tm_cnt::C -0.433 -0.818
## optimizer (bobyqa) convergence code: 0 (OK)
## boundary (singular) fit: see help('isSingular')
```

### Outcomes of the Model With Treatment Context as Moderator (0 = clinical/hospital setting)

```
## Linear mixed model fit by REML ['lmerMod']
## Formula:
## outcome_std ~ 1 + s.time_centered * phaseB * setting_home + s.time_centered *
##     phaseC * setting_home + s.time_centered * phaseB * setting_school +
##     s.time_centered * phaseC * setting_school + s.time_centered *
##     phaseB * setting_home_and_school + s.time_centered * phaseC *
##     setting_home_and_school + (1 + s.time_centered + phaseB +
##     s.time_centered * phaseB + phaseC + s.time_centered * phaseC |
##     study_id/child_id)
##    Data: GraphData
## Control: lmerControl(optimizer = "bobyqa", optCtrl = list(maxfun = 5e+05))
##
## REML criterion at convergence: 23345.4
##
## Scaled residuals:
##     Min      1Q  Median      3Q     Max
## -4.0048 -0.5598 -0.1069  0.4227  6.3467
##
## Random effects:
##  Groups            Name                   Variance  Std.Dev. Corr
##  child_id:study_id (Intercept)            1.664e+00 1.29001
##                    s.time_centered        4.345e-04 0.02085   0.07
##                    phaseB                 6.824e-01 0.82610  -0.41 -0.39
##                    phaseC                 1.043e+00 1.02141  -0.17  0.50 -0.15
##                    s.time_centered:phaseB 5.980e-04 0.02445  -0.40 -0.73  0.31
##                    s.time_centered:phaseC 2.165e-04 0.01471   0.62 -0.22 -0.11
##  study_id          (Intercept)            1.970e+01 4.43845
##                    s.time_centered        1.010e-03 0.03177  -0.31
##                    phaseB                 1.626e+00 1.27510  -0.12 -0.19
##                    phaseC                 3.137e+00 1.77103   0.00 -0.20 -0.28
##                    s.time_centered:phaseB 4.610e-03 0.06790  -0.18  0.06  0.35
##                    s.time_centered:phaseC 4.498e-03 0.06707   0.18 -0.42 -0.18
##  Residual                                 9.918e-01 0.99590
##
##
##
##
##
##  -0.16
##  -0.74 -0.40
##
##
##
##
##   0.38
```

```
##  -0.40 -0.91
##
## Number of obs: 7381, groups:  child_id:study_id, 270; study_id, 77
##
## Fixed effects:
##                                                Estimate Std. Error t value
## (Intercept)                                    7.820977   1.035147   7.555
## s.time_centered                                0.002731   0.013777   0.198
## phaseB                                        -1.425797   0.377999  -3.772
## setting_home                                   5.859814   4.608966   1.271
## phaseC                                        -1.613341   0.677384  -2.382
## setting_school                                -3.878065   1.252345  -3.097
## setting_home_and_school                       -3.207148   1.561852  -2.053
## s.time_centered:phaseB                        -0.060610   0.019896  -3.046
## s.time_centered:setting_home                  -0.062012   0.042786  -1.449
## phaseB:setting_home                            1.224602   1.432198   0.855
## s.time_centered:phaseC                         0.051038   0.016547   3.084
## setting_home:phaseC                           -4.529064   7.811883  -0.580
## s.time_centered:setting_school                 0.001659   0.015695   0.106
## phaseB:setting_school                         -0.127603   0.438977  -0.291
## phaseC:setting_school                          1.589997   0.774513   2.053
## s.time_centered:setting_home_and_school       -0.010751   0.017968  -0.598
## phaseB:setting_home_and_school                 0.330746   0.535453   0.618
## phaseC:setting_home_and_school                 0.820719   0.980220   0.837
## s.time_centered:phaseB:setting_home            0.114109   0.079095   1.443
## s.time_centered:setting_home:phaseC            0.110280   0.208605   0.529
## s.time_centered:phaseB:setting_school          0.001151   0.023680   0.049
## s.time_centered:phaseC:setting_school         -0.013533   0.020391  -0.664
## s.time_centered:phaseB:setting_home_and_school  0.025928   0.028167   0.921
## s.time_centered:phaseC:setting_home_and_school -0.019905   0.025034  -0.795
```

```
##
## Correlation matrix not shown by default, as p = 24 > 12.
## Use print(x, correlation=TRUE)  or
##     vcov(x)        if you need it
```

```
## optimizer (bobyqa) convergence code: 0 (OK)
## boundary (singular) fit: see help('isSingular')
```

### Outcomes of the Model With Treatment Context as Moderator (0 = home)

```
## Linear mixed model fit by REML ['lmerMod']
## Formula: outcome_std ~ 1 + s.time_centered * phaseB * setting_clinic +
##     s.time_centered * phaseC * setting_clinic + s.time_centered *
##     phaseB * setting_school + s.time_centered * phaseC * setting_school +
##     s.time_centered * phaseB * setting_home_and_school + s.time_centered *
##     phaseC * setting_home_and_school + (1 + s.time_centered +
##     phaseB + s.time_centered * phaseB + phaseC + s.time_centered *
##     phaseC | study_id/child_id)
##    Data: GraphData
## Control: lmerControl(optimizer = "bobyqa", optCtrl = list(maxfun = 5e+05))
##
## REML criterion at convergence: 23345.4
```

```
##
## Scaled residuals:
##     Min      1Q  Median      3Q     Max
## -4.0048 -0.5598 -0.1069  0.4227  6.3467
##
## Random effects:
##  Groups            Name                   Variance  Std.Dev. Corr
##  child_id:study_id (Intercept)            1.664e+00 1.29001
##                    s.time_centered        4.345e-04 0.02085   0.07
##                    phaseB                 6.824e-01 0.82610  -0.41 -0.39
##                    phaseC                 1.043e+00 1.02143  -0.17  0.50 -0.15
##                    s.time_centered:phaseB 5.980e-04 0.02445  -0.40 -0.73  0.31
##                    s.time_centered:phaseC 2.165e-04 0.01471   0.62 -0.22 -0.11
##  study_id          (Intercept)            1.970e+01 4.43849
##                    s.time_centered        1.010e-03 0.03177  -0.31
##                    phaseB                 1.626e+00 1.27517  -0.12 -0.19
##                    phaseC                 3.137e+00 1.77109   0.00 -0.20 -0.28
##                    s.time_centered:phaseB 4.610e-03 0.06790  -0.18  0.06  0.35
##                    s.time_centered:phaseC 4.498e-03 0.06707   0.18 -0.42 -0.18
##  Residual                                 9.918e-01 0.99590
##
##
##
##
##
##  -0.16
##  -0.74 -0.40
##
##
##
##
##   0.38
##  -0.40 -0.91
##
## Number of obs: 7381, groups:  child_id:study_id, 270; study_id, 77
##
## Fixed effects:
##                                            Estimate Std. Error t value
## (Intercept)                                13.68079    4.49126   3.046
## s.time_centered                            -0.05928    0.04051  -1.463
## phaseB                                     -0.20119    1.38147  -0.146
## setting_clinic                             -5.85976    4.60901  -1.271
## phaseC                                     -6.14230    7.78250  -0.789
## setting_school                             -9.73784    4.54624  -2.142
## setting_home_and_school                    -9.06697    4.64105  -1.954
## s.time_centered:phaseB                      0.05350    0.07655   0.699
## s.time_centered:setting_clinic              0.06202    0.04279   1.449
## phaseB:setting_clinic                      -1.22465    1.43226  -0.855
## s.time_centered:phaseC                      0.16132    0.20795   0.776
## setting_clinic:phaseC                       4.52889    7.81192   0.580
## s.time_centered:setting_school              0.06368    0.04120   1.546
## phaseB:setting_school                      -1.35224    1.39939  -0.966
## phaseC:setting_school                       6.11901    7.79155   0.785
## s.time_centered:setting_home_and_school     0.05126    0.04212   1.217
```

```
## phaseB:setting_home_and_school                 -0.89385    1.43259  -0.624
## phaseC:setting_home_and_school                  5.34991    7.81469   0.685
## s.time_centered:phaseB:setting_clinic          -0.11412    0.07910  -1.443
## s.time_centered:setting_clinic:phaseC          -0.11028    0.20861  -0.529
## s.time_centered:phaseB:setting_school          -0.11297    0.07762  -1.455
## s.time_centered:phaseC:setting_school          -0.12381    0.20829  -0.594
## s.time_centered:phaseB:setting_home_and_school -0.08818    0.07911  -1.115
## s.time_centered:phaseC:setting_home_and_school -0.13018    0.20880  -0.624

##
## Correlation matrix not shown by default, as p = 24 > 12.
## Use print(x, correlation=TRUE)  or
##     vcov(x)        if you need it

## optimizer (bobyqa) convergence code: 0 (OK)
## boundary (singular) fit: see help('isSingular')
```

### Outcomes of the Model With Treatment Context as Moderator (0 = school)

```
## Linear mixed model fit by REML ['lmerMod']
## Formula: outcome_std ~ 1 + s.time_centered * phaseB * setting_clinic +
##     s.time_centered * phaseC * setting_clinic + s.time_centered *
##     phaseB * setting_home + s.time_centered * phaseC * setting_home +
##     s.time_centered * phaseB * setting_home_and_school + s.time_centered *
##     phaseC * setting_home_and_school + (1 + s.time_centered +
##     phaseB + s.time_centered * phaseB + phaseC + s.time_centered *
##     phaseC | study_id/child_id)
##    Data: GraphData
## Control: lmerControl(optimizer = "bobyqa", optCtrl = list(maxfun = 5e+05))
##
## REML criterion at convergence: 23345.4
##
## Scaled residuals:
##     Min      1Q  Median      3Q     Max
## -4.0048 -0.5598 -0.1069  0.4227  6.3467
##
## Random effects:
##  Groups            Name                   Variance  Std.Dev. Corr
##  child_id:study_id (Intercept)            1.664e+00 1.29000
##                    s.time_centered        4.345e-04 0.02085   0.07
##                    phaseB                 6.825e-01 0.82611  -0.41 -0.39
##                    phaseC                 1.043e+00 1.02139  -0.17  0.50 -0.15
##                    s.time_centered:phaseB 5.980e-04 0.02445  -0.40 -0.73  0.31
##                    s.time_centered:phaseC 2.165e-04 0.01471   0.62 -0.22 -0.11
##  study_id          (Intercept)            1.970e+01 4.43854
##                    s.time_centered        1.010e-03 0.03177  -0.31
##                    phaseB                 1.626e+00 1.27511  -0.12 -0.19
##                    phaseC                 3.137e+00 1.77108   0.00 -0.20 -0.28
##                    s.time_centered:phaseB 4.610e-03 0.06790  -0.18  0.06  0.35
##                    s.time_centered:phaseC 4.498e-03 0.06707   0.18 -0.42 -0.18
##  Residual                                 9.918e-01 0.99590
##
##
```

```
##
##
##
##  -0.16
##  -0.74 -0.40
##
##
##
##
##   0.38
##  -0.40 -0.91
##
## Number of obs: 7381, groups:  child_id:study_id, 270; study_id, 77
##
## Fixed effects:
##                                               Estimate Std. Error t value
## (Intercept)                                   3.942916   0.704882   5.594
## s.time_centered                               0.004392   0.007517   0.584
## phaseB                                       -1.553405   0.223200  -6.960
## setting_clinic                                3.878076   1.252369   3.097
## phaseC                                       -0.023326   0.375539  -0.062
## setting_home                                  9.737874   4.546285   2.142
## setting_home_and_school                       0.670913   1.365564   0.491
## s.time_centered:phaseB                       -0.059461   0.012842  -4.630
## s.time_centered:setting_clinic               -0.001659   0.015695  -0.106
## phaseB:setting_clinic                         0.127594   0.438979   0.291
## s.time_centered:phaseC                        0.037505   0.011915   3.148
## setting_clinic:phaseC                        -1.589983   0.774529  -2.053
## s.time_centered:setting_home                 -0.063672   0.041199  -1.545
## phaseB:setting_home                           1.352210   1.399335   0.966
## phaseC:setting_home                          -6.119634   7.791516  -0.785
## s.time_centered:setting_home_and_school      -0.012411   0.013768  -0.901
## phaseB:setting_home_and_school                0.458354   0.440053   1.042
## phaseC:setting_home_and_school               -0.769252   0.801903  -0.959
## s.time_centered:phaseB:setting_clinic        -0.001151   0.023681  -0.049
## s.time_centered:setting_clinic:phaseC         0.013532   0.020391   0.664
## s.time_centered:phaseB:setting_home           0.112959   0.077622   1.455
## s.time_centered:phaseC:setting_home           0.123827   0.208289   0.594
## s.time_centered:phaseB:setting_home_and_school  0.024778   0.023716   1.045
## s.time_centered:phaseC:setting_home_and_school -0.006372   0.022245  -0.286
```

```
##
## Correlation matrix not shown by default, as p = 24 > 12.
## Use print(x, correlation=TRUE)  or
##     vcov(x)        if you need it
```

```
## optimizer (bobyqa) convergence code: 0 (OK)
## boundary (singular) fit: see help('isSingular')
```

## Outcomes of the Model With Type of Problem Behavior as Moderator (0 = tempered/irritable)

```
## Linear mixed model fit by REML ['lmerMod']
```

```
## Formula: outcome_std ~ 1 + s.time_centered * phaseB * problem_aggressive +
##     s.time_centered * phaseC * problem_aggressive + s.time_centered *
##     phaseB * problem_anti_social + s.time_centered * phaseC *
##     problem_anti_social + s.time_centered * phaseB * problem_noncompliance +
##     s.time_centered * phaseC * problem_noncompliance + s.time_centered *
##     phaseB * problem_oppositional_stubborn + s.time_centered *
##     phaseC * problem_oppositional_stubborn + s.time_centered *
##     phaseB * problem_other + s.time_centered * phaseC * problem_other +
##     (1 + s.time_centered + phaseB + s.time_centered * phaseB +
##         phaseC + s.time_centered * phaseC | study_id/child_id)
##    Data: GraphData
## Control: lmerControl(optimizer = "bobyqa", optCtrl = list(maxfun = 5e+05))
##
## REML criterion at convergence: 23404.7
##
## Scaled residuals:
##     Min      1Q  Median      3Q     Max
## -4.0055 -0.5594 -0.0974  0.4181  6.3463
##
## Random effects:
##  Groups            Name                   Variance  Std.Dev. Corr
##  child_id:study_id (Intercept)            1.716e+00 1.30991
##                    s.time_centered        4.486e-04 0.02118   0.08
##                    phaseB                 6.796e-01 0.82435  -0.42 -0.41
##                    phaseC                 1.415e+00 1.18968  -0.25  0.49 -0.08
##                    s.time_centered:phaseB 5.539e-04 0.02354  -0.45 -0.69  0.35
##                    s.time_centered:phaseC 3.673e-04 0.01917   0.62 -0.33 -0.13
##  study_id          (Intercept)            2.210e+01 4.70135
##                    s.time_centered        1.024e-03 0.03199  -0.46
##                    phaseB                 1.581e+00 1.25742   0.01 -0.14
##                    phaseC                 3.245e+00 1.80147  -0.04 -0.17 -0.32
##                    s.time_centered:phaseB 4.848e-03 0.06963  -0.04 -0.09  0.38
##                    s.time_centered:phaseC 5.095e-03 0.07138   0.17 -0.29 -0.27
##  Residual                                 9.915e-01 0.99573
##
##
##
##
##
##  -0.04
##  -0.83 -0.35
##
##
##
##
##   0.39
##  -0.41 -0.92
##
## Number of obs: 7381, groups:  child_id:study_id, 270; study_id, 77
##
## Fixed effects:
##                                                        Estimate Std. Error
## (Intercept)                                            4.693390   0.699409
## s.time_centered                                       -0.006893   0.015436
```

```
## phaseB                                          -0.640850   0.349320
## problem_aggressive                               0.507318   0.520271
## phaseC                                          -0.646594   0.659206
## problem_anti_social                              2.047601   1.545115
## problem_noncompliance                            0.410098   0.641575
## problem_oppositional_stubborn                    0.448256   0.603922
## problem_other                                    0.275257   0.762877
## s.time_centered:phaseB                          -0.049124   0.019321
## s.time_centered:problem_aggressive               0.011286   0.016582
## phaseB:problem_aggressive                       -0.829596   0.359854
## s.time_centered:phaseC                           0.056072   0.014389
## problem_aggressive:phaseC                        0.007795   0.712596
## s.time_centered:problem_anti_social              0.004637   0.048853
## phaseB:problem_anti_social                      -1.254652   1.021829
## phaseC:problem_anti_social                      -0.821570   1.482663
## s.time_centered:problem_noncompliance            0.003394   0.018046
## phaseB:problem_noncompliance                    -1.031048   0.422052
## phaseC:problem_noncompliance                     0.093537   0.762744
## s.time_centered:problem_oppositional_stubborn    0.010015   0.017468
## phaseB:problem_oppositional_stubborn            -0.610689   0.413563
## phaseC:problem_oppositional_stubborn             0.339466   0.805235
## s.time_centered:problem_other                   -0.007284   0.022529
## phaseB:problem_other                            -0.696439   0.522773
## phaseC:problem_other                             0.792921   1.316874
## s.time_centered:phaseB:problem_aggressive       -0.001952   0.019632
## s.time_centered:problem_aggressive:phaseC       -0.023954   0.013439
## s.time_centered:phaseB:problem_anti_social       0.012878   0.053434
## s.time_centered:phaseC:problem_anti_social      -0.024834   0.027902
## s.time_centered:phaseB:problem_noncompliance     0.005036   0.022166
## s.time_centered:phaseC:problem_noncompliance    -0.012275   0.015990
## s.time_centered:phaseB:problem_oppositional_stubborn -0.019220   0.020533
## s.time_centered:phaseC:problem_oppositional_stubborn -0.003134   0.013919
## s.time_centered:phaseB:problem_other            -0.008174   0.027791
## s.time_centered:phaseC:problem_other            -0.011824   0.019209
##                                                 t value
## (Intercept)                                       6.711
## s.time_centered                                  -0.447
## phaseB                                           -1.835
## problem_aggressive                                0.975
## phaseC                                           -0.981
## problem_anti_social                               1.325
## problem_noncompliance                             0.639
## problem_oppositional_stubborn                     0.742
## problem_other                                     0.361
## s.time_centered:phaseB                           -2.542
## s.time_centered:problem_aggressive                0.681
## phaseB:problem_aggressive                        -2.305
## s.time_centered:phaseC                            3.897
## problem_aggressive:phaseC                         0.011
## s.time_centered:problem_anti_social               0.095
## phaseB:problem_anti_social                       -1.228
## phaseC:problem_anti_social                       -0.554
## s.time_centered:problem_noncompliance             0.188
## phaseB:problem_noncompliance                     -2.443
```

```
## phaseC:problem_noncompliance                            0.123
## s.time_centered:problem_oppositional_stubborn           0.573
## phaseB:problem_oppositional_stubborn                   -1.477
## phaseC:problem_oppositional_stubborn                    0.422
## s.time_centered:problem_other                          -0.323
## phaseB:problem_other                                   -1.332
## phaseC:problem_other                                    0.602
## s.time_centered:phaseB:problem_aggressive              -0.099
## s.time_centered:problem_aggressive:phaseC              -1.782
## s.time_centered:phaseB:problem_anti_social              0.241
## s.time_centered:phaseC:problem_anti_social             -0.890
## s.time_centered:phaseB:problem_noncompliance            0.227
## s.time_centered:phaseC:problem_noncompliance           -0.768
## s.time_centered:phaseB:problem_oppositional_stubborn  -0.936
## s.time_centered:phaseC:problem_oppositional_stubborn  -0.225
## s.time_centered:phaseB:problem_other                   -0.294
## s.time_centered:phaseC:problem_other                   -0.616
```

```
##
## Correlation matrix not shown by default, as p = 36 > 12.
## Use print(x, correlation=TRUE)  or
##     vcov(x)        if you need it
```

```
## optimizer (bobyqa) convergence code: 0 (OK)
## boundary (singular) fit: see help('isSingular')
```

### Outcomes of the Model With Type of Problem Behavior as Moderator (0 = aggressive)

```
## Linear mixed model fit by REML ['lmerMod']
## Formula:
## outcome_std ~ 1 + s.time_centered * phaseB * problem_tempered_irritable +
##     s.time_centered * phaseC * problem_tempered_irritable + s.time_centered *
##     phaseB * problem_anti_social + s.time_centered * phaseC *
##     problem_anti_social + s.time_centered * phaseB * problem_noncompliance +
##     s.time_centered * phaseC * problem_noncompliance + s.time_centered *
##     phaseB * problem_oppositional_stubborn + s.time_centered *
##     phaseC * problem_oppositional_stubborn + s.time_centered *
##     phaseB * problem_other + s.time_centered * phaseC * problem_other +
##     (1 + s.time_centered + phaseB + s.time_centered * phaseB +
##         phaseC + s.time_centered * phaseC | study_id/child_id)
##    Data: GraphData
## Control: lmerControl(optimizer = "bobyqa", optCtrl = list(maxfun = 7e+05))
##
## REML criterion at convergence: 23404.7
##
## Scaled residuals:
##     Min      1Q  Median      3Q     Max
## -4.0055 -0.5594 -0.0974  0.4181  6.3463
##
## Random effects:
##  Groups            Name                   Variance  Std.Dev. Corr
##  child_id:study_id (Intercept)            1.716e+00 1.30991
```

```
##                    s.time_centered       4.487e-04 0.02118   0.08
##                    phaseB                6.796e-01 0.82436  -0.42 -0.41
##                    phaseC                1.415e+00 1.18965  -0.25  0.49 -0.08
##                    s.time_centered:phaseB 5.539e-04 0.02354  -0.45 -0.69  0.35
##                    s.time_centered:phaseC 3.673e-04 0.01916   0.62 -0.33 -0.13
##  study_id          (Intercept)           2.210e+01 4.70133
##                    s.time_centered       1.024e-03 0.03199  -0.46
##                    phaseB                1.581e+00 1.25739   0.01 -0.14
##                    phaseC                3.245e+00 1.80151  -0.04 -0.17 -0.32
##                    s.time_centered:phaseB 4.848e-03 0.06963  -0.04 -0.09  0.38
##                    s.time_centered:phaseC 5.095e-03 0.07138   0.17 -0.29 -0.27
##  Residual                                9.915e-01 0.99573
##
##
##
##
##
##  -0.04
##  -0.83 -0.35
##
##
##
##
##   0.39
##  -0.41 -0.92
##
## Number of obs: 7381, groups:  child_id:study_id, 270; study_id, 77
##
## Fixed effects:
##                                                   Estimate Std. Error
## (Intercept)                                      5.2007020  0.6048713
## s.time_centered                                  0.0043919  0.0085634
## phaseB                                          -1.4704513  0.2281183
## problem_tempered_irritable                      -0.5073331  0.5202672
## phaseC                                          -0.6387519  0.4002296
## problem_anti_social                              1.5402807  1.4945918
## problem_noncompliance                           -0.0972142  0.5805909
## problem_oppositional_stubborn                   -0.0590707  0.5359709
## problem_other                                   -0.2320717  0.7147459
## s.time_centered:phaseB                          -0.0510746  0.0123465
## s.time_centered:problem_tempered_irritable      -0.0112880  0.0165813
## phaseB:problem_tempered_irritable                0.8296303  0.3598489
## s.time_centered:phaseC                           0.0321172  0.0106443
## problem_tempered_irritable:phaseC               -0.0079014  0.7125862
## s.time_centered:problem_anti_social             -0.0066481  0.0471286
## phaseB:problem_anti_social                      -0.4250620  0.9833798
## phaseC:problem_anti_social                      -0.8293621  1.3800938
## s.time_centered:problem_noncompliance           -0.0078940  0.0132850
## phaseB:problem_noncompliance                    -0.2014405  0.3427665
## phaseC:problem_noncompliance                     0.0855761  0.6261459
## s.time_centered:problem_oppositional_stubborn   -0.0012731  0.0123562
## phaseB:problem_oppositional_stubborn             0.2189432  0.3281745
## phaseC:problem_oppositional_stubborn             0.3314383  0.6029950
## s.time_centered:problem_other                   -0.0185715  0.0186619
```

```
## phaseB:problem_other                                  0.1331903  0.4681461
## phaseC:problem_other                                  0.7852669  1.1783550
## s.time_centered:phaseB:problem_tempered_irritable     0.0019533  0.0196320
## s.time_centered:problem_tempered_irritable:phaseC     0.0239551  0.0134387
## s.time_centered:phaseB:problem_anti_social            0.0148301  0.0512329
## s.time_centered:phaseC:problem_anti_social           -0.0008807  0.0259797
## s.time_centered:phaseB:problem_noncompliance          0.0069892  0.0166111
## s.time_centered:phaseC:problem_noncompliance          0.0116804  0.0127785
## s.time_centered:phaseB:problem_oppositional_stubborn -0.0172672  0.0155882
## s.time_centered:phaseC:problem_oppositional_stubborn  0.0208221  0.0116150
## s.time_centered:phaseB:problem_other                 -0.0062210  0.0240855
## s.time_centered:phaseC:problem_other                  0.0121296  0.0173600
##                                                      t value
## (Intercept)                                            8.598
## s.time_centered                                        0.513
## phaseB                                                -6.446
## problem_tempered_irritable                            -0.975
## phaseC                                                -1.596
## problem_anti_social                                    1.031
## problem_noncompliance                                 -0.167
## problem_oppositional_stubborn                         -0.110
## problem_other                                         -0.325
## s.time_centered:phaseB                                -4.137
## s.time_centered:problem_tempered_irritable            -0.681
## phaseB:problem_tempered_irritable                      2.305
## s.time_centered:phaseC                                 3.017
## problem_tempered_irritable:phaseC                     -0.011
## s.time_centered:problem_anti_social                   -0.141
## phaseB:problem_anti_social                            -0.432
## phaseC:problem_anti_social                            -0.601
## s.time_centered:problem_noncompliance                 -0.594
## phaseB:problem_noncompliance                          -0.588
## phaseC:problem_noncompliance                           0.137
## s.time_centered:problem_oppositional_stubborn         -0.103
## phaseB:problem_oppositional_stubborn                   0.667
## phaseC:problem_oppositional_stubborn                   0.550
## s.time_centered:problem_other                         -0.995
## phaseB:problem_other                                   0.285
## phaseC:problem_other                                   0.666
## s.time_centered:phaseB:problem_tempered_irritable      0.099
## s.time_centered:problem_tempered_irritable:phaseC      1.783
## s.time_centered:phaseB:problem_anti_social             0.289
## s.time_centered:phaseC:problem_anti_social            -0.034
## s.time_centered:phaseB:problem_noncompliance           0.421
## s.time_centered:phaseC:problem_noncompliance           0.914
## s.time_centered:phaseB:problem_oppositional_stubborn  -1.108
## s.time_centered:phaseC:problem_oppositional_stubborn   1.793
## s.time_centered:phaseB:problem_other                  -0.258
## s.time_centered:phaseC:problem_other                   0.699
```

```
##
## Correlation matrix not shown by default, as p = 36 > 12.
## Use print(x, correlation=TRUE)  or
##     vcov(x)        if you need it
```

```
## optimizer (bobyqa) convergence code: 0 (OK)
## boundary (singular) fit: see help('isSingular')
```

### Outcomes of the Model With Type of Problem Behavior as Moderator (0 = anti-social)

```
## Linear mixed model fit by REML ['lmerMod']
## Formula:
## outcome_std ~ 1 + s.time_centered * phaseB * problem_tempered_irritable +
##     s.time_centered * phaseC * problem_tempered_irritable + s.time_centered *
##     phaseB * problem_aggressive + s.time_centered * phaseC *
##     problem_aggressive + s.time_centered * phaseB * problem_noncompliance +
##     s.time_centered * phaseC * problem_noncompliance + s.time_centered *
##     phaseB * problem_oppositional_stubborn + s.time_centered *
##     phaseC * problem_oppositional_stubborn + s.time_centered *
##     phaseB * problem_other + s.time_centered * phaseC * problem_other +
##     (1 + s.time_centered + phaseB + s.time_centered * phaseB +
##         phaseC + s.time_centered * phaseC | study_id/child_id)
##    Data: GraphData
## Control: lmerControl(optimizer = "bobyqa", optCtrl = list(maxfun = 7e+05))
##
## REML criterion at convergence: 23404.7
##
## Scaled residuals:
##     Min      1Q  Median      3Q     Max
## -4.0055 -0.5594 -0.0974  0.4181  6.3463
##
## Random effects:
##  Groups            Name                   Variance  Std.Dev. Corr
##  child_id:study_id (Intercept)            1.716e+00 1.30991
##                    s.time_centered        4.486e-04 0.02118   0.08
##                    phaseB                 6.796e-01 0.82435  -0.42 -0.41
##                    phaseC                 1.415e+00 1.18967  -0.25  0.49 -0.08
##                    s.time_centered:phaseB 5.539e-04 0.02354  -0.45 -0.69  0.35
##                    s.time_centered:phaseC 3.673e-04 0.01917   0.62 -0.33 -0.13
##  study_id          (Intercept)            2.210e+01 4.70131
##                    s.time_centered        1.024e-03 0.03199  -0.46
##                    phaseB                 1.581e+00 1.25742   0.01 -0.14
##                    phaseC                 3.245e+00 1.80150  -0.04 -0.17 -0.32
##                    s.time_centered:phaseB 4.848e-03 0.06963  -0.04 -0.09  0.38
##                    s.time_centered:phaseC 5.095e-03 0.07138   0.17 -0.29 -0.27
##  Residual                                 9.915e-01 0.99573
##
##
##
##
##
##  -0.04
##  -0.83 -0.35
##
##
##
##
```

```
##   0.39
##  -0.41 -0.92
##
## Number of obs: 7381, groups:  child_id:study_id, 270; study_id, 77
##
## Fixed effects:
##                                                       Estimate Std. Error
## (Intercept)                                          6.7410001  1.5268605
## s.time_centered                                     -0.0022558  0.0464291
## phaseB                                              -1.8955089  0.9684886
## problem_tempered_irritable                          -2.0476172  1.5451092
## phaseC                                              -1.4681868  1.3336927
## problem_aggressive                                  -1.5402901  1.4945948
## problem_noncompliance                               -1.6375193  1.5499214
## problem_oppositional_stubborn                       -1.5993568  1.5003931
## problem_other                                       -1.7723557  1.6037877
## s.time_centered:phaseB                              -0.0362456  0.0506781
## s.time_centered:problem_tempered_irritable          -0.0046374  0.0488535
## phaseB:problem_tempered_irritable                    1.2546624  1.0218277
## s.time_centered:phaseC                               0.0312377  0.0259077
## problem_tempered_irritable:phaseC                    0.8215831  1.4826688
## s.time_centered:problem_aggressive                   0.0066492  0.0471290
## phaseB:problem_aggressive                            0.4250594  0.9833921
## phaseC:problem_aggressive                            0.8293856  1.3800993
## s.time_centered:problem_noncompliance               -0.0012430  0.0475725
## phaseB:problem_noncompliance                         0.2236142  1.0068881
## phaseC:problem_noncompliance                         0.9151345  1.4358381
## s.time_centered:problem_oppositional_stubborn        0.0053775  0.0473729
## phaseB:problem_oppositional_stubborn                 0.6439752  0.9920421
## phaseC:problem_oppositional_stubborn                 1.1610553  1.4096193
## s.time_centered:problem_other                       -0.0119214  0.0493785
## phaseB:problem_other                                 0.5582249  1.0571652
## phaseC:problem_other                                 1.6144997  1.7624812
## s.time_centered:phaseB:problem_tempered_irritable   -0.0128783  0.0534344
## s.time_centered:problem_tempered_irritable:phaseC    0.0248340  0.0279020
## s.time_centered:phaseB:problem_aggressive           -0.0148309  0.0512330
## s.time_centered:phaseC:problem_aggressive            0.0008804  0.0259798
## s.time_centered:phaseB:problem_noncompliance        -0.0078427  0.0522217
## s.time_centered:phaseC:problem_noncompliance         0.0125588  0.0276013
## s.time_centered:phaseB:problem_oppositional_stubborn -0.0320986  0.0515675
## s.time_centered:phaseC:problem_oppositional_stubborn  0.0217002  0.0262054
## s.time_centered:phaseB:problem_other                -0.0210519  0.0547767
## s.time_centered:phaseC:problem_other                 0.0130099  0.0294424
##                                                     t value
## (Intercept)                                           4.415
## s.time_centered                                      -0.049
## phaseB                                               -1.957
## problem_tempered_irritable                           -1.325
## phaseC                                               -1.101
## problem_aggressive                                   -1.031
## problem_noncompliance                                -1.057
## problem_oppositional_stubborn                        -1.066
## problem_other                                        -1.105
## s.time_centered:phaseB                               -0.715
```

```
## s.time_centered:problem_tempered_irritable           -0.095
## phaseB:problem_tempered_irritable                     1.228
## s.time_centered:phaseC                                1.206
## problem_tempered_irritable:phaseC                     0.554
## s.time_centered:problem_aggressive                    0.141
## phaseB:problem_aggressive                             0.432
## phaseC:problem_aggressive                             0.601
## s.time_centered:problem_noncompliance                -0.026
## phaseB:problem_noncompliance                          0.222
## phaseC:problem_noncompliance                          0.637
## s.time_centered:problem_oppositional_stubborn         0.114
## phaseB:problem_oppositional_stubborn                  0.649
## phaseC:problem_oppositional_stubborn                  0.824
## s.time_centered:problem_other                        -0.241
## phaseB:problem_other                                  0.528
## phaseC:problem_other                                  0.916
## s.time_centered:phaseB:problem_tempered_irritable    -0.241
## s.time_centered:problem_tempered_irritable:phaseC     0.890
## s.time_centered:phaseB:problem_aggressive            -0.289
## s.time_centered:phaseC:problem_aggressive             0.034
## s.time_centered:phaseB:problem_noncompliance         -0.150
## s.time_centered:phaseC:problem_noncompliance          0.455
## s.time_centered:phaseB:problem_oppositional_stubborn  -0.622
## s.time_centered:phaseC:problem_oppositional_stubborn   0.828
## s.time_centered:phaseB:problem_other                 -0.384
## s.time_centered:phaseC:problem_other                  0.442

##
## Correlation matrix not shown by default, as p = 36 > 12.
## Use print(x, correlation=TRUE)  or
##     vcov(x)        if you need it

## optimizer (bobyqa) convergence code: 0 (OK)
## boundary (singular) fit: see help('isSingular')
```

## Outcomes of the Model With Type of Problem Behavior as Moderator (0 = non-compliance)

```
## Linear mixed model fit by REML ['lmerMod']
## Formula:
## outcome_std ~ 1 + s.time_centered * phaseB * problem_tempered_irritable +
##     s.time_centered * phaseC * problem_tempered_irritable + s.time_centered *
##     phaseB * problem_aggressive + s.time_centered * phaseC *
##     problem_aggressive + s.time_centered * phaseB * problem_anti_social +
##     s.time_centered * phaseC * problem_anti_social + s.time_centered *
##     phaseB * problem_oppositional_stubborn + s.time_centered *
##     phaseC * problem_oppositional_stubborn + s.time_centered *
##     phaseB * problem_other + s.time_centered * phaseC * problem_other +
##     (1 + s.time_centered + phaseB + s.time_centered * phaseB +
##         phaseC + s.time_centered * phaseC | study_id/child_id)
##    Data: GraphData
## Control: lmerControl(optimizer = "bobyqa", optCtrl = list(maxfun = 5e+05))
##
```

```
## REML criterion at convergence: 23404.7
##
## Scaled residuals:
##     Min      1Q  Median      3Q     Max
## -4.0055 -0.5594 -0.0974  0.4181  6.3463
##
## Random effects:
##  Groups            Name                   Variance  Std.Dev. Corr
##  child_id:study_id (Intercept)            1.716e+00 1.30991
##                    s.time_centered        4.486e-04 0.02118   0.08
##                    phaseB                 6.796e-01 0.82435  -0.42 -0.41
##                    phaseC                 1.415e+00 1.18968  -0.25  0.49 -0.08
##                    s.time_centered:phaseB 5.539e-04 0.02354  -0.45 -0.69  0.35
##                    s.time_centered:phaseC 3.673e-04 0.01917   0.62 -0.33 -0.13
##  study_id          (Intercept)            2.210e+01 4.70132
##                    s.time_centered        1.024e-03 0.03199  -0.46
##                    phaseB                 1.581e+00 1.25741   0.01 -0.14
##                    phaseC                 3.245e+00 1.80148  -0.04 -0.17 -0.32
##                    s.time_centered:phaseB 4.848e-03 0.06963  -0.04 -0.09  0.38
##                    s.time_centered:phaseC 5.095e-03 0.07138   0.17 -0.29 -0.27
##  Residual                                 9.915e-01 0.99573
##
##
##
##
##
##  -0.04
##  -0.83 -0.35
##
##
##
##
##   0.39
##  -0.41 -0.92
##
## Number of obs: 7381, groups:  child_id:study_id, 270; study_id, 77
##
## Fixed effects:
##                                                   Estimate Std. Error
## (Intercept)                                      5.1034770  0.6891284
## s.time_centered                                 -0.0035018  0.0106708
## phaseB                                          -1.6718908  0.2982486
## problem_tempered_irritable                      -0.4101189  0.6415713
## phaseC                                          -0.5531400  0.5484967
## problem_aggressive                               0.0972337  0.5805904
## problem_anti_social                              1.6375386  1.5499172
## problem_oppositional_stubborn                    0.0381389  0.5961230
## problem_other                                   -0.1348475  0.7723847
## s.time_centered:phaseB                          -0.0440854  0.0152200
## s.time_centered:problem_tempered_irritable      -0.0033942  0.0180456
## phaseB:problem_tempered_irritable                1.0310725  0.4220464
## s.time_centered:phaseC                           0.0437971  0.0131525
## problem_tempered_irritable:phaseC               -0.0934878  0.7627362
## s.time_centered:problem_aggressive               0.0078938  0.0132851
```

```
## phaseB:problem_aggressive                              0.2014445  0.3427686
## phaseC:problem_aggressive                             -0.0856175  0.6261478
## s.time_centered:problem_anti_social                    0.0012451  0.0475721
## phaseB:problem_anti_social                            -0.2236147  1.0068780
## phaseC:problem_anti_social                            -0.9149523  1.4358284
## s.time_centered:problem_oppositional_stubborn          0.0066208  0.0138643
## phaseB:problem_oppositional_stubborn                   0.4203754  0.3704533
## phaseC:problem_oppositional_stubborn                   0.2458919  0.6793231
## s.time_centered:problem_other                         -0.0106770  0.0196493
## phaseB:problem_other                                   0.3346105  0.5033474
## phaseC:problem_other                                   0.6996308  1.2690354
## s.time_centered:phaseB:problem_tempered_irritable     -0.0050358  0.0221657
## s.time_centered:problem_tempered_irritable:phaseC      0.0122748  0.0159899
## s.time_centered:phaseB:problem_aggressive             -0.0069893  0.0166111
## s.time_centered:phaseC:problem_aggressive             -0.0116798  0.0127785
## s.time_centered:phaseB:problem_anti_social             0.0078411  0.0522216
## s.time_centered:phaseC:problem_anti_social            -0.0125606  0.0276012
## s.time_centered:phaseB:problem_oppositional_stubborn -0.0242562  0.0181125
## s.time_centered:phaseC:problem_oppositional_stubborn  0.0091415  0.0140549
## s.time_centered:phaseB:problem_other                  -0.0132107  0.0257405
## s.time_centered:phaseC:problem_other                   0.0004496  0.0192671
##                                                      t value
## (Intercept)                                            7.406
## s.time_centered                                       -0.328
## phaseB                                                -5.606
## problem_tempered_irritable                            -0.639
## phaseC                                                -1.008
## problem_aggressive                                     0.167
## problem_anti_social                                    1.057
## problem_oppositional_stubborn                          0.064
## problem_other                                         -0.175
## s.time_centered:phaseB                                -2.897
## s.time_centered:problem_tempered_irritable            -0.188
## phaseB:problem_tempered_irritable                      2.443
## s.time_centered:phaseC                                 3.330
## problem_tempered_irritable:phaseC                     -0.123
## s.time_centered:problem_aggressive                     0.594
## phaseB:problem_aggressive                              0.588
## phaseC:problem_aggressive                             -0.137
## s.time_centered:problem_anti_social                    0.026
## phaseB:problem_anti_social                            -0.222
## phaseC:problem_anti_social                            -0.637
## s.time_centered:problem_oppositional_stubborn          0.478
## phaseB:problem_oppositional_stubborn                   1.135
## phaseC:problem_oppositional_stubborn                   0.362
## s.time_centered:problem_other                         -0.543
## phaseB:problem_other                                   0.665
## phaseC:problem_other                                   0.551
## s.time_centered:phaseB:problem_tempered_irritable     -0.227
## s.time_centered:problem_tempered_irritable:phaseC      0.768
## s.time_centered:phaseB:problem_aggressive             -0.421
## s.time_centered:phaseC:problem_aggressive             -0.914
## s.time_centered:phaseB:problem_anti_social             0.150
## s.time_centered:phaseC:problem_anti_social            -0.455
```

```
## s.time_centered:phaseB:problem_oppositional_stubborn  -1.339
## s.time_centered:phaseC:problem_oppositional_stubborn   0.650
## s.time_centered:phaseB:problem_other                  -0.513
## s.time_centered:phaseC:problem_other                   0.023

##
## Correlation matrix not shown by default, as p = 36 > 12.
## Use print(x, correlation=TRUE)  or
##     vcov(x)        if you need it

## optimizer (bobyqa) convergence code: 0 (OK)
## boundary (singular) fit: see help('isSingular')
```

### Outcomes of the Model With Type of Problem Behavior as Moderator (0 = stubborn)

```
## Linear mixed model fit by REML ['lmerMod']
## Formula:
## outcome_std ~ 1 + s.time_centered * phaseB * problem_tempered_irritable +
##     s.time_centered * phaseC * problem_tempered_irritable + s.time_centered *
##     phaseB * problem_aggressive + s.time_centered * phaseC *
##     problem_aggressive + s.time_centered * phaseB * problem_anti_social +
##     s.time_centered * phaseC * problem_anti_social + s.time_centered *
##     phaseB * problem_noncompliance + s.time_centered * phaseC *
##     problem_noncompliance + s.time_centered * phaseB * problem_other +
##     s.time_centered * phaseC * problem_other + (1 + s.time_centered +
##     phaseB + s.time_centered * phaseB + phaseC + s.time_centered *
##     phaseC | study_id/child_id)
##    Data: GraphData
## Control: lmerControl(optimizer = "bobyqa", optCtrl = list(maxfun = 5e+05))
##
## REML criterion at convergence: 23404.7
##
## Scaled residuals:
##     Min      1Q  Median      3Q     Max
## -4.0055 -0.5594 -0.0974  0.4181  6.3463
##
## Random effects:
##  Groups            Name                   Variance  Std.Dev. Corr
##  child_id:study_id (Intercept)            1.716e+00 1.30991
##                    s.time_centered        4.486e-04 0.02118   0.08
##                    phaseB                 6.796e-01 0.82436  -0.42 -0.41
##                    phaseC                 1.415e+00 1.18968  -0.25  0.49 -0.08
##                    s.time_centered:phaseB 5.539e-04 0.02354  -0.45 -0.69  0.35
##                    s.time_centered:phaseC 3.673e-04 0.01917   0.62 -0.33 -0.13
##  study_id          (Intercept)            2.210e+01 4.70135
##                    s.time_centered        1.024e-03 0.03199  -0.46
##                    phaseB                 1.581e+00 1.25740   0.01 -0.14
##                    phaseC                 3.245e+00 1.80148  -0.04 -0.17 -0.32
##                    s.time_centered:phaseB 4.848e-03 0.06963  -0.04 -0.09  0.38
##                    s.time_centered:phaseC 5.095e-03 0.07138   0.17 -0.29 -0.27
##  Residual                                 9.915e-01 0.99573
##
```

```
##
##
##
##
##  -0.04
##  -0.83 -0.35
##
##
##
##
##   0.39
##  -0.41 -0.92
##
## Number of obs: 7381, groups:  child_id:study_id, 270; study_id, 77
##
## Fixed effects:
##                                                    Estimate Std. Error t value
## (Intercept)                                        5.141634   0.650864   7.900
## s.time_centered                                    0.003121   0.010075   0.310
## phaseB                                            -1.251531   0.277801  -4.505
## problem_tempered_irritable                        -0.448249   0.603921  -0.742
## phaseC                                            -0.307108   0.512551  -0.599
## problem_aggressive                                 0.059083   0.535974   0.110
## problem_anti_social                                1.599377   1.500397   1.066
## problem_noncompliance                             -0.038146   0.596127  -0.064
## problem_other                                     -0.173006   0.709311  -0.244
## s.time_centered:phaseB                            -0.068344   0.014161  -4.826
## s.time_centered:problem_tempered_irritable        -0.010014   0.017468  -0.573
## phaseB:problem_tempered_irritable                  0.610682   0.413561   1.477
## s.time_centered:phaseC                             0.052938   0.011750   4.505
## problem_tempered_irritable:phaseC                 -0.339473   0.805237  -0.422
## s.time_centered:problem_aggressive                 0.001272   0.012357   0.103
## phaseB:problem_aggressive                         -0.218925   0.328179  -0.667
## phaseC:problem_aggressive                         -0.331678   0.603009  -0.550
## s.time_centered:problem_anti_social               -0.005377   0.047373  -0.114
## phaseB:problem_anti_social                        -0.643990   0.992039  -0.649
## phaseC:problem_anti_social                        -1.161020   1.409617  -0.824
## s.time_centered:problem_noncompliance             -0.006620   0.013865  -0.477
## phaseB:problem_noncompliance                      -0.420368   0.370457  -1.135
## phaseC:problem_noncompliance                      -0.245948   0.679335  -0.362
## s.time_centered:problem_other                     -0.017299   0.018925  -0.914
## phaseB:problem_other                              -0.085731   0.482607  -0.178
## phaseC:problem_other                               0.453413   1.249130   0.363
## s.time_centered:phaseB:problem_tempered_irritable  0.019220   0.020533   0.936
## s.time_centered:problem_tempered_irritable:phaseC  0.003134   0.013919   0.225
## s.time_centered:phaseB:problem_aggressive          0.017267   0.015588   1.108
## s.time_centered:phaseC:problem_aggressive         -0.020820   0.011615  -1.792
## s.time_centered:phaseB:problem_anti_social         0.032098   0.051568   0.622
## s.time_centered:phaseC:problem_anti_social        -0.021700   0.026205  -0.828
## s.time_centered:phaseB:problem_noncompliance       0.024255   0.018113   1.339
## s.time_centered:phaseC:problem_noncompliance      -0.009141   0.014055  -0.650
## s.time_centered:phaseB:problem_other               0.011046   0.023350   0.473
## s.time_centered:phaseC:problem_other              -0.008690   0.015399  -0.564
```

```
##
## Correlation matrix not shown by default, as p = 36 > 12.
## Use print(x, correlation=TRUE)  or
##     vcov(x)        if you need it

## optimizer (bobyqa) convergence code: 0 (OK)
## boundary (singular) fit: see help('isSingular')
```
