# Supplementary material for: A Multilevel Meta-Analysis of Single-Case Research on Interventions for Externalizing Behavior Problems in Children and Adolescents
Source: JAACAP Open. 2025 Dec 18;4(2):220–31. doi: 10.1016/j.jaacop.2025.12.002 (PMC13043497; doi:10.1016/j.jaacop.2025.12.002)
Supplement: Supplemental Data [file mmc6.pdf]

Supplement #2 – Overview of included and excluded studies

No.	Gr. Lit.	Title	Author	Year	Reason for exclusion
S0001		The effects of social skills training on the socially appropriate and antisocial behaviors of elementary students with serious emotional disorders and at-risk behaviors in the general education setting.	Adedapo	1999	99
S0002		The Effect of Behavioral Management and Social Skills Training Program on Behavioral and Adaptive Problems of Male Adolescents with High -Functioning Autism	Akrami	2019	2
S0003		The use of an enhanced simplified habit-reversal procedure to reduce disruptive outbursts during athletic performance.	Allen	1998	2
S0004		Training parents via telehealth to teach manding to children with ASD to replace problem behavior.	Almulhim	2023	2
S0005		ACCEPTANCE AND COMMITMENT THERAPY (ACT) IN A CHILD WITH DYSFUNCTIONAL JEALOUSY AND RUMINATION	Alonso	2022	0
S0006		Effects of Joint Action Routine on Improving Social Behavior of a Student with ASD	Alwahbi	2023	99
S0007		Externalizing behavioral symptoms in adjudicated youth and solution-focused brief therapy delivered through video conferencing.	Anderson	2019	0
S0009		Using narrated literacy-based behavioural interventions to decrease episodes of physical aggression in elementary students with disabilities.	Anderson	2016	2
S0010		Effects of Acceptance and Commitment Training Plus Behavior Parent Training on Parental Implementation of Autism Treatment	Andrews	2022	2
S0011		Using modified TAGteachTM procedures in increasing skill acquisition of dance movements for a child with multiple diagnoses.	Arnall	2019	2
S0012		Evaluation of short and medium-term effectiveness of Early Intervention Program for Child-to-Parent Violence	Arnoso	2021	4
S0013		Combining acceptance and commitment therapy with parent-child interaction therapy when working with a child with serious behavioral problems.	Ascanio	2018	0
S0014		Classroom Management in Self-Contained Classrooms for Children with Autism: Extending Research on the Color Wheel System	Aspiranti	2019	3
S0015	Y	Incorporating a Class-Wide Behavioral System to Decrease Disruptive Behaviors in the Inclusive Classroom	Aspiranti	2018	2
S0016		Video self-modeling: A promising strategy for noncompliant children.	Axelrod	2014	0
S0017		Adapting parent-child interaction therapy to train Head Start teachers in behavior management.	Bahl	2001	0
S0018		Feasibility and potential efficacy of the family-centered Prevent-Teach-Reinforce model with families of children with developmental disorders.	Bailey	2015	2

S0019		A case study integrating CBT with narrative therapy externalizing techniques with a child with OCD: How to flush away the Silly Gremlin. A single-case experimental design.	Banting	2017	1
S0020		Supporting teachers to use restorative practice circles to improve student outcomes in elementary school classrooms.	Barrasso	2020	99
S0021		A family in crisis: Replacing the aggressive behavior of a child with autism toward an infant sibling.	Barry	2001	1
S0022		A Practical Application of Self-Management for Students Diagnosed with Attention-Deficit/Hyperactivity Disorder.	Barry	2003	2
S0024		A multiple baseline investigation of conjoint behavioral consultation (CBC) facilitated by a pediatric mental health consultant.	Bellinger	2012	0
S0025		Effectiveness of deep brain stimulation in refractory and drug-resistant aggressiveness in autism spectrum disorder	Benedetti	2023	5
S0026		Using social stories to decrease aggression and increase positive peer interactions in normally developing pre-school children.	Benish	2011	0
S0027		Effect of a psychoeducation treatment model on adolescent symptom reduction.	Beyer	2015	99
S0028		Teleconsultation: The use of technology to improve evidence-based practices in rural communities	Bice	2016	0
S0029		The effectiveness of solution-focused brief therapy with incarcerated youth.	Black	2021	99
S0030		Positive behavior support through family-school collaboration for young children with autism.	Blair	2011	2
S0032		The effects of teacher-directed and peer-directed social skills training on the social interaction skills of middle school students with serious emotional disturbances.	Blake	1999	99
S0033		Stimulus control over family problem-solving behavior: The Family Contract Game.	Blechman	1976	1
S0034		Improving parent-child interactions and generalized problem-solving skills in families of children with attention-deficit/hyperactivity disorder through adapted parent child interaction therapy.	Bobal	2020	2
S0035		An evaluation of the caught being good game with an adolescent student population.	Bohan	2021	3
S0036	Y	Videotape feedback as a behavior management technique	Booth	1983	2
S0037		Social skills training for highly aggressive children: Treatment in an inpatient psychiatric setting.	Bornstein	1980	0
S0038		Parent-Child Interaction Therapy With a Spanish-Speaking Family.	Borrego	2006	12
S0040		OVER-CHASTISEMENT, CHILD NONCOMPLIANCE AND PARENTING SKILLS - A BEHAVIORAL INTERVENTION BY A FAMILY CENTER SOCIAL-WORKER	Bourn	1993	0
S0041		Parent-child interaction therapy with in-room coaching: Results of a preliminary German case study.	Briegel	2015	99
S0042		From the editor.	Bruder	2015	99

S0043		Evaluating technology-based self-monitoring as a Tier 2 intervention across middle school settings.	Bruhn	2017	1
S0044		An analysis of multiple misplaced parental social contingencies.	Budd	1976	2
S0046		A modified application of a training program for socially insecure children from socioculturally disadvantaged populations: A single-case study with children from a day-treatment group.	Burk	1991	99
S0047		An evaluation of deferred time-out to treat noncompliance in the classroom setting.	Buzenski	2018	0
S0048		Culturally adapted social, emotional, and behavioral support for Black male learners.	Campbell	2023	0
S0049		Social-Emotional and Behavioral Support for First- and Second-Grade Black Learners at Risk for Emotional and Behavioral Problems	Campbell	2023	4
S0050		Adding function-based behavioral support to first step to success: Integrating individualized and manualized practices.	Carter	2009	0
S0051	Y	Adding functional behavioral assessment to first step to sucess: A case study	Carter	2007	0
S0053		Effects of kinder training on preschool children's externalizing behavior: A single-case design.	Chen	2021	1
S0054		Reducing perseverative requesting and other problem behavior in a young girl with autism: a sequentially implemented intervention package	Chen	2023	99
S0055		Feasibility of low-intensity psychological interventions for emotional and behavioural difficulties in children and young people with genetic conditions: A case series.	Ching	2022	3
S0057		Using a multicomponent function-based intervention to support students with attention deficit hyperactivity disorder.	Cho	2017	2
S0062		Solution-focused family therapy with three aggressive and oppositional-acting children: An N=1 empirical study.	Conoley	2003	0
S0063		Preliminary Study of the Confined, Collateral, and Combined Effects of Reading and Behavioral Interventions: Evidence for a Transactional Relationship	Cook	2012	2
S0064		The effects of martial arts on inattention, impulsivity, hyperactivity and aggression in children with attention-deficit/hyperactivity disorder: A single-subject multiple-baseline design across participants.	Cooper	2005	99
S0065		Emotional Dysregulation in Preschoolers with Autism Spectrum Disorder-A Sample of Romanian Children.	Costescu	2021	1
S0067		The effects of a technology delivered self-monitoring system and video self modeling on the disruptive behavior of adolescents with autism.	Crutchfield	2015	2
S0068		Evaluation of the integrated therapy model in preschool education for children with autism spectrum disorder in China.	Cui	2023	2
S0069		Aggression Replacement Training in Australia: Youth Justice Pilot Study	Currie	2009	2
S0070		Altering the function of commands presented to boys with oppositional and hyperactive behavior.	Danforth	2001	0

S0071		The outcome of parent training using the Behavior Management Flow Chart with mothers and their children with oppositional defiant disorder and attention-deficit hyperactivity disorder.	Danforth	1998	0
S0072		Anger control training for adolescents in residential treatment.	Dangel	1989	12
S0073		The classroom password: A class-wide intervention to increase academic engagement.	Dart	2016	3
S0075	Y	The impact of self-modeling on problem behaviors in school-age children	Davis	1979	0
S0076		Evaluating the Prevent-Teach-Reinforce Model for High School Students With Autism Spectrum Disorder	Deenihan	2023	2
S0078		The effectiveness of structural family therapy in repairing behavioral problems and improving family functioning in single-parent families in iran.	Dehghani	2022	2
S0080		The efficacy of problem-solving consultation for homeschooled students with behavior concerns.	DeRish	2020	1
S0081		The efficacy of problem-solving consultation and evidence-based interventions for homeschooled students with learning-related behavior concerns.	DeRoos	2019	99
S0082		Teacher child interaction training (TCIT) creates a positive classroom environment: Improving attachment and management of child behavior problems.	Devers	2015	2
S0083		A playground social skills intervention for children exhibiting emotional and behavioral problems.	DiFolco	2014	99
S0084	Y	A component analysis of self-management training with behaviorally disordered youth	DiGangi	1992	0
S0085		Effects of child-centered play therapy (CCPT) on disruptive behavior of at-risk preschool children in Head Start.	Dillman	2021	4
S0008		Training and generalization of social skills with problem children.	Anderson	1987	99
S0086		Investigating group Adlerian play therapy for children with disruptive behaviors: A single-case research design.	Dillman	2019	2
S0087		The effects of Child Teacher Relationship Training (CTRT) on residential care workers: A mixed methods study.	Donald	2016	2
S0088		Evaluating a DBR self-monitoring intervention for middle schoolers with ADHD.	Donham	2023	2
S0090		Success-based, noncoercive treatment of oppositional behavior in children from violent homes.	Ducharme	2000	3
S0091	Y	Functional communication training to reduce challenging behavior	Durand	1991	2
S0092		The impact of a caregiver-training program on caregiver's generalization ability and child's compliance.	Ebanks	2007	99
S0093		Feasibility and acceptability of a compressed caregiver training program to treat child behavior problems.	Edelstein	2023	2
S0094		Adolescent psychiatric patients: Modifying aggressive behavior with social skills training.	Elder	1979	3

S0096		Secondary prevention efforts at a residential facility for students with emotional and behavioral disorders: Function-based check-in, check-out.	Ennis	2012	0
S0100		Effects of the Promove-Pais program, a behavioral psychotherapy applied to caregivers of adolescents with behavioral problem.	Ferreira	2017	3
S0101		Integrating the acceptance and commitment therapy with the parent-child interaction therapy in an oppositional defiant child	Ferro	2017	0
S0023		Evaluating the effects of "On-Task in a Box" as a class-wide intervention for increasing on-task behavior and academic performance.	Battaglia	2017	1
S0102		Using multiple schedules during functional communication training to promote rapid transfer of treatment effects.	Fisher	2015	1
S0104		Efficacy of a no-team version of the good behavior game in high school classrooms.	Ford	2020	3
S0105		Using Parent-Child Interaction Therapy with an adolescent diagnosed with Asperger's syndrome.	Ford	2013	99
S0106		The effectiveness of solution-focused therapy with children in a school setting.	Franklin	2001	2
S0031		Use of positive behavior support to address the challenging behavior of young children within a community early childhood program.	Blair	2010	2
S0107		The utilization and effects of positive behavior support strategies on an urban school playground.	Franzen	2008	3
S0108		Parent-child interaction therapy for Japanese working mother and child with behavioral problems: A single case study.	Furuzawa	2020	0
S0109		The effect of a self-monitored relaxation breathing exercise on male adolescent aggressive behavior.	Gaines	2008	2
S0111	Y	Positive interaction between mothers and conduct-problem children: is there training for harmony as well as fighting?.	Gardner	1987	3
S0113		Academic and Behavior Combined Support: Evaluation of an integrated supplemental intervention for early elementary students.	Gettinger	2021	0
S0114		An evaluation of operant behavioural economics in functional communication training for severe problem behaviour.	Gilroy	2019	2
S0039		Efficacy of a Virtual Reality Biofeedback Game (DEEP) to Reduce Anxiety and Disruptive Classroom Behavior: Single-Case Study.	Bossenbroek	2020	2
S0115		Short-term, home-based intervention for child noncompliance using behavioral consultation and a self-help manual.	Gmeinder	1998	1
S0116	Y	A case study of parent–child interaction therapy: Flexible client-centered adaptation of an EST	Gordon	2016	0
S0118		The use of functional assessment and self-management with a first grader.	Grandy	1997	2
S0119		The lunchroom behavior game: A single-case design conceptual replication.	Grasley	2022	2
S0045		Home-based treatment of severe disruptive behaviors: A reinforcement package for preschool and kindergarten children.	Budd	1981	2

S0120		Home-based consultation for parents of young children with behavioral problems	Greene	1999	0
S0122		Regulation of the aggressiveness among pre-school children by the training of mothers and teachers.	Guerra	2011	0
S0052		The effects of errorless compliance training on children in home and school settings.	Cavell	2018	1
S0123		Training parents to create and implement Social StoriesTM: Promoting social competence in children without disabilities.	Gullon	2019	0
S0124		Exploring the efficacy of self-regulatory training as a possible alternative to social skills training.	Gumpel	2000	0
S0125		A case study of the effects of altering instructional interactions on the disruptive behavior of a child identified with severe behavior disorders.	Gunter	1994	0
S0056		Effects of rules and feedback on classroom behavior of adolescents in a residential treatment setting.	Chinnappan	2020	3
S0126		Play Therapy As Effective Options for School-Age Children With Emotional and Behavioral Problems: A Case Series.	Gupta	2023	3
S0058		The Effectiveness of Lurasidone Add-On for Residual Aggressive Behavior and Obsessive Symptoms in Antipsychotic-Treated Children and Adolescents with Tourette Syndrome: Preliminary Evidence from a Case Series.	Colizzi	2021	2
S0059		Evaluating implementation supports delivered within problem-solving consultation.	Collier	2019	3
S0060		Using the Performance Diagnostic Checklist to evaluate and promote paraeducators' treatment fidelity.	Collier	2021	1
S0061		Using active supervision and precorrection to improve transition behaviors in an elementary school.	Colvin	1997	3
S0127		Cognitive-behavioral intervention with the aggressor child in a bullying case	Guzm	2015	99
S0128		Trauma-informed check-in/check-out adaptations for youth with emotional and behavioral disorders and histories of trauma exposures.	Hackney	2024	1
S0129		Impact of MindUP on elementary school students' classroom behaviors: A single-case design pilot study.	Hai	2021	0
S0066		Parent training on generalized use of behavior analytic strategies for decreasing the problem behavior of children with autism spectrum disorder: A data-based case study.	Crone	2016	2
S0130		Cognitive-behavioral training of problem-solving and impulse-control with delinquent adolescents.	Hains	1988	1
S0131		Video self-modeling as an intervention to address noncompliance in preschoolers.	Halberg	2019	99
S0132		Parent-implemented positive behavior support strategies for young children on the autism spectrum: A pilot investigation.	Hampton	2022	2
S0134		Analysis of multiple manding topographies during functional communication training.	Harding	2009	0
S0074		School-Based Interventions Targeting Challenging Behavior of Adolescents with Developmental Disabilities: A Meta-Analysis	David	2023	4

S0135		Using a modified social story intervention in the home environment for children with autism spectrum disorder and its impact on family quality of life.	Harkema	2018	99
S0077		Sertraline as a treatment option for temper outbursts in Prader-Willi syndrome.	Deest	2021	2
S0136		Remote Delivery of Prevent Teach Reinforce for Families	Hodges	2022	0
S0079		Treating family conflicts and behavioral problems in children with attention deficit hyperactivity disorder using parent-child interaction training and structural family therapy, single-case experimental design.	Depry	2008	99
S0137		The use of collaborative problem solving to address challenging behavior among hospitalized children with complex trauma: A case series.	Holmes	2014	2
S0138		An investigation of the HOT DOCS guide for weekly early intervention sessions: A multiple baseline design.	Holyfield	2022	0
S0139		Treating children with early-onset conduct problems and callous-unemotional traits: An empirical evaluation of KooLKIDS.	Houghton	2017	1
S0140		Using functional communication training to alleviate problem behaviors in young children.	Hunt	1999	99
S0141		The social validity of Social StoriesTM for supporting the behavioural and communicative functioning of children with autism spectrum disorder.	Hutchins	2013	2
S0142		Evaluation report of early intervention program in situation of child-to-parent abuse: Parents and children as participants.	Ibabe	2023	3
S0143		A preview of the AIM practice guidance for harmful sexual behaviour between siblings illustrated by a case series	Ibrahim	2023	3
S0144		An initial case series of intensive cognitive-behavioral therapy for obsessive-compulsive disorder in adolescents with autism spectrum disorder.	Iniesta	2018	1
S0089		The daily report card: A simplified and flexible package for classroom behavior management.	Dougherty	1977	2
S0145		An evaluation of the components of a Social StoriesTM intervention package.	Iskander	2013	2
S0146		The effects of parent-child interaction therapy on problem behaviors in three children with autistic disorder.	Jamison	2008	99
S0147		Parent-child interaction therapy as a treatment for ADHD in early childhood: A multiple baseline single-case design.	Jeffries	2016	0
S0148		An experimental analysis of the effects of therapeutic horseback riding on the behavior of children with autism	Jenkins	2013	2
S0150		The good behavior game: A systematic replication in two unruly transitional classrooms.	Johnson	1978	3
S0095		Evaluation report of Early Intervention Program in Situations of Youth-to-Parent Aggression: What happens one year after?	Elgorriaga	2023	3
S0097		Universal TCIT improves teacher-child interactions and management of child behavior.	Fawley	2020	3

S0098	Y	Effectiveness, Acceptability, and Feasibility of the Soles of the Feet Mindfulness-Based Intervention with Elementary School Students	Felver	2013	2
S0099		A case series: Cognitive-behavioral treatment (exposure, relaxation, and rescripting therapy) of trauma-related nightmares experienced by children.	Fernandez	2013	1
S0151		Case Report. Improved Mood and Behavior During Treatment with a Mineral-Vitamin Supplement: An Open-Label Case Series of Children.	Kaplan	2004	3
S0152		Micronutrient treatment for children with emotional and behavioral dysregulation: a case series.	Kaplan	2015	5
S0103		The Caterpillar Game: A SW-PBIS aligned classroom management system.	Floress	2017	3
S0153		Check in-check out intervention for supporting pupils' behaviour: Effectiveness and feasibility in Finnish schools.	Karhu	2019	0
S0154		Implementation of the inclusive CICO Plus intervention for pupils at risk of severe behaviour problems in SWPBS schools.	Karhu	2021	0
S0155		Helping students C.O.P.E.: Effects of a mental health program on adolescents with challenging behaviors.	Katic	2023	0
S0156	Y	Effectiveness of self-observation with behavior disordered elementary school children	Kehle	1986	0
S0157		The effects of role-playing and self-monitoring on the generalization of vocational social skills by behaviorally disordered adolescents.	Kelly	1983	2
S0110		Intervention in bullying and cyberbullying: Assessment of Martin's case	Garaigordobil	2017	2
S0159	Y	Improving the peer interactions of students with emotional and behavioral disorders through self-evaluation procedures: A component analysis and group application.	Kern	1995	0
S0112		Child-centered play therapy and child development: A single-case analysis.	Garofano	2010	2
S0160		Use of a classwide self-management program to improve the behavior of students with emotional and behavioral disorders.	Kern	1994	3
S0162	Y	Effects of a videotape feedback package on the peer interactions of children with serious behavioral and emotional challenges	Kern-Dunlap	1986	2
S0117		The development of an enhanced school home note intervention: Applying key behavioral parenting training components to improve the outcomes of school based behavioral intervention.	Grady	2014	2
S0163		Function-based modification of Check-In/Check-Out to influence escape-maintained behavior.	Kilgus	2016	1
S0165	Y	Adapting parent–child interaction therapy to treat severe conduct problems with callous-unemotional traits: A case study	Kimonis	2012	0
S0121		Modifying the Classroom Environment to Increase Engagement and Decrease Disruption with Students Who Are Deaf or Hard of Hearing	Guardino	2012	2
S0166		Metadata Correction: The Effects of a Virtual Reality-Based Training Program for Adolescents With Disruptive Behavior Problems on Cognitive Distortions and	Klein	2022	4

		Treatment Motivation: Protocol for a Multiple Baseline Single-Case Experimental Design.			
S0167		Parent-child interaction therapy for children with autism spectrum disorder: An analysis of behavioral patterns and treatment barriers.	Knap	2018	0
S0168		A cognitive-behavioral treatment for academic survival skills in disruptive classrooms: A multiple baseline design.	Knapp	1981	99
S0169		Reducing aggression in children with autism toward infant or toddler siblings.	Koegel	1998	2
S0170		Effects of a social story intervention with a modified perspective sentence on preschool-age children with autism.	Krasch	2015	0
S0172		The effects of stability ball seating on the behavior of children with autism during instructional activities.	Krombach	2020	2
S0133		Evaluation of the preschool life skills program in Head Start classrooms: A systematic replication.	Hanley	2014	3
S0173		A single-case study of a self-monitoring intervention for high school students.	Kumm	2020	0
S0174		Maintenance and generalization of preschool teachers' use of behavior specific praise following in situ training.	LaBrot	2018	4
S0175		An evaluation of balance delivered via telehealth.	LaCroix	2023	99
S0176		Transition states in single-case experimental designs: A retrospective consecutive-controlled case series investigation.	Laureano	2023	1
S0177	Y	Effectiveness of home treatment in children and adolescents with externalizing psychiatric disorders	Lay	2001	2
S0178		A compliance matrix to increase teacher praise and improve student behaviors: Incorporating ecological observation data to inform an intervention.	Lazo	2005	99
S0179		Mystery motivator versus reward menu: An investigation of the effects of home-based reinforcement delivery systems used with home-school notes on disruptive/disengaged classroom behavior.	Leblanc	1999	99
S0180		The use of role play and reinforcement procedures in the development of generalized interpersonal behavior with emotionally disturbed-behavior disordered adolescents in a special education classroom.	Lebsock	1981	0
S0181		Individual, generalized, and moderated effects of the good behavior game on at-risk primary school students: A multilevel multiple baseline study using behavioral progress monitoring	Leidig	2022	3
S0183		Replication study of the first step to success early intervention program.	Lien	2005	2
S0184		Programming maintenance for the mystery student intervention.	Litten	2023	3
S0149		Evaluating the boundaries of analytic efficiency and control: A consecutive controlled case series of 26 functional analyses.	Jessel	2020	2
S0185		Behavior disordered children's social skills: Increased by training, but not sustained or reciprocated.	Lovejoy	1988	2

S0186		Using emotes, a social emotional curriculum, to teach elementary aged children with autism spectrum disorder about emotions and perspective taking.	Lowery	2020	99
S0187		Transforming parent-child interaction in family routines: Longitudinal analysis with families of children with developmental disabilities.	Lucyshyn	2015	2
S0188		Using a self-management intervention to increase compliance in children with ASD.	Lui	2014	3
S0158		A cognitive-behavioral treatment for impulse control: A case study.	Kendall	1976	2
S0189		Pharamcotherapy for severe aggression in a child with autism: "Open-label" evaluation of multiple medications on response frequency and intensity of behavioral intervention.	Luiselli	2000	2
S0161	Y	Using assessment-based curricu-lar intervention to improve the classroom behavior of a student with emotional and behav-ioral challenges	Kern	1994	2
S0190		Tootling with a randomized independent group contingency to improve high school classwide behavior.	Lum	2019	3
S0191		The good behaviour game: Maintenance effects	Lynch	2018	3
S0164		The effectiveness of a forgiveness intervention program on reducing adolescents' bullying behavior: A single-case experimental design.	Kim	2017	2
S0192		Generalization and behavior covariation of aggression in children receiving stress inoculation therapy.	Maag	1988	0
S0193		An individual-supported program to enhance placement in a sheltered work environment of autistic individuals mostly with intellectual disability: a prospective observational case series in an Italian community service.	Maggio	2023	2
S0194		Implementing positive behavior support within preschool settings: Group functional assessment and CW-FIT.	Mahon	2020	3
S0171		Reducing disruptive behavior of siblings of children with autism spectrum disorder during treatment sessions.	Krasno	2015	99
S0195		Play your way to compliance: A validation study on a parent training program's effects on compliance in preschoolers with autism spectrum disorder.	Majszak	2018	2
S0196		Dialectical behavioural therapy for oppositional defiant disorder in adolescents: A case series.	Marco	2013	12
S0197		The effects of classroom-based time-in/time-out on compliance rates in children with speech/language disabilities.	Marlow	1997	0
S0198		Examining the efficacy of parent-child interaction therapy with children on the autism spectrum.	Masse	2016	0
S0199		Examining the efficacy of parent-child interaction therapy with high-functioning autism.	Masse	2010	6
S0200		Modified parent-child interaction therapy to address subclinical levels of behavioral concerns: A nonconcurrent multiple probe across participants design.	Mathes	2022	4

S0201		Reducing disruptive behavior in an urban school cafeteria: An extension of the Good Behavior Game	McCurdy	2009	4
S0202		Examining the additive effects of check-in/check-out to coping power.	McDaniel	2019	0
S0182		Multiple-baseline design on pretend game for the rectification of children's aggressive behavior.	Li	2008	99
S0203		Combination long-acting injectable (LAI) antipsychotic medication in adolescents with severe psychosis and aggression: A case series.	McInnis	2019	3
S0206		The effects of social skills instruction and parent participation on aggressive behaviors, antisocial behaviors, and prosocial skills exhibited by primary-age students.	Middleton	1995	6
S0207		The effects of social skills instruction and parental involvement on the aggressive behaviors of African American males.	Middleton	1995	0
S0208		Decreasing severe behavior problems in children with developmental disabilities via training parents in functional assessment.	Miles	2003	99
S0209		Effects of Theraplay on behavior and caregiver relationships in a child with Chiari malformation.	Miller	2018	99
S0210		Evaluating the effectiveness of the tootling intervention in the preschool setting.	Miller	2019	3
S0211	Y	The effects of check-in/check-out on problem behavior and academic engagement in elementary school students.	Miller	2015	2
S0212		Effects of check-in check-out intervention for an African American child with attention deficit hyperactivity disorder.	Mohn	2020	2
S0213		The impact of the contextual fit enhancement protocol on behavior support plan fidelity and student behavior.	Monzalve	2021	1
S0214		A positive version of the Good Behavior Game in a self-contained classroom for EBD: Effects on individual student behavior.	Moore	2022	0
S0215		A program evaluation of the "getting what you want program": A treatment program for adolescents with externalizing behavior disorders.	Moore	2004	99
S0216		The effects of social skill instruction and self-monitoring on came-related behaviors of adolescents with emotional or behavioral disorders	Moore	1995	1
S0217		Teaching behaviorally disordered students to increase teacher attention and praise in mainstreamed classrooms.	Morgan	1983	3
S0218		Using the teaching interaction procedure to train preschool teachers to use fixed-time attention schedules with students with histories of adverse childhood experiences.	Morgan	2021	99
S0220		Positive family intervention for three families of children with Autism Spectrum Disorder: An examination of parental beliefs.	Mueller	2016	99
S0222		Reducing disruptive behaviors in students with serious emotional disturbance.	Musser	2001	3
S0223		Conjoint behavioral consultation as an intervention for young children with disruptive behaviors.	Myers	1997	99

S0204		Adlerian play therapy with students with externalizing behaviors and poor social skills.	Meany	2016	1
S0205		Group Adlerian play therapy with children with off-task behaviors.	Meany	2015	1
S0225		An Exposure-Based Cognitive-Behavioral Therapy for Youth with Severe Irritability: Feasibility and Preliminary Efficacy	Naim	2023	3
S0227		Functional communication training for toddlers at-risk for autism with early problem behavior.	Neely	2022	2
S0229	Y	The effects of physical time-out on the aggressive behaviors of a severely emotionally disturbed child in a public school setting	Noll	1979	2
S0230		The Effect of Multimodal Trainings on Attention Deficit Hyperactivity Disorder	ñzmen	2011	99
S0232		A real-world application of Social Stories as an intervention for children with communication and behaviour difficulties.	O’Connor	2019	1
S0233		Evaluation of Video Feedback and Self- Management to Decrease Schoolyard Aggression and Increase Pro-Social Behaviour in Two Students with Behavioural Disorders.	O'Reilly	2005	0
S0234		Implementing conjoint behavioral consultation for African American children from a low-SES, urban setting.	Ohmstede	2015	0
S0235		The Good Behavior Game for Latino English Language Learners in a small-group setting.	Ortiz	2017	2
S0236		Attempting to Reduce Anger: An International Evaluation of a Forgiveness Based Therapeutic Intervention for Students Who Bully Others.	Owens	2018	1
S0238		Behavior change strategies for reducing disruptive classroom behavior.	Page	1978	3
S0239		Management and prevention of hyperactivity and conduct disorders in 8-10 year old boys through correspondence training procedures.	Paniagua	1990	3
S0219		Positive family intervention for children with ASD: Impact on parents' cognitions and stress.	Mueller	2020	2
S0240		Management of a hyperactive-conduct disordered child through correspondence training: A preliminary study.	Paniagua	1990	2
S0221		Examining the effects of the personal matrix activity with diverse students.	Muldrew	2021	3
S0241		Clinical experience using intranasal ketamine in the treatment of pediatric bipolar disorder/fear of harm phenotype.	Papolos	2013	3
S0243		Cognitive-behavioral intervention in a case of children's jealousy	Pereda	2016	99
S0224		Early intervention using function-based planning for children at-risk for emotional or behavioral disorders.	Nahgahgwon	2008	2
S0244		The effects of therapeutic morning meetings as a socio-emotional, behavioral, and academic intervention on middle school students with emotional disturbance.	Pfitzer	2011	99
S0226		The class specific effects of compliance training with "do" and "don't" requests: Analogue analysis and classroom application.	Neef	1983	2

S0245		The effect of child-centered play therapy on the externalizing behaviors of low-income male preschoolers: A single-case design study.	Phipps	2020	2
S0228		Tiers 1 and 2 of a German MTSS: impact of a multiple baseline study on elementary school students with disruptive behavior	Nitz	2023	3
S0246		Haptotherapy for children and parents: A case-based time-series study of angry outbursts, acceptance, affective parent-child interaction and parenting stress.	Pollmann	2018	0
S0247		A modified self-modeling intervention to reduce disruptive behavior of elementary school boys with social-emotional maladjustment.	Possell	1995	99
S0231		The Effects of Different Rates of Behavior-Specific Praise in Secondary Classrooms	O	2023	2
S0248		Self-modeling as an intervention to reduce disruptive classroom behavior.	Possell	1999	0
S0249		Addressing the social emotional needs of children in chronic poverty: A pilot of the Journey of Hope.	Powell	2019	3
S0250		Efficacy of the daily report card intervention for high-functioning children with autism spectrum disorder: A multiple baseline study.	Pyle	2018	2
S0237		Brief report of efficacy and side effect profile of crossing over to modified-release capsules of methylphenidate in ADHD patients receiving other treatments: Case series.	Ozbaran	2017	3
S0251		Effects of a brief mindfulness-infused behavioral parent training for mothers of children with autism spectrum disorder	Raulston	2019	0
S0252		Video self-modelling: An intervention for children with behavioural difficulties.	Regan	2017	12
S0253		Generalization of cooperative behavior across classroom situations.	Reisinger	1978	0
S0254		Functional assessment-based interventions for children at-risk for emotional and behavioral disorders.	Restori	2007	1
S0242		Facilitating early intervention through teacher training in brief functional behavior assessment.	Peet	2021	2
S0255		The efficacy of a response cost-based treatment package for managing aggressive behavior in preschoolers.	Reynolds	1997	0
S0256		The impact of embedding behavioral supports into reading instruction for upper elementary students with reading difficulties and inattention.	Roberts	2023	2
S0257		Effects of positive statements made by peers on peer interactions and social status of children in a residential treatment setting.	Robinson	1999	99
S0258		Functional analytic psychotherapy among mothers with children with disruptive behavior.	Romero	2018	0
S0259		Family-centered, feedback-informed therapy for conduct disorder: Findings from an empirical case study.	Ronan	2016	3
S0260		Imparting Self-Control Skills to Decrease Aggressive Behavior in a 12-Year-Old Boy: A Case Study.	Ronen	2004	3

S0261		DBT-skills system for cognitively challenged individuals with self-harm: a Swedish pilot study	Rosendahl	2023	2
S0262		Check-In Check-Out + Social Skills: Enhancing the effects of Check-In Check-Out for students with social skill deficits.	Ross	2015	0
S0263		Teacher Child Interaction Training as a universal prevention program in preschool and kindergarten classrooms.	Rossi	2016	2
S0265		Compliance training and behavioral covariation in the treatment of multiple behavior problems.	Russo	1981	2
S0266		Effects of precision requests as a standalone intervention on noncompliance of students with emotional disturbance.	Sabey	2020	3
S0267		Video Self-Modeling to Treat Aggression in Students Significantly Impacted by Autism Spectrum Disorder	Sadler	2019	0
S0268		Being nice to Bobo: The use of three prosocial modeling interventions to decrease physical aggression and increase prosocial behavior among preschoolers with behavior disorders.	Sanborn	2006	99
S0269		Effects of matching instruction difficulty to reading level for students with escape-maintained problem behavior.	Sanford	2013	2
S0264		Reduction of disruptive behaviors using an intervention based on the good behavior game and the say-do-report correspondence.	Ruiz	2010	3
S0270		Modifying delinquents' conversation using token reinforcement of self-recorded behavior.	Sanson	1978	3
S0271		The effects of classwide self-management on students at risk for behavior disorders in general education classrooms.	Sawka	1998	99
S0272		Decreasing disruptive behaviors of children with autism using social stories.	Scattone	2002	2
S0273		Use of the mystery motivator for a high school class.	Schanding	2010	2
S0274		Evaluating the effects of positive reinforcement, instructional strategies, and negative reinforcement on problem behavior and academic performance: An experimental analysis.	Schieltz	2020	1
S0275		Single-case analysis to determine reasons for failure of behavioral treatment via telehealth.	Schieltz	2018	2
S0276		Generalized Reduction of Problem Behavior of Young Children With Autism: Building Trans-Situational Interventions.	Schindler	2005	2
S0277		Therapist perceptions of relationship conditions in child-centered play therapy.	Schottelkorb	2014	2
S0281		Using the prevent-teach-reinforce model with families of young children with ASD.	Sears	2013	2
S0282		Effectiveness of Theraplay in Internalizing and Externalizing Problems in Bereaved Siblings of Children with Cancer	Sepehrtaj	2021	0

S0283		Increasing family safety and decreasing parental stress and child's social-emotional problems with resolutions approach: A single-case experimental design study protocol.	Sepers	2020	4
S0285		MULTI DISCIPLINARY APPROACH IN TREATING A GIRL CHILD DIAGNOSED WITH ATTENTION DEFICIT HYPER ACTIVE DISORDER AND OPPOSITIONAL DEFIANT DISORDER. A CASE REPORT	Shaik	2015	3
S0278		The use of video priming to reduce disruptive transition behavior in children with autism.	Schreibman	2000	2
S0279		Student interview-informed behavior contracts for high school students identified as at risk.	Schrieber	2023	2
S0280		Evaluating the effectiveness of the core content of The Incredible Years with and without visual performance feedback for parents of children with autism.	Schultz	2013	1
S0286	Y	Effects of using self-recording and self-observation in reducing disruptive behavior	Shear	1993	2
S0287		Mindfulness training for adolescents with ADHD and their families: A time-series evaluation.	Shecter	2015	2
S0284		Soles of the feet mindfulness-based program for students with autism spectrum disorder and challenging behavior.	Shah	2022	2
S0289		Classroom-based functional analysis and intervention for disruptive and off-task behaviors.	Shumate	2010	2
S0290		The impact of maternal responsiveness and parent training with conduct-disordered children.	Sierra	2008	99
S0291		Functional communication training for the treatment of multiply determined challenging behavior in two boys with autism	Sigafoos	1996	2
S0288	Y	Decreasing violent or aggressive theme play among preschool children with behavior disorders	Sherburne	1988	2
S0292		A mindfulness-based intervention for self-management of verbal and physical aggression by adolescents with Prader-Willi syndrome.	Singh	2017	2
S0293		Adolescents with Asperger syndrome can use a mindfulness-based strategy to control their aggressive behavior.	Singh	2011	0
S0294		Adolescents with conduct disorder can be mindful of their aggressive behavior.	Singh	2007	0
S0295	Y	Mindfulness Training for Teachers Changes the Behavior of Their Preschool Students	Singh	2013	3
S0296		Surfing the Urge: An informal mindfulness practice for the self-management of aggression by adolescents with autism spectrum disorder.	Singh	2019	0
S0297	Y	Training in Mindful Caregiving Transfers to Parent–Child Interactions	Singh	2009	2
S0298	Y	Mindful Parenting Decreases Aggression, Noncompliance, and Self-Injury in Children With Autism.	Singh	2006	2
S0299		Improving child compliance through self-instructional parent training materials.	Sloane	1990	3

S0301		A clinical case series of six extremely aggressive youths treated with olanzapine.	Soderstrom	2002	3
S0300		Effects of cognitive-behavioral training on angry behavior and aggression of three elementary-aged students.	Smith	1994	0
S0302		Behavioral treatment of stereotypy in children with autism spectrum disorder: A comparative analysis.	Spector	2018	99
S0303		Group art therapy with eighth-grade students transitioning to high school.	Spier	2010	3
S0304		Direct and collateral effects of the First Step to Success program.	Sprague	2009	0
S0305		Formative applications of ongoing visual inspection for trial-based functional analysis: A proof of concept	Standish	2021	1
S0306		The effect of teacher-child interaction training on children who are exhibiting disruptive behaviors within the classroom setting.	Stankus	2021	2
S0307		Effects of an indicated prevention program for preschoolers: A single-subject design approach.	Stefan	2015	2
S0308		Outpatient behavioral management of aggressiveness in adolescents: A response cost paradigm.	Stein	1999	3
S0309		The effects of child-centered play therapy on African American boys with aggressive behavior.	Stewart	2020	99
S0310		Aripiprazole for Maladaptive Behavior in Pervasive Developmental Disorders.	Stigler	2004	3
S0311		Teacher implementation of precorrection and praise statements in Head Start classrooms as a component of a program-wide system of positive behavior support.	Stormont	2007	3
S0312		Effects of peer assisted communication application training on the communicative and social behaviors of children with autism.	Strasberger	2014	1
S0313		Impact of Adlerian play therapy on externalizing behaviors of at-risk preschoolers.	Stutey	2017	0
S0314		The effects of training students with learning and behavior disorders to modify the behavior of their peers.	Sugai	1989	1
S0315		Effectiveness of group play therapy on problematic behaviors and symptoms of anxiety of preschool children.	Swan	2019	1
S0319	Y	Cognitive-behavior modification treatment of an aggressive 11-year-old boy	Taber	1981	0
S0316		Initial investigation of nature-based, child-centered play therapy: A single-case design.	Swank	2015	3
S0321		Improving social skills through paper and digital social stories(tm).	Teague	2015	99
S0317		Nature-based child-centered group play therapy and behavioral concerns: A single-case design.	Swank	2017	2
S0318		On-Task Behavior of Children With Attention-Deficit/Hyperactivity Disorder: Examining Treatment Effectiveness of Play Therapy Interventions	Swank	2018	2
S0320		Potential usefulness of the kampo medicine yokukansan, containing uncaria hook, for paediatric emotional and behavioural disorders: a case series.	Tanaka	2013	3

S0322		Exploring the effects of a wearable biocueing app (Sense-IT) as an addition to aggression regulation therapy in forensic psychiatric outpatients	ter	2023	2
S0326		Self-monitoring and self-recruited praise: Effects on problem behavior, academic engagement, and work completion in a typical classroom.	Todd	1999	3
S0323		Therapeutic assessment with children: A pilot study of treatment acceptability and outcomes.	Tharinger	2009	3
S0324		The effects of remote acceptance and commitment therapy on parent and child behavior.	Tilson	2023	99
S0325		Parent-Child Interaction Therapy: Application of an Empirically Supported Treatment to Maltreated Children in Foster Care.	Timmer	2006	12
S0327	Y	The effects of a targeted intervention to reduce problem behaviors: Elementary school implementation of Check In-Check Out	Todd	2008	2
S0333		Multimodal play therapy for children, 6-8 years old, with tics: A single-case design and analysis.	Vessels	2019	2
S0334		The effects of behavioral momentum on task initiation, math performance, and problematic behaviors of residential youth.	Vincent	1997	2
S0328	Y	Using social stories to change problematic lunchtime behaviour in school	Toplis	2006	1
S0329		Managing middle school students' disruptive behaviors during physical education classes using the color wheel system.	Tounsi	2023	0
S0338		Coaching paraeducators to implement functional communication training involving augmentative and alternative communication for students with autism spectrum disorder	Walker	2021	2
S0330		The cultural adaptation of teachers and parents as partners (TAPP) to Brazil: Efficacy and acceptability.	Trefiglio	2023	99
S0331		Poor peer interactions and social isolation: A case report of successful in vivo social skills training on a child psychiatric inpatient unit.	Van Hasselt	1984	0
S0332		Training teachers to implement classroom pivotal response teaching during small-group instruction: A pilot study.	Verschuur	2021	2
S0335		Interactional intervention in child behavior problems.	Vite	2015	3
S0336		Conducting functional communication training via telehealth to reduce the problem behavior of young children with autism.	Wacker	2013	0
S0337		The effects of parent-child interaction therapy on symptoms and impairment in young children with attention-deficit/hyperactivity disorder.	Wagner	2012	3
S0339	Y	The effect of videotape feedback on the on-task behavior of a student with emotional/behavioral disorders	Walther	1991	1
S0340		The use of a stimulus control transfer procedure to teach spontaneous manding to children with autism.	Ward	2015	2
S0341		A Case Study Examining Fixed Versus Randomized Criteria for Treating a Child With Conduct Problems and Callous-Unemotional Traits.	Waschbusch	2016	0

S0342	Y	Behavior modification with culturally deprived school children: two case studies	Wasik	1969	0
S0343		Enhancing treatment participation in CAMHS among families of conduct problem children: Effectiveness study of a clinician training programme.	Watt	2012	3
S0344		The effectiveness of a Social Story intervention in Decreasing disruptive behavior in autistic children.	Watts	2018	2
S0354		The consultative merits of praise-ignore versus praise-reprimand instruction.	Workman	1980	3
S0345		Contingency contracting with families of delinquent adolescents.	Weathers	1975	0
S0356		Generalized Negatively Reinforced Manding in Children with Autism	Yi	2006	2
S0346		Can less be more for students at-risk for emotional and behavioral disorders: Evaluating components of check-in/check-out.	Weber	2019	2
S0360		Long-term outcome of globus pallidus internus deep brain stimulation in patients with Tourette syndrome.	Zhang	2014	1
S0361		Using class pass intervention to decrease disruptive behavior in young children.	Zuniga	2022	2
S0347		Multisystemic therapy (MST) for youth offending, psychiatric disorder and substance abuse: Case examples from a UK MST team.	Wells	2010	3
S0348		Effects of children's literature on students' on-task behavior during mathematics instruction.	Whitney	2012	1
S0349		An evaluation of performance feedback intervention to increase children's physical activity levels and reduce disruptive behavior.	Wicoff	2018	99
S0350		Evaluation of a guided compliance procedure to reduce noncompliance among preschool children.	Wilder	2006	2
S0351		A preliminary investigation of the effect of intervention on parental attributions and reported behaviour	Wilson	2006	3
S0352		Teaching cooperative play to behavior-problem preschool children.	Wolfe	1983	1
S0353		Treatment of comorbid conduct problems and depression in youth: A pilot study.	Wolff	2012	3
S0355		The effects of the class-wide function-related intervention teams on behaviors of an elementary student with autism spectrum disorder in an inclusive classroom in Taiwan.	Wu	2019	2
S0357		A study on the effects of a parent training program on the behavior of foster parents and their foster children.	Yockelson	2000	99
S0358	Y	Modification of a child's problem behaviors in the home with the mother as therapist.	Zeilberger	1968	0
S0359		A Precision-Based Approach to Implement Evidence-Based Interventions for Students With Externalizing Behaviors in Developing Countries: A Single Case Experimental Study in China	Zhang	2023	0
S0362	NS	Applying Multitiered Support for Professional Development of Targeted Student Interventions: A Single-Case Design Stud	Samudre	2024	0

S0363	NS	Effects of Banking Time Intervention on Child-Teacher Relationship and Problem Behavior in China: A Multiple Baseline Design	Zhang	2024	0

Legend:

0=	Included
Y=	Included during grey literature search
1 =	wrong target or outcome behavior
10 =	reason unknown / not stated in inlcusion exclusion file
11 =	not screened yet
12 =	only pre and post
2 =	wrong target group (i.e intellectual diability, ADHD, autism, too old/ young, etc)
3 =	wrong kind of data
4 =	wrong publication type (panel study, preventive study, study protocol)
5 =	no psychotherapy
6 =	duplication of other study within sample
7 =	excluded after screening pdfs
77 =	excluded after screening pdfs of grey literature search
99 =	Paper not found
NS=	New search
