# Supplementary material for: A Multilevel Meta-Analysis of Single-Case Research on Interventions for Externalizing Behavior Problems in Children and Adolescents
Source: JAACAP Open. 2025 Dec 18;4(2):220–31. doi: 10.1016/j.jaacop.2025.12.002 (PMC13043497; doi:10.1016/j.jaacop.2025.12.002)
Supplement: Supplemental Data [file mmc7.pdf]

Supplement #3A – Study Characteristics Part 1

study_id	study_author	study_year	pub_type	study_country	study_n	study_dropout	sample_type	study_setting
S0005	Alonso	2022	article	Spain	1	0	referred	private practice
S0007	Anderson	2019	dissertation	USA	4	NR	referred	video-conference
S0013	Ascanio	2018	article	Spain	1	0	referred	Outpatient Psychiatric Center
S0016	Axelrod	2014	article	USA	3	NR	referred	Acute Care Psychiatric Hospital
S0017	Bahl	2001	dissertation	USA	6	NR	referred	school
S0024	Bellinger	2012	dissertation	USA	3	NR	referred	school / home
S0026	Benish	2011	article	USA	3	NR	referred	school
S0028	Bice	2016	article	USA	6	NR	referred	school
S0037	Bornstein	1980	article	USA	4	NR	referred	Psychiatric Center
S0040	Bourn	1993	article	UK	1	NR	referred	Outpatient Psychiatric Center
S0047	Buzenski	2018	dissertation	USA	9	6	referred	school
S0048	Campbell	2023	article	USA	12	NR	referred	school
S0050	Carter	2009	article	USA	3	NR	referred	school
S0051	Carter	2007	article	USA	1	NR	referred	school
S0062	Conoley	2003	article	USA	4	NR	voluntarily	university-affiliated clinic
S0062	Conoley	2003	article	USA	4	NR	voluntarily	university-affiliated clinic
S0070	Danforth	2001	article	USA	3	NR	referred	university-affiliated clinic
S0071	Danforth	1998	article	USA	8	NR	referred	university-affiliated clinic
S0075	Davis	1979	article	USA	1	2	referred	school
S0084	DiGangi	1992	article	USA	3	NR	referred	school
S0096	Ennis	2012	article	USA	6	NR	referred	school
S0101	Ferro	2017	article	Spain	1	NR	referred	Outpatient Psychiatric Center
S0108	Furuzawa	2020	article	Japan	1	NR	referred	Outpatient Psychiatric Center
S0113	Gettinger	2021	article	USA	8	2	referred	school
S0116	Gordon	2016	article	USA	1	NR	referred	university-affiliated clinic
S0120	Greene	1999	article	USA	5	1	voluntarily	home

S0122	Guerra	2011	article	Chile	3	NR	referred	school / home
S0123	Gullon	2019	article	USA	19	NR	voluntarily	home
S0124	Gumpel	2000	article	Israel	3	NR	referred	school
S0125	Gunter	1994	article	USA	1	0	referred	school
S0129	Hai	2021	article	USA	11	3	referred	school
S0134	Harding	2009	article	USA	3	NR	referred	home
S0136	Hodges	2022	article	USA	3	NR	voluntarily	home
S0136	Hodges	2022	article	USA	3	NR	voluntarily	home
S0138	Holyfield	2022	dissertation	USA	3	NR	referred	home
S0147	Jeffries	2016	dissertation	USA	4	2	referred	university-affiliated clinic
S0153	Karhu	2019	article	Finland	3	NR	referred	school
S0154	Karhu	2021	article	Finland	4	NR	referred	school
S0155	Katic	2023	dissertation	USA	3	NR	referred	home
S0156	Kehle	1986	article	USA	4	NR	referred	school
S0159	Kern	1995	article	USA	8	NR	referred	school
S0165	Kimonis	2012	article	USA	1	NR	referred	university-affiliated clinic
S0167	Knap	2018	dissertation	USA	1	2	referred	university-affiliated clinic
S0170	Krasch	2015	dissertation	USA	4	NR	referred	school
S0173	Kumm	2020	dissertation	USA	4	NR	referred	school
S0180	Lebsock	1981	article	USA	2	NR	referred	school
S0192	Maag	1988	article	USA	4	NR	referred	school
S0192	Maag	1988	article	USA	4	NR	referred	school
S0197	Marlow	1997	article	USA	4	NR	referred	school
S0198	Masse	2016	article	USA	5	2	referred	home
S0202	McDaniel	2019	article	USA	3	NR	referred	school
S0207	Middleton	1995	article	USA	9	4	referred	school
S0214	Moore	2022	article	USA	4	1	referred	school
S0233	O'Reilly	2005	article	USA	2	NR	referred	school
S0234	Ohmstede	2015	article	USA	6	3	referred	school / home

S0246	Pollmann	2018	article	The Netherlands	1	NR	referred	Outpatient Psychiatric Center
S0248	Possell	1999	article	USA	4	NR	referred	school
S0251	Raulston	2019	article	USA	4	1	voluntarily	home
S0253	Reisinger	1978	article	USA	3	NR	referred	school
S0255	Reynolds	1997	article	USA	4	NR	voluntarily	school
S0258	Romero	2018	article	Colombia	5	NR	referred	university-affiliated clinic
S0262	Ross	2015	article	USA	5	NR	referred	school
S0267	Sadler	2019	article	USA	3	NR	referred	school
S0282	Sepehrtaj	2021	article	Iran	4	NR	referred	university-affiliated clinic
S0293	Singh	2011	article	unclear	3	NR	referred	home
S0294	Singh	2007	article	unclear	3	NR	referred	school
S0296	Singh	2019	article	unclear	3	NR	referred	home
S0300	Smith	1994	article	USA	3	NR	referred	school
S0304	Sprague	2009	article	USA	4	NR	referred	school
S0313	Stutey	2017	article	USA	4	NR	voluntarily	school
S0319	Taber	1981	article	USA	1	NR	referred	Psychiatric Center
S0329	Tounsi	2023	article	Tunisia	17	6	referred	school
S0331	Van Hasselt	1984	article	USA	1	NR	referred	university-affiliated clinic
S0336	Wacker	2013	article	USA	17	9	referred	Outpatient Psychiatric Center
S0341	Waschbusch	2016	article	USA	1	NR	referred	university-affiliated clinic
S0342	Wasik	1969	article	USA	2	NR	referred	school
S0345	Weathers	1975	article	USA	28	16	referred	home
S0358	Zeilberger	1968	article	USA	1	NR	referred	home
S0359	Zhang	2023	dissertation	China	6	NR	referred	school
S0362	Samudre	2024	article	USA	3	NR	referred	school
S0363	Zhang	2024	article	China	8	NR	referred	school

Supplement #3B – Study Characteristics Part 2

study_id	sced_design	data_availability
S0005	AB	f.a.
S0007	Multiple-baseline	f.a.
S0013	AB	f.a.
S0016	Multiple-baseline	pre- and post-test; f.a.
S0017	Multiple-baseline	f.a.
S0024	Multiple-baseline	pre- and post-test; f.a.
S0026	ABC	f.a.
S0028	Multiple-baseline	pre- and post-test; f.a.
S0037	AB	f.a.
S0040	Multiple-baseline	pre- and post-test; f.a.
S0047	Multiple-baseline	pre- and post-test; f.a.
S0048	Multiple-baseline	pre- and post-test; f.a.
S0050	Multiple-baseline	pre- and post-test; f.a.
S0051	ABAB	pre- and post-test; f.a.
S0062	Multiple-baseline	pre- and post-test; f.a.
S0070	Multiple-baseline	pre- and post-test; f.a.
S0071	Multiple-baseline	f.a.
S0075	ABAB	f.a.
S0084	"A-B-BC-C-D-DB-DBC-DC"	mean appropriate and inappropriate verbal behavior scores across experimental conditions; f.a.
S0096	Multiple-baseline	f.a.
S0101	AB	f.a.
S0108	AB	f.a.
S0113	Multiple-baseline	f.a.
S0116	Multiple Baseline Design	pre- and post-test; f.a.
S0120	Multiple-baseline	f.a.
S0122	Multiple-baseline	f.a.
S0123	ABAB	f.a.
S0124	Multiple-baseline	f.a.

S0125	ABAB	f.a.
S0129	AB	f.a.
S0134	Multiple-baseline	f.a.
S0136	Multiple-baseline	f.a.
S0138	Multiple-baseline	pre- and post-test; f.a.
S0147	Multiple Baseline Design	pre- and post-test; f.a.
S0153	Multiple-baseline	f.a.
S0154	Multiple-baseline	f.a.
S0155	Multiple-baseline	pre- and post-test; f.a.
S0156	ABA	f.a.
S0159	Multiple Baseline Design	f.a.
S0165	ABC	f.a.
S0167	AB	pre- and post-test; f.a.
S0170	Multiple-baseline	f.a.
S0173	ABAB	pre- and post-test; f.a.
S0180	Multiple-baseline	f.a.
S0192	Multiple-baseline	f.a.
S0197	Multiple-baseline	f.a.
S0198	Multiple-baseline	pre- and post-test; f.a.
S0202	ABBCBBC	pre- and post-test; f.a.
S0207	Multiple-baseline	f.a.
S0214	ABAB	f.a.
S0233	Multiple-baseline	f.a.
S0234	Multiple-baseline	pre- and post-test; f.a.
S0246	Multiple-baseline	pre- and post-test; f.a.
S0248	Multiple-baseline	pre- and post-test; f.a.
S0251	Multiple-baseline	f.a.
S0253	Multiple-baseline	f.a.
S0255	Multiple-baseline	f.a.
S0258	AB	f.a.
S0262	Multiple-baseline	pre- and post-test; f.a.

S0267	ABCA	pre- and post-test; f.a.
S0282	Multiple-baseline	pre- and post-test; f.a.
S0293	Multiple-baseline	f.a.
S0294	Multiple-baseline	pre- and post-test; f.a.
S0296	Multiple-baseline	f.a.
S0300	Multiple-baseline	f.a.
S0304	Multiple-baseline	pre- and post-test; f.a.
S0313	ABC	f.a.
S0319	Multiple-baseline	pre- and post-test; f.a.
S0329	Multiple-baseline	f.a.
S0331	AB	f.a.
S0336	Multiple-baseline	f.a.
S0341	AB	f.a.
S0342	ABAC	pre- and post-test; f.a.
S0345	Multiple-baseline	f.a.
S0358	ABAC	f.a.
S0359	Multiple-baseline	f.a.
S0362	Multiple-baseline	f.a.
S0363	Multiple-baseline	pre- and post-test; f.a.

Supplement #3C – Child Characteristics Part 1

study_id	child_id	child_name	child_age	child_sex	child_living	child_ethnicity
S0005	C0001	J	9	M	at home	NR
S0007	C0002	Participant A	15	M	at home	caucasian
S0007	C0003	Participant B	15/16	M	at home	caucasian
S0007	C0004	Participant C	15	M	at home	caucasian
S0007	C0005	Participant D	14	M	at home	caucasian
S0013	C0006	Marcos	10	M	at home	NR
S0016	C0013	Dan	8	M	psychiatric hospital	caucasian
S0016	C0014	Bob	8	M	psychiatric hospital	caucasian
S0016	C0015	Tom	7	M	psychiatric hospital	caucasian
S0017	C0016	Jill	4	F	NR	caucasian
S0017	C0017	Tom	4	M	NR	caucasian
S0017	C0018	Jack	4	M	NR	caucasian
S0017	C0019	Alex	4	M	NR	caucasian
S0017	C0020	Kathy	4	F	NR	caucasian
S0017	C0021	Sam	4	F	NR	caucasian
S0024	C0025	CR	7	m	at home	caucasian
S0024	C0026	DW	6	m	foster family	caucasian
S0024	C0027	LM	7	m	at home	caucasian
S0026	C0028	Lisa	4	f	NR	NR
S0026	C0029	Kyle	4	m	NR	NR
S0026	C0030	John	4	m	NR	NR
S0028	C0031	Student A	7	m	NR	caucasian
S0028	C0032	Student B	10	m	NR	caucasian
S0028	C0033	Student C	9	m	NR	caucasian
S0028	C0034	Student D	5	m	NR	caucasian
S0028	C0035	Student E	5	m	NR	caucasian
S0028	C0036	Student F	9	m	NR	caucasian
S0037	C0039	Mike	8	m	currently living at psychiatric intensive care	afro-american

S0037	C0041	Tom	12	m	currently living at psychiatric intensive care	mixed ethnic ancestry
S0037	C0042	Sue	12	f	currently living at psychiatric intensive care	caucasian
S0040	C0044	Scott	4.5	m	at home	NR
S0047	C0045	Student 1	6	f	NR	afro-american
S0047	C0046	Student 2	6	m	NR	afro-american
S0047	C0047	Student 3	6	m	NR	caucasian
S0048	C0048	Student 1	6-11	m	NR	afro-american
S0048	C0049	Student 2	6-11	m	NR	afro-american
S0048	C0050	Student 3	6-11	m	NR	afro-american
S0048	C0051	Student 4	6-11	m	NR	afro-american
S0048	C0052	Student 5	6-11	m	NR	afro-american
S0048	C0053	Student 6	6-11	m	NR	afro-american
S0048	C0054	Student 7	6-11	m	NR	afro-american
S0048	C0055	Student 8	6-11	m	NR	afro-american
S0048	C0056	Student 9	6-11	m	NR	afro-american
S0048	C0057	Student 10	6-11	m	NR	afro-american
S0048	C0058	Student 11	6-11	m	NR	afro-american
S0048	C0059	Student 12	6-11	m	NR	afro-american
S0050	C0060	Gabriel	6	m	first living at home, however moved to foster care during intervention	caucasian
S0050	C0061	Jonas	7	m	at home	caucasian
S0050	C0062	Patrick	5	m	at home	caucasian
S0051	C0063	Noah	6	m	at home	caucasian
S0062	C0064	Tim	9	m	at home	caucasian
S0062	C0065	Arnie	9	m	at home	caucasian
S0062	C0066	Jon	8	m	at home	caucasian
S0070	C0068	Subject 1	5.9	m	at home	NR
S0070	C0069	Subject 2	6.4	m	at home	NR
S0070	C0070	Subject 3	6.4	m	at home	NR
S0071	C0071	Child 1	4,0 - 7,4	f	at home	NR
S0071	C0072	Child 2	4,0 - 7,4	m	at home	NR
S0071	C0073	Child 3	4,0 - 7,4	m	at home	NR

S0071	C0074	Child 4	4,0 - 7,4	f	at home	NR
S0071	C0075	Child 5	4,0 - 7,4	m	at home	NR
S0071	C0076	Child 6	4,0 - 7,4	f	at home	NR
S0071	C0077	Child 7	4,0 - 7,4	m	at home	NR
S0071	C0078	Child 8	4,0 - 7,4	m	at home	NR
S0075	C0091	Eric	11	m	NR	NR
S0084	C0092	Subject 1	12	m	NR	caucasian
S0084	C0093	Subject 2	13	m	NR	caucasian
S0096	C0100	Evander	13	m	residential facility	afro-american
S0096	C0101	Ethan	16	m	residential facility	afro-american
S0096	C0102	Elsa	14	f	residential facility	caucasian
S0096	C0103	Patrick	12	m	residential facility	caucasian
S0096	C0104	Priscilla	15	f	residential facility	caucasian
S0096	C0105	Peter	13	m	residential facility	afro-american
S0101	C0106	Pablo	4	m	at home	NR
S0108	C0107	Akira	5	m	at home	Japanese
S0113	C0109	Student 1	Grade 2 (7,0-8,0)	NR	NR	NR
S0113	C0110	Student 2	Grade 2 (7,0-8,0)	NR	NR	NR
S0113	C0111	Student 3	Grade 2 (7,0-8,0)	NR	NR	NR
S0113	C0112	Student 4	Grade 2 (7,0-8,0)	NR	NR	NR
S0113	C0113	Student 5	Grade 2 (7,0-8,0)	NR	NR	NR
S0113	C0114	Student 6	Grade 2 (7,0-8,0)	NR	NR	NR
S0116	C0115	Katie	4	f	at home	NR
S0120	C0116	John	7	m	at home	hispanic
S0120	C0117	Amy	7	f	at home	hispanic
S0120	C0118	Mark	5	m	at home	afro-american
S0120	C0119	Victor	7	m	at home	hispanic
S0122	C0120	Nino 1	3,6 - 4,6	m	at home	NR
S0122	C0121	Nino 2	3,6 - 4,6	m	at home	NR
S0122	C0122	Nino 3	3,6 - 4,6	m	at home	NR
S0123	C0126	Jenny	7	f	at home	NR

S0123	C0128	Luke	7	m	at home	NR
S0123	C0131	Megan	9	f	at home	NR
S0123	C0132	Mia	5	f	at home	NR
S0124	C0142	Yitshak	9	m	NR	NR
S0124	C0143	Ronen	9	m	NR	NR
S0125	C0145	Tom	12	m	NR	NR
S0129	C0146	Subject 1	10,0 - 11,0	NR	NR	NR
S0129	C0147	Subject 2	10,0 - 11,0	NR	NR	NR
S0129	C0148	Subject 3	10,0 - 11,0	NR	NR	NR
S0129	C0149	Subject 4	10,0 - 11,0	NR	NR	NR
S0129	C0150	Subject 5	10,0 - 11,0	NR	NR	NR
S0129	C0151	Subject 6	10,0 - 11,0	NR	NR	NR
S0129	C0152	Subject 7	10,0 - 11,0	NR	NR	NR
S0129	C0153	Subject 8	10,0 - 11,0	NR	NR	NR
S0129	C0154	Subject 9	10,0 - 11,0	NR	NR	NR
S0129	C0155	Subject 10	10,0 - 11,0	NR	NR	NR
S0129	C0156	Subject 11	10,0 - 11,0	NR	NR	NR
S0134	C0157	Al	3	m	at home	NR
S0134	C0158	Lou	2.11	m	at home	NR
S0136	C0160	Ronald	2	m	at home	caucasian
S0136	C0161	Kylie	4	f	at home	caucasian
S0136	C0162	Ella	2	f	at home	caucasian
S0138	C0163	Child 1	3	m	at home	caucasian
S0138	C0164	Child 2	2.2	m	at home	hispanic
S0138	C0165	Child 3	2.5	m	at home	caucasian
S0147	C0323	Dyad 2	m = 4,23	m	at home	caucasian
S0147	C0324	Dyad 4	m = 4,23	m	at home	bi-racial
S0153	C0166	John	Grade 2 (7,0-8,0)	m	at home	NR
S0153	C0167	David	Grade 3 (8,0 - 9,0)	m	at home	NR
S0153	C0168	Andrew	Grade 6 (11,0 -12,0)	m	at home	NR
S0154	C0169	Jonah	12	m	NR	NR

S0154	C0170	Kevin	8	m	NR	NR
S0154	C0171	Joe	8	m	NR	NR
S0154	C0172	Rick	10	m	NR	NR
S0155	C0173	Student A	16	m	at home	hispanic
S0155	C0174	Student B	14	f	at home	hispanic
S0155	C0175	Student C	14	m	at home	asian
S0156	C0176	Child 1	10 to 13	m	NR	NR
S0156	C0177	Child 2	10 to 13	m	NR	NR
S0156	C0178	Child 3	10 to 13	m	NR	NR
S0159	C0180	Brian	11	m	NR	NR
S0159	C0181	Chris	10	m	NR	NR
S0159	C0182	Andy	12	m	NR	NR
S0159	C0183	Juan	8	m	NR	NR
S0159	C0184	Miguel	8	m	NR	NR
S0159	C0185	Tommy	12	m	NR	NR
S0159	C0186	Earl	9	m	NR	NR
S0159	C0187	Larry	12	m	NR	NR
S0159	C0188	Susan	13	f	NR	NR
S0159	C0189	Debby	13	f	NR	NR
S0159	C0190	Mara	12	f	NR	NR
S0165	C0191	Luke	6	m	with adopted mother	bi-racial
S0167	C0325	Dyad 1	m = 4,67	f	at home	caucasian
S0170	C0192	Child 1	5.6	m	NR	hispanic
S0170	C0193	Child 2	5.4	m	NR	asian
S0170	C0194	Child 3	4.1	m	NR	caucasian
S0170	C0195	Child 4	4	m	NR	caucasian
S0173	C0196	Korey	17	m	at home	caucasian
S0173	C0197	Deandre	15	m	at home	afro-american
S0173	C0198	Ezekiel	16	m	with grandmother	afro-american
S0173	C0199	Noel	15	m	at home	afro-american
S0180	C0200	Participant 1	14	f	NR	NR

S0180	C0201	Participant 2	15	m	NR	NR
S0192	C0205	Duane	12.2	m	residential treatment facility	NR
S0192	C0206	Jim	13.5	m	residential treatment facility	NR
S0192	C0207	Clarence	12.5	m	residential treatment facility	NR
S0192	C0208	Jesse	11.1	m	residential treatment facility	NR
S0197	C0211	Student 1	11	m	NR	caucasian
S0197	C0212	Student 2	4	f	NR	caucasian
S0197	C0213	Student 3	11	f	NR	caucasian
S0198	C0214	Kenneth	3	m	at home	caucasian
S0198	C0215	Adam	4	m	at home	caucasian
S0198	C0216	Christopher	4	m	at home	caucasian
S0202	C0217	Cory	8	m	NR	afro-american
S0202	C0218	Kareem	10	m	NR	afro-american
S0202	C0219	Jeremiah	11	m	NR	afro-american
S0207	C0220	Student 1	6,0 - 9,0 (m = 8,0)	m	NR	afro-american
S0207	C0221	Student 2	6,0 - 9,0 (m = 8,0)	m	NR	afro-american
S0207	C0222	Student 3	6,0 - 9,0 (m = 8,0)	m	NR	afro-american
S0207	C0224	Student 5	6,0 - 9,0 (m = 8,0)	m	NR	afro-american
S0214	C0225	Ron	Grade 4 (9,0 - 10,0)	m	NR	afro-american
S0214	C0226	Josh	Grade 5 (10,0 - 11,0)	m	NR	caucasian
S0214	C0227	Deidre	Grade 5 (10,0 - 11,0)	f	NR	afro-american
S0233	C0228	Garry	10	m	NR	NR
S0233	C0229	David	10	m	NR	NR
S0234	C0230	Child A	9	m	with grandmother	mixed ethnic ancestry
S0234	C0231	Child B	9	m	with great aunt	afro-american
S0234	C0232	Child C	13	m	at home	afro-american
S0246	C0234	Brit	10	f	at home	NR
S0248	C0235	Student 1	5,0 - 8,0	m	NR	NR
S0248	C0236	Student 2	5,0 - 8,0	m	NR	NR
S0248	C0237	Student 3	5,0 - 8,0	m	NR	NR

S0248	C0238	Student 4	5,0 - 8,0	m	NR	NR
S0251	C0239	Jonathan	4.4	m	at home	caucasian
S0251	C0240	Tabitha	4.9	f	at home	caucasian
S0251	C0241	Richie	6.7	m	at home	caucasian
S0253	C0243	Jamie	3,5 - 4,5	f	at home	NR
S0253	C0244	Stephanie	3,5 - 4,5	f	at home	NR
S0253	C0245	Debbie	3,5 - 4,5	f	at home	NR
S0255	C0246	Jacob	3	m	at home	caucasian
S0255	C0247	Mike	5	m	at home	afro-american
S0255	C0248	Randy	4	m	at home	caucasian
S0255	C0249	Cory	4	m	at home	caucasian
S0258	C0250	Mary's Child	m = 7,2 (SD (1,3)	m	at home	NR
S0258	C0251	Sophie's Child	m = 7,2 (SD (1,3)	m	at home	NR
S0258	C0252	Martha's Child	m = 7,2 (SD (1,3)	m	at home	NR
S0258	C0253	Elsa's Child	m = 7,2 (SD (1,3)	m	at home	NR
S0262	C0255	Lucinda	9	f	NR	hispanic
S0262	C0256	Sarah	9	f	NR	caucasian
S0262	C0257	Emily	11	f	NR	hispanic
S0262	C0258	Olivia	11	f	NR	caucasian
S0262	C0259	Tom	7	m	NR	caucasian
S0267	C0263	Jason	12	m	NR	NR
S0267	C0264	Ryan	10	m	NR	NR
S0267	C0265	Mike	8	m	NR	NR
S0282	C0266	Participant 1	9	f	at home	NR
S0282	C0267	Participant 2	10	m	at home	NR
S0282	C0268	Participant 3	7	f	at home	NR
S0282	C0269	Participant 4	8	f	at home	NR
S0293	C0270	John	15	m	at home	NR
S0293	C0271	Paul	13	m	at home	NR
S0294	C0273	Ricky	14	m	at home	caucasian

S0294	C0274	Kent	13	m	foster family	caucasian
S0294	C0275	Libby	13	f	at home	caucasian
S0296	C0276	Alan	16	m	at home	NR
S0296	C0277	Brady	17	m	at home	NR
S0296	C0278	Cam	17	m	at home	NR
S0300	C0279	Robert	10	m	NR	afro-american
S0300	C0280	Martha	10	f	NR	afro-american
S0300	C0281	Travis	11	m	NR	caucasian
S0304	C0282	Student 1	5	f	NR	caucasian
S0304	C0283	Student 2	5.6	m	NR	caucasian
S0304	C0284	Student 3	5	m	NR	caucasian
S0304	C0285	Student 4	6	m	NR	caucasian
S0313	C0286	Jacylnn	4.5	f	with mother and grandmother	afro-american
S0313	C0287	Jardon	4.5	m	with mother and grandmother	afro-american
S0313	C0288	Malaika	5	f	with mother and grandmother	afro-american
S0313	C0289	Keenan	3.5	m	at home	afro-american
S0319	C352	Steve	11	m	residential treatment facility	NR
S0329	C0291	Student 1	m = 13,33 (SD= 1,1)	m	NR	NR
S0329	C0292	Student 2	m = 13,33 (SD= 1,1)	m	NR	NR
S0329	C0293	Student 3	m = 13,33 (SD= 1,1)	m	NR	NR
S0329	C0294	Student 4	m = 13,33 (SD= 1,1)	m	NR	NR
S0329	C0295	Student 5	m = 13,33 (SD= 1,1)	m	NR	NR
S0329	C0296	Student 6	m = 13,33 (SD= 1,1)	m	NR	NR
S0329	C0297	Student 7	m = 13,33 (SD= 1,1)	m	NR	NR
S0329	C0298	Student 8	m = 13,33 (SD= 1,1)	m	NR	NR
S0329	C0299	Student 9	m = 13,33 (SD= 1,1)	m	NR	NR
S0331	C0300	Bob	7.5	m	foster family	NR
S0336	C0301	Jake	2,5 - 6,6	NR	NR	NR
S0336	C0302	Jack	2,5 - 6,6	NR	NR	NR
S0336	C0303	Jill	2,5 - 6,6	NR	NR	NR
S0336	C0304	Tad	2,5 - 6,6	NR	NR	NR

S0336	C0305	Mitt	2,5 - 6,6	NR	NR	NR
S0336	C0306	Zeke	2,5 - 6,6	NR	NR	NR
S0341	C0307	Juan	8.11	m	with adoption parents	hispanic
S0342	C0308	Dianne	7	f	NR	afro-american
S0342	C0309	Jane	7	f	NR	afro-american
S0345	C0310	Child 1	14,0 - 17,0	NR	NR	NR
S0345	C0311	Child 2	14,0 - 17,0	NR	NR	NR
S0345	C0312	Child 3	14,0 - 17,0	NR	NR	NR
S0345	C0313	Child 4	14,0 - 17,0	NR	NR	NR
S0345	C0314	Child 5	14,0 - 17,0	NR	NR	NR
S0345	C0315	Child 6	14,0 - 17,0	NR	NR	NR
S0358	C0316	Rorey	5	m	home	NR
S0359	C0317	Yang	m = 8,5 (SD = 6,14)	m	NR	Han
S0359	C0318	Zong	m = 8,5 (SD = 6,14)	m	NR	Han
S0359	C0319	Zhao	m = 8,5 (SD = 6,14)	m	NR	Han
S0359	C0320	Ning	m = 8,5 (SD = 6,14)	m	NR	Han
S0359	C0321	Zhu	m = 8,5 (SD = 6,14)	m	NR	Han
S0359	C0322	Chen	m = 8,5 (SD = 6,14)	m	NR	Han
S0362	C0341	Chase	7	m	NR	Mixed (caucasian/ afro-american)
S0362	C0342	Kamari	7	m	NR	afro-american
S0362	C0343	Alonzo	9	m	NR	afro-american
S0363	C0344	Child 1	4	m	NR	NR
S0363	C0345	Child 2	4	f	NR	NR
S0363	C0346	Child 3	4	m	NR	NR
S0363	C0347	Child 4	4	m	NR	NR
S0363	C0348	Child 5	4	f	NR	NR
S0363	C0349	Child 6	4	m	NR	NR

Supplement #3D – Child Characteristics Part 2

study_id	child_id	primary_diagnosis	diagnosis_screening	child_medication
S0005	C0001	not assessed	NR	No
S0007	C0002	not assessed	NR	No
S0007	C0003	not assessed	NR	No
S0007	C0004	not assessed	NR	No
S0007	C0005	not assessed	NR	No
S0013	C0006	ODD	screened	No
S0016	C0013	ODD, ADHD	screened	Adderall XR
S0016	C0014	ODD	screened	No
S0016	C0015	ODD, ADHD	screened	Concerta
S0017	C0016	None	screened	No
S0017	C0017	None	screened	No
S0017	C0018	None	screened	No
S0017	C0019	None	screened	No
S0017	C0020	None	screened	No
S0017	C0021	None	screened	No
S0024	C0025	not assessed	NR	No
S0024	C0026	PDD-NOS; ADHD	NR	Yes (for behavior; stabilized)
S0024	C0027	not assessed	NR	No
S0026	C0028	not assessed	NR	No
S0026	C0029	not assessed	NR	No
S0026	C0030	not assessed	NR	No
S0028	C0031	Conduct problems	screened	No
S0028	C0032	Conduct problems	screened	No
S0028	C0033	Conduct problems	screened	No
S0028	C0034	None	screened	No
S0028	C0035	Conduct problems	screened	No
S0028	C0036	None	screened	No
S0037	C0039	mixed behavior disorder of childhood with aggressive and hyperkinetic features	NR	No

S0037	C0041	mixed behavior disorder of childhood with aggressive features	NR	No
S0037	C0042	mixed behavior disorder of childhood with aggressive features and depressive features	NR	No
S0040	C0044	Conduct disorder	screened	No
S0047	C0045	None	screened	No
S0047	C0046	None	screened	No
S0047	C0047	None	screened	No
S0048	C0048	not assessed	NR	No
S0048	C0049	not assessed	NR	No
S0048	C0050	not assessed	NR	No
S0048	C0051	not assessed	NR	No
S0048	C0052	not assessed	NR	No
S0048	C0053	not assessed	NR	No
S0048	C0054	not assessed	NR	No
S0048	C0055	not assessed	NR	No
S0048	C0056	not assessed	NR	No
S0048	C0057	not assessed	NR	No
S0048	C0058	not assessed	NR	No
S0048	C0059	not assessed	NR	No
S0050	C0060	not assessed	NR	No
S0050	C0061	not assessed	NR	No
S0050	C0062	not assessed	NR	No
S0051	C0063	externalizing and hyperactivity behavior	screened	No
S0062	C0064	ODD; ADD	NR	Yes (ADHD)
S0062	C0065	ODD	NR	No
S0062	C0066	ODD; ADHD	NR	Yes (ADHD)
S0070	C0068	ODD; ADHD	screened	No
S0070	C0069	ODD; ADHD	screened	No
S0070	C0070	ODD; ADHD	screened	No
S0071	C0071	ODD; ADHD	screened	retalin
S0071	C0072	ODD; ADHD	screened	No
S0071	C0073	ODD; ADHD	screened	No

S0071	C0074	ODD; ADHD	screened	No
S0071	C0075	ODD; ADHD	screened	retalin
S0071	C0076	ODD; ADHD	screened	No
S0071	C0077	ODD; ADHD	screened	No
S0071	C0078	ODD; ADHD	screened	retalin
S0075	C0091	not assessed	NR	No
S0084	C0092	not assessed	NR	No
S0084	C0093	not assessed	NR	No
S0096	C0100	emotional and behavioral disorders	screened	No
S0096	C0101	Conduct disorder	screened	No
S0096	C0102	None	screened	No
S0096	C0103	emotional and behavioral disorders	screened	No
S0096	C0104	emotional and behavioral disorders	screened	No
S0096	C0105	emotional and behavioral disorders	screened	No
S0101	C0106	ODD	screened	No
S0108	C0107	None	screened	No
S0113	C0109	not assessed	NR	No
S0113	C0110	not assessed	NR	No
S0113	C0111	not assessed	NR	No
S0113	C0112	not assessed	NR	No
S0113	C0113	not assessed	NR	No
S0113	C0114	not assessed	NR	No
S0116	C0115	ODD	diagnostic interview	No
S0120	C0116	ADHD	NR	Methylphenidate + Clonidine
S0120	C0117	not assessed	NR	No
S0120	C0118	not assessed	NR	No
S0120	C0119	not assessed	NR	No
S0122	C0120	not assessed	NR	No
S0122	C0121	not assessed	NR	No
S0122	C0122	not assessed	NR	No

S0123	C0126	not assessed	NR	No
S0123	C0128	not assessed	NR	No
S0123	C0131	not assessed	NR	No
S0123	C0132	not assessed	NR	No
S0124	C0142	internalizing behavior	screened	No
S0124	C0143	externalizing behavior	screened	No
S0125	C0145	severe behavior disorders (SBD)	NR	No
S0129	C0146	not assessed	NR	No
S0129	C0147	not assessed	NR	No
S0129	C0148	not assessed	NR	No
S0129	C0149	not assessed	NR	No
S0129	C0150	not assessed	NR	No
S0129	C0151	not assessed	NR	No
S0129	C0152	not assessed	NR	No
S0129	C0153	not assessed	NR	No
S0129	C0154	not assessed	NR	No
S0129	C0155	not assessed	NR	No
S0129	C0156	not assessed	NR	No
S0134	C0157	expressive and receptive language delays	screened	No
S0134	C0158	developmental delays and disruptive behavior disorder	screened	No
S0136	C0160	not assessed	NR	No
S0136	C0161	Cornelia de Lange Syndrome	NR	No
S0136	C0162	not assessed	NR	No
S0138	C0163	developmental delay and speech language delay	screened	No
S0138	C0164	ASD; speech language delay	screened	No
S0138	C0165	ASD; speech language delay	screened	No
S0147	C0323	ADHD; ODD	screened	No
S0147	C0324	ADHD; ODD	screened	No
S0153	C0166	ADHD	NR	No
S0153	C0167	not assessed	NR	methylphenidate
S0153	C0168	ADHD	NR	methylphenidate and risperidone

S0154	C0169	ADHD	NR	No
S0154	C0170	ADHD	NR	No
S0154	C0171	not assessed	NR	No
S0154	C0172	not assessed	NR	No
S0155	C0173	not assessed	NR	No
S0155	C0174	not assessed	NR	No
S0155	C0175	not assessed	NR	No
S0156	C0176	behavior disorders	screened	No
S0156	C0177	behavior disorders	screened	No
S0156	C0178	behavior disorders	screened	No
S0159	C0180	not assessed	NR	No
S0159	C0181	not assessed	NR	No
S0159	C0182	not assessed	NR	No
S0159	C0183	not assessed	NR	No
S0159	C0184	not assessed	NR	No
S0159	C0185	not assessed	NR	No
S0159	C0186	not assessed	NR	No
S0159	C0187	not assessed	NR	No
S0159	C0188	Oppositional defiant disorder, attention deficit disorder with hyperactivity	NR	No
S0159	C0189	not assessed	NR	No
S0159	C0190	not assessed	NR	No
S0165	C0191	ODD and ADHD	diagnostic interview	No
S0167	C0325	ASD	screened	No
S0170	C0192	ASD	NR	No
S0170	C0193	ASD	NR	No
S0170	C0194	ASD	NR	No
S0170	C0195	ASD	NR	No
S0173	C0196	ADHD	NR	No
S0173	C0197	Emotional Disturbance (ED)	NR	No
S0173	C0198	ADHD	NR	No
S0173	C0199	ADHD	NR	No

S0180	C0200	emotional and behavioral disorders	NR	No
S0180	C0201	emotional and behavioral disorders	NR	No
S0192	C0205	conduct disorder; undersocialized aggressive and attention deficit disorder without hyperactivity	screened	No
S0192	C0206	conduct disorder; undersocialized aggressive and attention deficit disorder with hyperactivity	screened	No
S0192	C0207	conduct disorder; undersocialized aggressive	screened	No
S0192	C0208	atypical depression and oppositional disorder	screened	No
S0197	C0211	Articulation and Language Disorders; Atthention Deficit/Hyperactivity Disorder; Pervasive Developmental Disorder	NR	No
S0197	C0212	Articulation and Language Disorder and Pervasive Developmental Disorder	NR	No
S0197	C0213	Articulation and Language Disorders; Atthention Deficit/Hyperactivity Disorder; Pervasive Developmental Disorder	NR	No
S0198	C0214	ASD	screened	No
S0198	C0215	ASD	screened	No
S0198	C0216	ASD	screened	No
S0202	C0217	ADHD	NR	Yes (ADHD)
S0202	C0218	not assessed	NR	No
S0202	C0219	not assessed	NR	No
S0207	C0220	not assessed	NR	No
S0207	C0221	not assessed	NR	No
S0207	C0222	not assessed	NR	No
S0207	C0224	not assessed	NR	No
S0214	C0225	ADHD	NR	Yes (ADHD)
S0214	C0226	Specidic learning disability in math	NR	No
S0214	C0227	Emotional Disturbance (ED)	NR	No
S0233	C0228	not assessed	NR	No
S0233	C0229	not assessed	NR	No
S0234	C0230	not assessed	NR	No
S0234	C0231	not assessed	NR	No
S0234	C0232	not assessed	NR	No
S0246	C0234	not assessed	NR	No
S0248	C0235	Conduct disorder	NR	No
S0248	C0236	Conduct disorder	NR	No

S0248	C0237	Conduct disorder	NR	No
S0248	C0238	Conduct disorder	NR	No
S0251	C0239	ASD	screened	No
S0251	C0240	ASD	screened	No
S0251	C0241	ASD	screened	No
S0253	C0243	not assessed	NR	No
S0253	C0244	not assessed	NR	No
S0253	C0245	not assessed	NR	No
S0255	C0246	not assessed	NR	No
S0255	C0247	not assessed	NR	No
S0255	C0248	not assessed	NR	No
S0255	C0249	not assessed	NR	No
S0258	C0250	not assessed	NR	No
S0258	C0251	not assessed	NR	No
S0258	C0252	not assessed	NR	No
S0258	C0253	not assessed	NR	No
S0262	C0255	not assessed	NR	No
S0262	C0256	not assessed	NR	No
S0262	C0257	not assessed	NR	No
S0262	C0258	not assessed	NR	No
S0262	C0259	not assessed	NR	No
S0267	C0263	ASD	NR	No
S0267	C0264	ASD	NR	No
S0267	C0265	ASD	NR	No
S0282	C0266	internalizing problems	screened	No
S0282	C0267	internalizing and externalzing behavior	screened	No
S0282	C0268	internalizing and externalzing behavior	screened	No
S0282	C0269	externalizing problems	screened	No
S0293	C0270	Asperger Syndrome	NR	No
S0293	C0271	Asperger Syndrome	NR	No
S0294	C0273	Conduct disorder	NR	No

S0294	C0274	Conduct disorder	NR	No
S0294	C0275	Conduct disorder	NR	No
S0296	C0276	ASD	NR	Risperidone
S0296	C0277	ASD	NR	Aripiprazole
S0296	C0278	ASD	NR	Risperidone
S0300	C0279	learning disabilities and concomitant behavioral problems	screened	No
S0300	C0280	learning disabilities and concomitant behavioral problems	screened	No
S0300	C0281	NR	NR	No
S0304	C0282	not assessed	NR	No
S0304	C0283	not assessed	NR	No
S0304	C0284	not assessed	NR	No
S0304	C0285	not assessed	NR	No
S0313	C0286	not assessed	NR	No
S0313	C0287	not assessed	NR	No
S0313	C0288	not assessed	NR	No
S0313	C0289	not assessed	NR	No
S0319	C352	not assessed	NR	No
S0329	C0291	not assessed	NR	No
S0329	C0292	not assessed	NR	No
S0329	C0293	not assessed	NR	No
S0329	C0294	not assessed	NR	No
S0329	C0295	not assessed	NR	No
S0329	C0296	not assessed	NR	No
S0329	C0297	not assessed	NR	No
S0329	C0298	not assessed	NR	No
S0329	C0299	not assessed	NR	No
S0331	C0300	Conduct disorder; undersocialized; aggressive; attention deficit disorder; residual type; borderline intellectual functioning	screened	No
S0336	C0301	ASD	screened	No
S0336	C0302	ASD	screened	No
S0336	C0303	ASD	screened	No
S0336	C0304	ASD	screened	No

S0336	C0305	ASD	screened	No
S0336	C0306	ASD	screened	No
S0341	C0307	ADHD; ODD	screened	No
S0342	C0308	not assessed	NR	No
S0342	C0309	not assessed	NR	No
S0345	C0310	not assessed	NR	No
S0345	C0311	not assessed	NR	No
S0345	C0312	not assessed	NR	No
S0345	C0313	not assessed	NR	No
S0345	C0314	not assessed	NR	No
S0345	C0315	not assessed	NR	No
S0358	C0316	not assessed	NR	No
S0359	C0317	not assessed	NR	No
S0359	C0318	not assessed	NR	No
S0359	C0319	not assessed	NR	No
S0359	C0320	not assessed	NR	No
S0359	C0321	not assessed	NR	No
S0359	C0322	not assessed	NR	No
S0362	C0341	at-risk EBD	screened	No
S0362	C0342	at-risk EBD	screened	No
S0362	C0343	at-risk EBD	screened	No
S0363	C0344	not assessed	NR	No
S0363	C0345	not assessed	NR	No
S0363	C0346	not assessed	NR	No
S0363	C0347	not assessed	NR	No
S0363	C0348	not assessed	NR	No
S0363	C0349	not assessed	NR	No

Supplement #3E – Child Characteristics Part 3

study_id	child_id	child_problem
S0005	C0001	Disruptive Behavior; Dysfunctional Jealousy; Rumination; Difficulty Managing Anger and Frustration
S0007	C0002	Assult; Externalizing behavior
S0007	C0003	Destroying Property; Externalizing behavior
S0007	C0004	Destroying Property; Externalizing behavior
S0007	C0005	Drugs; Externalizing Behavior
S0013	C0006	Disruptive Behavior
S0016	C0013	Noncompliance, Aggressive Behavior
S0016	C0014	Noncompliance, Aggressive Behavior
S0016	C0015	Noncompliance, Aggressive Behavior
S0017	C0016	Disruptive Behavior
S0017	C0017	Disruptive Behavior
S0017	C0018	Disruptive Behavior
S0017	C0019	Disruptive Behavior
S0017	C0020	Disruptive Behavior
S0017	C0021	Disruptive Behavior
S0024	C0025	compliance; socially inappropriate behavior (saying inappropriate things making noises, throwing things, poking other kids and blurting out); Off-task behavior
S0024	C0026	Noncompliance; self-control (has difficulties maintaing self-control with his mouth and body; engages in inappropriate behavior such as body flapping, shaking, making inappropriate noises, touching others, and blurting out)
S0024	C0027	Noncompliance; self-control (wanders around room; focused on distracting objects; makes inappropriate noises and verbalizations); socially inappropriate behavior (baby talk; poking other kids; blurting out; having melt-downs when things don't go his way or he's criticized); Lack of independednt Problem Solving (replies on adults to solve problems)
S0026	C0028	Aggression
S0026	C0029	Aggression
S0026	C0030	Aggression
S0028	C0031	Tantrum (e.g., crying, whining, breaking objects, stomping feet, pounding fists)
S0028	C0032	Leaving seat, speaking out, banging desk, inattention
S0028	C0033	Leaving seat, kicking chairs, pounding fists, destroying objects, inappropriate Language, speaking out, inattention
S0028	C0034	Tantrum (e.g., crying, yelling, throwing objects, task refusal), leaving seat, inattention
S0028	C0035	Speaking out, leaving seat, asking questions

S0028	C0036	Noncompliance, inappropriate language, inattention
S0037	C0039	violent outbursts when frustrated
S0037	C0041	aggressive and distructive at home and school; violent behavior
S0037	C0042	frequently provoked and teased her peers and was engaged in regular aggressive confrontations with other children in school
S0040	C0044	Noncompliance; destructivness; fire-lighting and facination with matches, electical wires and plugs; lack care; defiance; oppositional behavior
S0047	C0045	disruptive behavior including noncompliance; verbal aggression; and minor physical aggression
S0047	C0046	disruptive behavior including noncompliance; verbal aggression; and minor physical aggression
S0047	C0047	disruptive behavior including noncompliance; verbal aggression; and minor physical aggression
S0048	C0048	Noncompliance; disruption; out of seat; negative verbal and physical interaction
S0048	C0049	Noncompliance; disruption; out of seat; negative verbal and physical interaction
S0048	C0050	Noncompliance; disruption; out of seat; negative verbal and physical interaction
S0048	C0051	Noncompliance; disruption; out of seat; negative verbal and physical interaction
S0048	C0052	Noncompliance; disruption; out of seat; negative verbal and physical interaction
S0048	C0053	Noncompliance; disruption; out of seat; negative verbal and physical interaction
S0048	C0054	Noncompliance; disruption; out of seat; negative verbal and physical interaction
S0048	C0055	Noncompliance; disruption; out of seat; negative verbal and physical interaction
S0048	C0056	Noncompliance; disruption; out of seat; negative verbal and physical interaction
S0048	C0057	Noncompliance; disruption; out of seat; negative verbal and physical interaction
S0048	C0058	Noncompliance; disruption; out of seat; negative verbal and physical interaction
S0048	C0059	Noncompliance; disruption; out of seat; negative verbal and physical interaction
S0050	C0060	Off-task behavior; talking out; out-of-seat behavior; and noncompliance
S0050	C0061	Off-task behavior; talking out; out-of-seat behavior; and noncompliance
S0050	C0062	Off-task behavior; talking out; out-of-seat behavior; and noncompliance; and confrontational/ aggressive behavior
S0051	C0063	talking out, disruption, and noncompliance in the classroom
S0062	C0064	Argumentive and non-obedience; anger outbursts; poor social skills; collected and organized garbage and was resistant to throwing things away
S0062	C0065	Fighting; received negative reports from teachers weekly; talking back; throwing tantrums: throwing objects; getting angry; lying: and not listing to directions
S0062	C0066	does not follow directions; rude and oppsitional; difficulties interacting calmly with his parents; and acting out in general
S0070	C0068	Noncompliance; defiance; disruptive behavior; difficulties in following directions
S0070	C0069	Noncompliance; defiance; disruptive behavior; difficulties in following directions
S0070	C0070	Noncompliance; defiance; disruptive behavior; difficulties in following directions
S0071	C0071	Noncompliance

S0071	C0072	Noncompliance
S0071	C0073	Noncompliance
S0071	C0074	Noncompliance
S0071	C0075	Noncompliance
S0071	C0076	Noncompliance
S0071	C0077	Noncompliance
S0071	C0078	Noncompliance
S0075	C0091	running in and out of the room; cursing loudly at students and the teacher; refusing to do school work; stealing; and causing property damage; fighting and inappropriate responses to teacher control
S0084	C0092	swearing; yelling in class; making animal sounds; grunting; or whistling; aggressive behavior towards peers and teacher
S0084	C0093	swearing; yelling in class; making animal sounds; grunting; or whistling
S0096	C0100	Refusal to do work; non-compliance; inappropriate language; and walking out of class without permission
S0096	C0101	off-task behavior such as sleeping; putting head down; and non-academic talk
S0096	C0102	refusal to do work; sleeping: inappropriate language; non-academic talk; and wandering around the room
S0096	C0103	disruption; including provoking peers by talking or touching; and engaging inappropriate language
S0096	C0104	off-task behavior; including inappropriate language; non-academic talk; loud sighs; and eye-rolling
S0096	C0105	disruption; including arguing with others; non-academic talk; and making faces or gestures
S0101	C0106	Aggression; Defiance; Tantrums; Threatening Behavior; Challenging Authority
S0108	C0107	aggression; inflexibility: annoying others; disobedience; emotion dysregulation; restlessness and hyperactivity; running away
S0113	C0109	low engagement; disruptive behavior
S0113	C0110	low engagement; disruptive behavior
S0113	C0111	low engagement; disruptive behavior
S0113	C0112	low engagement; disruptive behavior
S0113	C0113	low engagement; disruptive behavior
S0113	C0114	low engagement; disruptive behavior
S0116	C0115	oppositional behavior; temper tantrums; physical aggression
S0120	C0116	Physical and verbal aggression; noncompliance; talking out in class; verbal aggression; and physical aggression; disruptive classroom behaviors; and there was a suspicion that some of the talking out in class was a verbal tic potentially due to his medication
S0120	C0117	Physical and verbal aggression; noncompliance; verbal class disruption; and physical aggression; social problems; attention problems; aggressive behavior; and anxious/depressed behaviors
S0120	C0118	Highly aggressive physical and verbal behavior; as noted by his mother
S0120	C0119	Physical and verbal aggression; noncompliance; withdrawn behavior; anxious/depressed behavior; aggressive behavior; and attention problems
S0122	C0120	Verbal aggression; physical aggression; oppositional behavior

S0122	C0121	Verbal aggression; physical aggression; oppositional behavior
S0122	C0122	Verbal aggression; physical aggression; oppositional behavior
S0123	C0126	Inappropriate language; rude or offensive language; name-calling; insults to siblings and other children.
S0123	C0128	Behavioral problems; hitting when angry; difficulty using words and talking out feelings
S0123	C0131	Aggression toward siblings; hitting and pushing
S0123	C0132	Frustration; outbursts; hitting; throwing; screaming
S0124	C0142	social isolation
S0124	C0143	extreme aggressive behavior
S0125	C0145	Making inappropriate noises; talking out without permission; walking away from instructional area without permission; non-directed negative verbaliziations
S0129	C0146	disruptive off-task behavior
S0129	C0147	disruptive off-task behavior
S0129	C0148	disruptive off-task behavior
S0129	C0149	disruptive off-task behavior
S0129	C0150	disruptive off-task behavior
S0129	C0151	disruptive off-task behavior
S0129	C0152	disruptive off-task behavior
S0129	C0153	disruptive off-task behavior
S0129	C0154	disruptive off-task behavior
S0129	C0155	disruptive off-task behavior
S0129	C0156	disruptive off-task behavior
S0134	C0157	Aggression (hitting, kicking); property destruction (throwing objects)
S0134	C0158	Aggression (hitting, kicking); property destruction (throwing objects)
S0136	C0160	Negative vocalization; running out of the room; self-injurious behavior; flopping to the floor
S0136	C0161	Negative vocalization; hitting head with hands; flopping to the floor
S0136	C0162	Getting up from the dining table and using hands to engage in activities other than consuming food
S0138	C0163	behavioral issues
S0138	C0164	behavioral issues
S0138	C0165	behavioral issues
S0147	C0323	disruptive and oppositional behavior
S0147	C0324	disruptive and oppositional behavior
S0153	C0166	Disruption; out of seat; noncompliance; negative verbal or physical interaction

S0153	C0167	Disruption; out of seat; noncompliance; negative verbal or physical interaction
S0153	C0168	Disruption; out of seat; noncompliance; negative verbal or physical interaction
S0154	C0169	Problems with concentration during lessons; particularly in English; and conflicts with classmates
S0154	C0170	Problems following adult instructions; tolerating failures; improper language; and talking during lessons
S0154	C0171	Difficulty obeying adult instructions; staying seated; and completing tasks during lessons
S0154	C0172	Problems waiting his turn; losing temper when instructed by adults; and using improper language
S0155	C0173	Irritability; initiating arguments; and refusing to speak with his mother
S0155	C0174	Verbal outbursts; yelling at parents; and accusing parents of being unfair
S0155	C0175	verbal aggression; arguing with family members; and using profanity
S0156	C0176	touching; vocalizing; aggression; playing; disorienting; making noise; out of seat
S0156	C0177	touching; vocalizing; aggression; playing; disorienting; making noise; out of seat
S0156	C0178	touching; vocalizing; aggression; playing; disorienting; making noise; out of seat
S0159	C0180	Inattentiveness, impulsivity, poor anger control, excessive resistance
S0159	C0181	Impulsivity, poor anger control, excessive resistance
S0159	C0182	Aggressive, disruptive, volatile behaviors
S0159	C0183	Acting out, hyperactivity, poor peer interactions
S0159	C0184	Poor peer interactions, aggression, poor impulse control, hyperactivity
S0159	C0185	Poor peer interactions, aggression, hyperactivity
S0159	C0186	Poor peer interactions, aggression, disruption
S0159	C0187	Poor peer interactions, short attention span, tantrums
S0159	C0188	Poor peer interactions, fighting
S0159	C0189	Poor social interactions
S0159	C0190	Tantrums at school
S0165	C0191	severe aggression toward people and animals; bullying; threatening others; starting fights; deliberately hurting others; property destruction; theft; oppositional behavior; defiance; argumentativeness; hyperactivity; and chronic attempts to gain negative attention
S0167	C0325	impaired social communication and interaction as well as restricted, repetitive patterns of behaviors, interests, or activities
S0170	C0192	Maladaptive Behaviors such as screaming, hitting, kicking, pushing, biting, pinching, scratiching throwing objects, and engaging in self-simulatory behaviors; social deficity such as lack of verbal social initations and difficulties with perspective-taking
S0170	C0193	Maladaptive Behaviors such as screaming, hitting, kicking, pushing, biting, pinching, scratiching throwing objects, and engaging in self-simulatory behaviors; social deficity such as lack of verbal social initations and difficulties with perspective-taking
S0170	C0194	Maladaptive Behaviors such as screaming, hitting, kicking, pushing, biting, pinching, scratiching throwing objects, and engaging in self-simulatory behaviors; social deficity such as lack of verbal social initations and difficulties with perspective-taking
S0170	C0195	Maladaptive Behaviors such as screaming, hitting, kicking, pushing, biting, pinching, scratiching throwing objects, and engaging in self-simulatory behaviors; social deficity such as lack of verbal social initations and difficulties with perspective-taking
S0173	C0196	Off-task behavior; disrespectful behavior towards teachers; and low academic engagement

S0173	C0197	Inconsistent academic engagement; noncompliance with teacher instructions; and verbal aggression
S0173	C0198	Disruptive behavior; lack of focus; and poor academic engagement
S0173	C0199	Frequent outbursts; disrespectful interactions; and lack of academic focus
S0180	C0200	Denial behavior in response to questions or statements made by a teacher, aide, or peer; Talking back in response to commands given by an adult in the classroom
S0180	C0201	Talking back in response to commands given by an adult in the classroom; verbal reactions to peers during times when the participant was supposed to be quietly engaged in individual seatwork
S0192	C0205	conduct disorder; undersocialized aggressive and attention deficit disorder without hyperactivity
S0192	C0206	conduct disorder; undersocialized aggressive and attention deficit disorder with hyperactivity
S0192	C0207	conduct disorder; undersocialized aggressive
S0192	C0208	atypical depression and oppositional disorder
S0197	C0211	behavior problems and non-compliance
S0197	C0212	behavior problems and non-compliance
S0197	C0213	behavior problems and non-compliance
S0198	C0214	Physical aggression toward parents and peers (e.g., hitting, kicking, biting); Consistent noncompliance to requests at home and school; Difficulty focusing for long periods of time; High levels of activity throughout the day, more frequent and intense than similar-aged children; Frequent aggression leading to difficulties establishing relationships and experiencing peer rejection
S0198	C0215	Argumentative and defiant behavior toward requests from his mother and school staff; Noncompliance, often requiring threats of punishment to obey; Engaged in risky behaviors, such as running away from adults in public places; Aggression toward siblings and peers (e.g., throwing objects at others); Easily distracted, difficulty sustaining attention, frequent interruptions, and constant attention-seeking behavior; Occasionally destroyed toys or objects; Frequent off-task and noncompliant behavior during baseline sessions
S0198	C0216	Defiant behavior and noncompliance with demands at home, school, and with interventionists; Refusal to eat food presented to him; Frequent crying and yelling; Physical aggression toward parents and objects (e.g., hitting the wall); Frequently off-task, engaging in activities of his preference rather than following instructions
S0202	C0217	Defiance of authority; anger and aggression; easy frustration with difficult academic tasks; inability to attend to instruction or sit still
S0202	C0218	Easy aggravation; poor conflict resolution; poor self-control during difficult social interactions or academic demands
S0202	C0219	Difficulty with authority; self-control issues; difficulties in relationships with adults; aggressive outbursts
S0207	C0220	Physical aggression (e.g. hitting, biting, poking, spitting); verbal aggression (e.g. swearing; arguing; teasing; shouting); severe disruptive confrontations (e.g. fighting)
S0207	C0221	Physical aggression (e.g. hitting, biting, poking, spitting); verbal aggression (e.g. swearing; arguing; teasing; shouting); severe disruptive confrontations (e.g. fighting)
S0207	C0222	Physical aggression (e.g. hitting, biting, poking, spitting); verbal aggression (e.g. swearing; arguing; teasing; shouting); severe disruptive confrontations (e.g. fighting)
S0207	C0224	Physical aggression (e.g. hitting, biting, poking, spitting); verbal aggression (e.g. swearing; arguing; teasing; shouting); severe disruptive confrontations (e.g. fighting)
S0214	C0225	Frequent prompts and redirections needed to begin and complete work; difficulty interacting with peers; mumbling to himself following directions; high and variable disruptive behavior
S0214	C0226	Low levels of compliance to academic and behavioral directions; refusal to complete work; struggles in small group discussions
S0214	C0227	Frequent disruptive behavior during math class; walking away from instructional areas; making disruptive noises; leaving the classroom without permission; difficulty managing frustration

S0233	C0228	Aggressive behaviors such as pushing, kicking, head butting, pulling, and throwing objects at peers in the schoolyard
S0233	C0229	Aggressive behaviors such as pushing, kicking, head butting, pulling, and throwing objects at peers in the schoolyard
S0234	C0230	Talking without permission; Talking back to others; Being inappropriately out of his seat
S0234	C0231	Not following directions; Aggression; Off-task behavior
S0234	C0232	Verbal aggression; Physical aggression; Off-task behavior
S0246	C0234	anger outbursts at home; screaming and sometimes kicking or hitting younger siblings
S0248	C0235	Out-of-seat behavior; touching others; vocalizations; playing; disorienting; making noise; aggression
S0248	C0236	Out-of-seat behavior; touching others; vocalizations; playing; disorienting; making noise; aggression
S0248	C0237	Out-of-seat behavior; touching others; vocalizations; playing; disorienting; making noise; aggression
S0248	C0238	Out-of-seat behavior; touching others; vocalizations; playing; disorienting; making noise; aggression
S0251	C0239	Hitting; throwing objects; grabbing others; screaming; and crying/whimpering
S0251	C0240	Being off-task; verbal protesting; screaming; whining; and kicking
S0251	C0241	negative commenting; whining; and spitting out food
S0253	C0243	low rates of cooperative behavior; low rates of proximity to peers; various disruptive and oppositional behaviors
S0253	C0244	low rates of cooperative behavior; low rates of proximity to peers; various disruptive and oppositional behaviors
S0253	C0245	low rates of cooperative behavior; low rates of proximity to peers; various disruptive and oppositional behaviors
S0255	C0246	biting; lack of sharing; destruction of other children's projects
S0255	C0247	aggressive behavior; inability to interact effectively with peers
S0255	C0248	difficulties sharing; destructuve with materials (i.e. throwing toys and knocking over structures made by peers)
S0255	C0249	lack of sharing; social isolation; tantrum behavior; throwing materials; hitting; and screaming
S0258	C0250	disruptive behavior; confrontations with peers
S0258	C0251	disruptive behavior; confrontations with peers
S0258	C0252	disruptive behavior; confrontations with peers
S0258	C0253	disruptive behavior; confrontations with peers
S0262	C0255	Disruption; Disrespect
S0262	C0256	Teasing; physical aggression
S0262	C0257	Teasing; Gossip
S0262	C0258	Teasing; Gossip
S0262	C0259	Non-compliance; physical aggression
S0267	C0263	Hitting peers and staff; Kicking peers and staff; Biting; Hair-pulling; Engaging in self-injurious behaviors such as hitting his own head
S0267	C0264	Hitting peers and staff; Kicking peers and staff; Biting; Hair-pulling; Engaging in self-injurious behaviors such as hitting his own head

S0267	C0265	Hitting peers and staff; Kicking peers and staff; Biting; Hair-pulling; Engaging in self-injurious behaviors such as hitting his own head
S0282	C0266	anxious/depressed; withdrawn/depressed; somatic complaints; aggressive behavior
S0282	C0267	anxious/depressed; social problems; attention problems; rule-breaking behavior; aggressive behavior
S0282	C0268	anxious/depressed; thought problems; rule-breaking behavior
S0282	C0269	anxious/ depressed; social problems; thought problems; rule-breaking behavior
S0293	C0270	Hitting; kicking; biting; scratching
S0293	C0271	Hitting; kicking; slapping; scratching; destroying property
S0294	C0273	bullying; fire setting
S0294	C0274	Aggression; cruelty to animals
S0294	C0275	Aggresion; non-compliance
S0296	C0276	verbal and physical aggression (i.e. kicking; hitting with closed fist; biting)
S0296	C0277	verbal and physical aggression (i.e. slapping and punching family members)
S0296	C0278	verbal and physical aggression (i.e. kicking and hitting)
S0300	C0279	Teasing; hitting; kicking desks; pushing chairs; throwing books; and shouting
S0300	C0280	Fighting with other students; throwing books; refusing to cooperate; and making disruptive noises
S0300	C0281	Blaming others; physical intimation; property destruction; and shouting obscenties
S0304	C0282	maladaptive behaviors and aggression
S0304	C0283	maladaptive behaviors and aggression
S0304	C0284	maladaptive behaviors and aggression
S0304	C0285	maladaptive behaviors and aggression
S0313	C0286	displaying maladaptive and aggressive behaviors
S0313	C0287	displaying maladaptive and aggressive behaviors
S0313	C0288	displaying maladaptive and aggressive behaviors
S0313	C0289	displaying maladaptive and aggressive behaviors
S0319	C352	outburts of temper, anger, impulsive fighting, hitting and swearing
S0329	C0291	off-task verbal behavior (i.e. laughing; hummin; singing; yelling; crying; engaging in conversation with peers; or engaging in clowing behavior); off-task physical behavior (i.e. deviats from the assigned task; such as playing out of turn; shouting; throwing objects; disruptiing the flow of the activity; using sports equipment inappropriately; interrupting practice; leaving the practice area without permission; diregarding rules); exhibiting aggressive behavior
S0329	C0292	off-task verbal behavior (i.e. laughing; hummin; singing; yelling; crying; engaging in conversation with peers; or engaging in clowing behavior); off-task physical behavior (i.e. deviats from the assigned task; such as playing out of turn; shouting; throwing objects; disruptiing the flow of the activity; using sports equipment inappropriately; interrupting practice; leaving the practice area without permission; diregarding rules); exhibiting aggressive behavior
S0329	C0293	off-task verbal behavior (i.e. laughing; hummin; singing; yelling; crying; engaging in conversation with peers; or engaging in clowing behavior); off-task physical behavior (i.e. deviats from the assigned task; such as playing out of turn; shouting; throwing objects; disruptiing the flow of the activity; using sports equipment inappropriately; interrupting practice; leaving the practice area without permission; diregarding rules); exhibiting aggressive behavior

S0329	C0294	off-task verbal behavior (i.e. laughing; hummin; singing; yelling; crying; engaging in conversation with peers; or engaging in clowing behavior); off-task physical behavior (i.e. deviats from the assigned task; such as playing out of turn; shouting; throwing objects; disrupting the flow of the activity; using sports equipment inappropriately; interrupting practice; leaving the practice area without permission; diregarding rules); exhibiting aggressive behavior
S0329	C0295	off-task verbal behavior (i.e. laughing; hummin; singing; yelling; crying; engaging in conversation with peers; or engaging in clowing behavior); off-task physical behavior (i.e. deviats from the assigned task; such as playing out of turn; shouting; throwing objects; disruptiing the flow of the activity; using sports equipment inappropriately; interrupting practice; leaving the practice area without permission; diregarding rules); exhibiting aggressive behavior
S0329	C0296	off-task verbal behavior (i.e. laughing; hummin; singing; yelling; crying; engaging in conversation with peers; or engaging in clowing behavior); off-task physical behavior (i.e. deviats from the assigned task; such as playing out of turn; shouting; throwing objects; disruptiing the flow of the activity; using sports equipment inappropriately; interrupting practice; leaving the practice area without permission; diregarding rules); exhibiting aggressive behavior
S0329	C0297	off-task verbal behavior (i.e. laughing; hummin; singing; yelling; crying; engaging in conversation with peers; or engaging in clowing behavior); off-task physical behavior (i.e. deviats from the assigned task; such as playing out of turn; shouting; throwing objects; disruptiing the flow of the activity; using sports equipment inappropriately; interrupting practice; leaving the practice area without permission; diregarding rules); exhibiting aggressive behavior
S0329	C0298	off-task verbal behavior (i.e. laughing; hummin; singing; yelling; crying; engaging in conversation with peers; or engaging in clowing behavior); off-task physical behavior (i.e. deviats from the assigned task; such as playing out of turn; shouting; throwing objects; disruptiing the flow of the activity; using sports equipment inappropriately; interrupting practice; leaving the practice area without permission; diregarding rules); exhibiting aggressive behavior
S0329	C0299	off-task verbal behavior (i.e. laughing; hummin; singing; yelling; crying; engaging in conversation with peers; or engaging in clowing behavior); off-task physical behavior (i.e. deviats from the assigned task; such as playing out of turn; shouting; throwing objects; disruptiing the flow of the activity; using sports equipment inappropriately; interrupting practice; leaving the practice area without permission; diregarding rules); exhibiting aggressive behavior
S0331	C0300	aggressive behavior; sucidal threats and gestures; poor social interavtions with peers and somatic complaints (headaches and stomach-aches)
S0336	C0301	Aggression; self-injury; property destruction; screaming; elopement; repetive behavior; dangerous behavior; task refusal
S0336	C0302	Aggression; self-injury; property destruction; screaming; elopement; repetive behavior; dangerous behavior; task refusal
S0336	C0303	Aggression; self-injury; property destruction; screaming; elopement; repetive behavior; dangerous behavior; task refusal
S0336	C0304	Aggression; self-injury; property destruction; screaming; elopement; repetive behavior; dangerous behavior; task refusal
S0336	C0305	Aggression; self-injury; property destruction; screaming; elopement; repetive behavior; dangerous behavior; task refusal
S0336	C0306	Aggression; self-injury; property destruction; screaming; elopement; repetive behavior; dangerous behavior; task refusal
S0341	C0307	interruptions; rule violations during classroom activities; teasing peers
S0342	C0308	continuing one activity when the class had been given directions for another; throwing a basketball at the ceiling; pulling workbooks away from other children; and running around the room and out into the hall; also displayed verbal and physical aggressiveness toward other children
S0342	C0309	responded to teachers' directions with defiance; struck and kicked the teacher when pressed for appropriate behavior; engaged in behaviors that were disruptive to the class
S0345	C0310	verbal aggression (i.e. talking back, arguing, making derogatory and obscene comments); curfew compliance (i.e. ahering to agreed-upon curfew times); school attendance (i.e. regulary attenting school); performance of chores (i.e. completing assigned houshold tasks like cleaning their room, taking the trash, and doing yard work)
S0345	C0311	verbal aggression (i.e. talking back, arguing, making derogatory and obscene comments); curfew compliance (i.e. ahering to agreed-upon curfew times); school attendance (i.e. regulary attenting school); performance of chores (i.e. completing assigned houshold tasks like cleaning their room, taking the trash, and doing yard work)
S0345	C0312	verbal aggression (i.e. talking back, arguing, making derogatory and obscene comments); curfew compliance (i.e. ahering to agreed-upon curfew times); school attendance (i.e. regulary attenting school); performance of chores (i.e. completing assigned houshold tasks like cleaning their room, taking the trash, and doing yard work)
S0345	C0313	verbal aggression (i.e. talking back, arguing, making derogatory and obscene comments); curfew compliance (i.e. ahering to agreed-upon curfew times); school attendance (i.e. regulary attenting school); performance of chores (i.e. completing assigned houshold tasks like cleaning their room, taking the trash, and doing yard work)
S0345	C0314	verbal aggression (i.e. talking back, arguing, making derogatory and obscene comments); curfew compliance (i.e. ahering to agreed-upon curfew times); school attendance (i.e. regulary attenting school); performance of chores (i.e. completing assigned houshold tasks like cleaning their room, taking the trash, and doing yard work)

S0345	C0315	verbal aggression (i.e. talking back, arguing, making derogatory and obscene comments); curfew compliance (i.e. ahering to agreed-upon curfew times); school attendance (i.e. regulary attenting school); performance of chores (i.e. completing assigned houshold tasks like cleaning their room, taking the trash, and doing yard work)
S0358	C0316	screaming; fighting; disobeying, and bossing; frequently screaming at peers; directing their activities; and enforcing his demands with physical aggression such as punches; kicks; and slaps
S0359	C0317	defiance and non-compliance; disruptive behavior
S0359	C0318	defiance and non-compliance; disruptive behavior
S0359	C0319	defiance and non-compliance; disruptive behavior
S0359	C0320	defiance and non-compliance; disruptive behavior
S0359	C0321	defiance and non-compliance; disruptive behavior
S0359	C0322	defiance and non-compliance; disruptive behavior
S0362	C0341	at-risk of being identified as having an emotional/behavior disorder due to repeated behavioral indicence and nomination for Tier 2 behavioral support
S0362	C0342	at-risk of having an emotional/behavior disorder due to repeated behavioral incidence that involved verbal and physical aggression and were documented through multiple office discipline referrals. Elevated scores on hyperacticity and conduct
S0362	C0343	at-risk of being identified as having an emotional/behavior disorder due to repeated behavioral indicence
S0363	C0344	problem behavior
S0363	C0345	problem behavior
S0363	C0346	problem behavior
S0363	C0347	problem behavior
S0363	C0348	problem behavior
S0363	C0349	problem behavior

Supplement #3F – Child Characteristics Part 4

study_id	child_id	child_comorbid
S0005	C0001	NR
S0007	C0002	NR
S0007	C0003	NR
S0007	C0004	NR
S0007	C0005	NR
S0013	C0006	NR
S0016	C0013	ADHD
S0016	C0014	None
S0016	C0015	ADHD
S0017	C0016	speech and language delays
S0017	C0017	NR
S0017	C0018	NR
S0017	C0019	NR
S0017	C0020	NR
S0017	C0021	NR
S0024	C0025	NR
S0024	C0026	NR
S0024	C0027	NR
S0026	C0028	NR
S0026	C0029	NR
S0026	C0030	NR
S0028	C0031	NR
S0028	C0032	NR
S0028	C0033	NR
S0028	C0034	NR
S0028	C0035	NR
S0028	C0036	NR
S0037	C0039	NR

S0037	C0041	NR
S0037	C0042	NR
S0040	C0044	asthma and excema
S0047	C0045	NR
S0047	C0046	NR
S0047	C0047	NR
S0048	C0048	NR
S0048	C0049	NR
S0048	C0050	NR
S0048	C0051	NR
S0048	C0052	NR
S0048	C0053	NR
S0048	C0054	NR
S0048	C0055	NR
S0048	C0056	NR
S0048	C0057	NR
S0048	C0058	NR
S0048	C0059	NR
S0050	C0060	NR
S0050	C0061	NR
S0050	C0062	NR
S0051	C0063	NR
S0062	C0064	ADD
S0062	C0065	None
S0062	C0066	ADHD
S0070	C0068	ADHD
S0070	C0069	ADHD
S0070	C0070	ADHD
S0071	C0071	ADHD
S0071	C0072	ADHD
S0071	C0073	ADHD

S0071	C0074	ADHD
S0071	C0075	ADHD
S0071	C0076	ADHD
S0071	C0077	ADHD
S0071	C0078	ADHD
S0075	C0091	NR
S0084	C0092	internalizing problems
S0084	C0093	NA
S0096	C0100	None
S0096	C0101	None
S0096	C0102	None
S0096	C0103	None
S0096	C0104	other health impairment
S0096	C0105	None
S0101	C0106	NR
S0108	C0107	NR
S0113	C0109	NR
S0113	C0110	NR
S0113	C0111	NR
S0113	C0112	NR
S0113	C0113	NR
S0113	C0114	NR
S0116	C0115	NR
S0120	C0116	school suspects learning disability
S0120	C0117	school suspects learning disability
S0120	C0118	NR
S0120	C0119	NR
S0122	C0120	NR
S0122	C0121	NR
S0122	C0122	NR
S0123	C0126	NR

S0123	C0128	NR
S0123	C0131	NR
S0123	C0132	NR
S0124	C0142	externalizing behavior
S0124	C0143	internalizing behavior
S0125	C0145	NR
S0129	C0146	NR
S0129	C0147	NR
S0129	C0148	NR
S0129	C0149	NR
S0129	C0150	NR
S0129	C0151	NR
S0129	C0152	NR
S0129	C0153	NR
S0129	C0154	NR
S0129	C0155	NR
S0129	C0156	NR
S0134	C0157	NR
S0134	C0158	NR
S0136	C0160	NR
S0136	C0161	NR
S0136	C0162	NR
S0138	C0163	NR
S0138	C0164	NR
S0138	C0165	NR
S0147	C0323	ODD
S0147	C0324	ODD
S0153	C0166	NR
S0153	C0167	NR
S0153	C0168	NR
S0154	C0169	NR

S0154	C0170	NR
S0154	C0171	NR
S0154	C0172	NR
S0155	C0173	NR
S0155	C0174	NR
S0155	C0175	NR
S0156	C0176	NR
S0156	C0177	NR
S0156	C0178	NR
S0159	C0180	NR
S0159	C0181	NR
S0159	C0182	NR
S0159	C0183	NR
S0159	C0184	NR
S0159	C0185	NR
S0159	C0186	NR
S0159	C0187	NR
S0159	C0188	NR
S0159	C0189	NR
S0159	C0190	NR
S0165	C0191	CD and sleep disorder
S0167	C0325	NR
S0170	C0192	NR
S0170	C0193	NR
S0170	C0194	NR
S0170	C0195	NR
S0173	C0196	NR
S0173	C0197	NR
S0173	C0198	NR
S0173	C0199	NR
S0180	C0200	NR

S0180	C0201	NR
S0192	C0205	mixed specific developmental disorder
S0192	C0206	developmental reading disorder
S0192	C0207	None
S0192	C0208	None
S0197	C0211	NR
S0197	C0212	NR
S0197	C0213	NR
S0198	C0214	NR
S0198	C0215	NR
S0198	C0216	NR
S0202	C0217	NR
S0202	C0218	NR
S0202	C0219	NR
S0207	C0220	NR
S0207	C0221	NR
S0207	C0222	NR
S0207	C0224	NR
S0214	C0225	ADHD
S0214	C0226	NR
S0214	C0227	NR
S0233	C0228	NR
S0233	C0229	NR
S0234	C0230	NR
S0234	C0231	NR
S0234	C0232	NR
S0246	C0234	NR
S0248	C0235	NR
S0248	C0236	NR
S0248	C0237	NR
S0248	C0238	NR

S0251	C0239	NR
S0251	C0240	NR
S0251	C0241	NR
S0253	C0243	NR
S0253	C0244	NR
S0253	C0245	NR
S0255	C0246	NR
S0255	C0247	NR
S0255	C0248	NR
S0255	C0249	NR
S0258	C0250	NR
S0258	C0251	NR
S0258	C0252	NR
S0258	C0253	NR
S0262	C0255	NR
S0262	C0256	NR
S0262	C0257	NR
S0262	C0258	NR
S0262	C0259	NR
S0267	C0263	NR
S0267	C0264	NR
S0267	C0265	NR
S0282	C0266	NR
S0282	C0267	NR
S0282	C0268	NR
S0282	C0269	NR
S0293	C0270	NR
S0293	C0271	NR
S0294	C0273	Learning disabilities
S0294	C0274	None
S0294	C0275	None

S0296	C0276	NR
S0296	C0277	NR
S0296	C0278	NR
S0300	C0279	NR
S0300	C0280	NR
S0300	C0281	NR
S0304	C0282	NR
S0304	C0283	NR
S0304	C0284	NR
S0304	C0285	NR
S0313	C0286	NR
S0313	C0287	speech problems
S0313	C0288	speech problems
S0313	C0289	speech problems
S0319	C352	NR
S0329	C0291	NR
S0329	C0292	NR
S0329	C0293	NR
S0329	C0294	NR
S0329	C0295	NR
S0329	C0296	NR
S0329	C0297	NR
S0329	C0298	NR
S0329	C0299	NR
S0331	C0300	developmental articulation disorder; abnormal EEG with normal neurological examination and CT scan; history of parental abuse and neglect
S0336	C0301	NR
S0336	C0302	NR
S0336	C0303	NR
S0336	C0304	NR
S0336	C0305	NR
S0336	C0306	NR

S0341	C0307	Callous-Unemotional traits
S0342	C0308	NR
S0342	C0309	NR
S0345	C0310	NR
S0345	C0311	NR
S0345	C0312	NR
S0345	C0313	NR
S0345	C0314	NR
S0345	C0315	NR
S0358	C0316	NR
S0359	C0317	NR
S0359	C0318	NR
S0359	C0319	NR
S0359	C0320	NR
S0359	C0321	NR
S0359	C0322	NR
S0362	C0341	NR
S0362	C0342	NR
S0362	C0343	NR
S0363	C0344	NR
S0363	C0345	NR
S0363	C0346	Autism
S0363	C0347	NR
S0363	C0348	Autism
S0363	C0349	NR

Supplement #3G – Child Characteristics Part 5

study_id	child_id	comorbid_problems
S0005	C0001	NR
S0007	C0002	NR
S0007	C0003	NR
S0007	C0004	NR
S0007	C0005	NR
S0013	C0006	NR
S0016	C0013	Impulsivity, Hyperactivity
S0016	C0014	none
S0016	C0015	Impulsivity, Hyperactivity
S0017	C0016	NR
S0017	C0017	NR
S0017	C0018	NR
S0017	C0019	NR
S0017	C0020	NR
S0017	C0021	NR
S0024	C0025	NR
S0024	C0026	NR
S0024	C0027	NR
S0026	C0028	NR
S0026	C0029	NR
S0026	C0030	NR
S0028	C0031	NR
S0028	C0032	NR
S0028	C0033	NR
S0028	C0034	NR
S0028	C0035	NR
S0028	C0036	NR
S0037	C0039	NR

S0037	C0041	NR
S0037	C0042	NR
S0040	C0044	none
S0047	C0045	NR
S0047	C0046	NR
S0047	C0047	NR
S0048	C0048	NR
S0048	C0049	NR
S0048	C0050	NR
S0048	C0051	NR
S0048	C0052	NR
S0048	C0053	NR
S0048	C0054	NR
S0048	C0055	NR
S0048	C0056	NR
S0048	C0057	NR
S0048	C0058	NR
S0048	C0059	NR
S0050	C0060	NR
S0050	C0061	NR
S0050	C0062	NR
S0051	C0063	NR
S0062	C0064	NR
S0062	C0065	NR
S0062	C0066	NR
S0070	C0068	NR
S0070	C0069	NR
S0070	C0070	NR
S0071	C0071	NR
S0071	C0072	NR
S0071	C0073	NR

S0071	C0074	NR
S0071	C0075	NR
S0071	C0076	NR
S0071	C0077	NR
S0071	C0078	NR
S0075	C0091	NR
S0084	C0092	negative self-statements
S0084	C0093	NA
S0096	C0100	NR
S0096	C0101	NR
S0096	C0102	NR
S0096	C0103	NR
S0096	C0104	NR
S0096	C0105	NR
S0101	C0106	NR
S0108	C0107	NR
S0113	C0109	NR
S0113	C0110	NR
S0113	C0111	NR
S0113	C0112	NR
S0113	C0113	NR
S0113	C0114	NR
S0116	C0115	NR
S0120	C0116	NR
S0120	C0117	NR
S0120	C0118	NR
S0120	C0119	NR
S0122	C0120	NR
S0122	C0121	NR
S0122	C0122	NR
S0123	C0126	NR

S0123	C0128	NR
S0123	C0131	NR
S0123	C0132	NR
S0124	C0142	NR
S0124	C0143	NR
S0125	C0145	NR
S0129	C0146	NR
S0129	C0147	NR
S0129	C0148	NR
S0129	C0149	NR
S0129	C0150	NR
S0129	C0151	NR
S0129	C0152	NR
S0129	C0153	NR
S0129	C0154	NR
S0129	C0155	NR
S0129	C0156	NR
S0134	C0157	NR
S0134	C0158	NR
S0136	C0160	NR
S0136	C0161	NR
S0136	C0162	NR
S0138	C0163	NR
S0138	C0164	NR
S0138	C0165	NR
S0147	C0323	defiance, irritability, and oppositional actions towards authority figures
S0147	C0324	defiance, irritability, and oppositional actions towards authority figures
S0153	C0166	NR
S0153	C0167	NR
S0153	C0168	NR
S0154	C0169	NR

S0154	C0170	NR
S0154	C0171	NR
S0154	C0172	NR
S0155	C0173	NR
S0155	C0174	NR
S0155	C0175	NR
S0156	C0176	NR
S0156	C0177	NR
S0156	C0178	NR
S0159	C0180	NR
S0159	C0181	NR
S0159	C0182	NR
S0159	C0183	NR
S0159	C0184	NR
S0159	C0185	NR
S0159	C0186	NR
S0159	C0187	NR
S0159	C0188	NR
S0159	C0189	NR
S0159	C0190	NR
S0165	C0191	executive functioning deficits; can't fall asleep at night; drowsiness during the day
S0167	C0325	NR
S0170	C0192	NR
S0170	C0193	NR
S0170	C0194	NR
S0170	C0195	NR
S0173	C0196	NR
S0173	C0197	NR
S0173	C0198	NR
S0173	C0199	NR
S0180	C0200	NR

S0180	C0201	NR
S0192	C0205	NR
S0192	C0206	NR
S0192	C0207	NR
S0192	C0208	NR
S0197	C0211	NR
S0197	C0212	NR
S0197	C0213	NR
S0198	C0214	NR
S0198	C0215	NR
S0198	C0216	NR
S0202	C0217	NR
S0202	C0218	NR
S0202	C0219	NR
S0207	C0220	NR
S0207	C0221	NR
S0207	C0222	NR
S0207	C0224	NR
S0214	C0225	NR
S0214	C0226	NR
S0214	C0227	NR
S0233	C0228	NR
S0233	C0229	NR
S0234	C0230	NR
S0234	C0231	NR
S0234	C0232	NR
S0246	C0234	NR
S0248	C0235	NR
S0248	C0236	NR
S0248	C0237	NR
S0248	C0238	NR

S0251	C0239	NR
S0251	C0240	NR
S0251	C0241	NR
S0253	C0243	NR
S0253	C0244	NR
S0253	C0245	NR
S0255	C0246	NR
S0255	C0247	NR
S0255	C0248	NR
S0255	C0249	NR
S0258	C0250	NR
S0258	C0251	NR
S0258	C0252	NR
S0258	C0253	NR
S0262	C0255	NR
S0262	C0256	NR
S0262	C0257	NR
S0262	C0258	NR
S0262	C0259	NR
S0267	C0263	NR
S0267	C0264	NR
S0267	C0265	NR
S0282	C0266	NR
S0282	C0267	NR
S0282	C0268	NR
S0282	C0269	NR
S0293	C0270	NR
S0293	C0271	NR
S0294	C0273	NR
S0294	C0274	NR
S0294	C0275	NR

S0296	C0276	NR
S0296	C0277	NR
S0296	C0278	NR
S0300	C0279	NR
S0300	C0280	NR
S0300	C0281	NR
S0304	C0282	NR
S0304	C0283	NR
S0304	C0284	NR
S0304	C0285	NR
S0313	C0286	NR
S0313	C0287	NR
S0313	C0288	NR
S0313	C0289	NR
S0319	C352	NR
S0329	C0291	NR
S0329	C0292	NR
S0329	C0293	NR
S0329	C0294	NR
S0329	C0295	NR
S0329	C0296	NR
S0329	C0297	NR
S0329	C0298	NR
S0329	C0299	NR
S0331	C0300	NR
S0336	C0301	NR
S0336	C0302	NR
S0336	C0303	NR
S0336	C0304	NR
S0336	C0305	NR
S0336	C0306	NR

S0341	C0307	NR
S0342	C0308	NR
S0342	C0309	NR
S0345	C0310	NR
S0345	C0311	NR
S0345	C0312	NR
S0345	C0313	NR
S0345	C0314	NR
S0345	C0315	NR
S0358	C0316	NR
S0359	C0317	NR
S0359	C0318	NR
S0359	C0319	NR
S0359	C0320	NR
S0359	C0321	NR
S0359	C0322	NR
S0362	C0341	NR
S0362	C0342	NR
S0362	C0343	NR
S0363	C0344	NR
S0363	C0345	NR
S0363	C0346	NR
S0363	C0347	NR
S0363	C0348	NR
S0363	C0349	NR

Supplement #3H – Child Characteristics Part 6

study_id	child_id	treatment_type	treatment_name
S0005	C0001	Mindfulness Therapy	Acceptance and Commitment Therapy (ACT)
S0007	C0002	Problem-Solving Therapy (PST)	Solution-focused therapy
S0007	C0003	Problem-Solving Therapy (PST)	Solution-focused therapy
S0007	C0004	Problem-Solving Therapy (PST)	Solution-focused therapy
S0007	C0005	Problem-Solving Therapy (PST)	Solution-focused therapy
S0013	C0006	Parent Behavior Training (PBT) + Mindfullness Therapy	Parent-Child Interaction Therapy & Acceptance and Commitment Therapy
S0016	C0013	Self-Monitoring (SM)	Video Self-Modeling (VSM)
S0016	C0014	Self-Monitoring (SM)	Video Self-Modeling (VSM)
S0016	C0015	Self-Monitoring (SM)	Video Self-Modeling (VSM)
S0017	C0016	Adapted Parent Behavior Training (PBT)	Adapted Parent Behavior Training (PBT)
S0017	C0017	Adapted Parent Behavior Training (PBT)	Adapted Parent Behavior Training (PBT)
S0017	C0018	Adapted Parent Behavior Training (PBT)	Adapted Parent Behavior Training (PBT)
S0017	C0019	Adapted Parent Behavior Training (PBT)	Adapted Parent Behavior Training (PBT)
S0017	C0020	Adapted Parent Behavior Training (PBT)	Adapted Parent Behavior Training (PBT)
S0017	C0021	Adapted Parent Behavior Training (PBT)	Adapted Parent Behavior Training (PBT)
S0024	C0025	Conjoint Behavioral	Conjoint Behavioral
S0024	C0026	Conjoint Behavioral	Conjoint Behavioral
S0024	C0027	Conjoint Behavioral	Conjoint Behavioral
S0026	C0028	Social Skills Training (SST)	Social stories (SS)
S0026	C0029	Social Skills Training (SST)	Social stories (SS)
S0026	C0030	Social Skills Training (SST)	Social stories (SS)
S0028	C0031	Problem-Solving Therapy (PST)	Teleconsultation: Problem-solving consultation
S0028	C0032	Problem-Solving Therapy (PST)	Teleconsultation: Problem-solving consultation
S0028	C0033	Problem-Solving Therapy (PST)	Teleconsultation: Problem-solving consultation
S0028	C0034	Problem-Solving Therapy (PST)	Teleconsultation: Problem-solving consultation
S0028	C0035	Problem-Solving Therapy (PST)	Teleconsultation: Problem-solving consultation
S0028	C0036	Problem-Solving Therapy (PST)	Teleconsultation: Problem-solving consultation

S0037	C0039	Social Skills Training (SST)	Social Skills Training (SST)
S0037	C0041	Social Skills Training (SST)	Social Skills Training (SST)
S0037	C0042	Social Skills Training (SST)	Social Skills Training (SST)
S0040	C0044	Parent Behavior Training (PBT)	Parent Behavior Training (PBT)
S0047	C0045	Behavior Modification Therapy (BMT)	Deferred Time-Out (DTO)
S0047	C0046	Behavior Modification Therapy (BMT)	Deferred Time-Out (DTO)
S0047	C0047	Behavior Modification Therapy (BMT)	Deferred Time-Out (DTO)
S0048	C0048	Behavior Modification Therapy (BMT) + Self Monitoring (SM)	Culturally Adapted Social and Emotional Learning (CA-SEL) Curriculum + Check-In/Check-Out (CICO) + Self-Monitoring with I-Connect:
S0048	C0049	Behavior Modification Therapy (BMT) + Self Monitoring (SM)	Culturally Adapted Social and Emotional Learning (CA-SEL) Curriculum + Check-In/Check-Out (CICO) + Self-Monitoring with I-Connect:
S0048	C0050	Behavior Modification Therapy (BMT) + Self Monitoring (SM)	Culturally Adapted Social and Emotional Learning (CA-SEL) Curriculum + Check-In/Check-Out (CICO) + Self-Monitoring with I-Connect:
S0048	C0051	Behavior Modification Therapy (BMT) + Self Monitoring (SM)	Culturally Adapted Social and Emotional Learning (CA-SEL) Curriculum + Check-In/Check-Out (CICO) + Self-Monitoring with I-Connect:
S0048	C0052	Behavior Modification Therapy (BMT) + Self Monitoring (SM)	Culturally Adapted Social and Emotional Learning (CA-SEL) Curriculum + Check-In/Check-Out (CICO) + Self-Monitoring with I-Connect:
S0048	C0053	Behavior Modification Therapy (BMT) + Self Monitoring (SM)	Culturally Adapted Social and Emotional Learning (CA-SEL) Curriculum + Check-In/Check-Out (CICO) + Self-Monitoring with I-Connect:
S0048	C0054	Behavior Modification Therapy (BMT) + Self Monitoring (SM)	Culturally Adapted Social and Emotional Learning (CA-SEL) Curriculum + Check-In/Check-Out (CICO) + Self-Monitoring with I-Connect:
S0048	C0055	Behavior Modification Therapy (BMT) + Self Monitoring (SM)	Culturally Adapted Social and Emotional Learning (CA-SEL) Curriculum + Check-In/Check-Out (CICO) + Self-Monitoring with I-Connect:
S0048	C0056	Behavior Modification Therapy (BMT) + Self Monitoring (SM)	Culturally Adapted Social and Emotional Learning (CA-SEL) Curriculum + Check-In/Check-Out (CICO) + Self-Monitoring with I-Connect:
S0048	C0057	Behavior Modification Therapy (BMT) + Self Monitoring (SM)	Culturally Adapted Social and Emotional Learning (CA-SEL) Curriculum + Check-In/Check-Out (CICO) + Self-Monitoring with I-Connect:
S0048	C0058	Behavior Modification Therapy (BMT) + Self Monitoring (SM)	Culturally Adapted Social and Emotional Learning (CA-SEL) Curriculum + Check-In/Check-Out (CICO) + Self-Monitoring with I-Connect:
S0048	C0059	Behavior Modification Therapy (BMT) + Self Monitoring (SM)	Culturally Adapted Social and Emotional Learning (CA-SEL) Curriculum + Check-In/Check-Out (CICO) + Self-Monitoring with I-Connect:
S0050	C0060	Behavior Modification Therapy (BMT)	First Step to Sucess
S0050	C0061	Behavior Modification Therapy (BMT)	First Step to Sucess
S0050	C0062	Behavior Modification Therapy (BMT)	First Step to Sucess
S0051	C0063	Behavior Modification Therapy (BMT)	First Step to Sucess
S0062	C0064	Problem-Solving Therapy (PST)	Solution-Focused Family Therapy (SFFT)
S0062	C0065	Problem-Solving Therapy (PST)	Solution-Focused Family Therapy (SFFT)
S0062	C0066	Problem-Solving Therapy (PST)	Solution-Focused Family Therapy (SFFT)
S0070	C0068	Behavior Modification Therapy (BMT)	Behavior Management Flow Chart (BMFC)
S0070	C0069	Behavior Modification Therapy (BMT)	Behavior Management Flow Chart (BMFC)

S0070	C0070	Behavior Modification Therapy (BMT)	Behavior Management Flow Chart (BMFC)
S0071	C0071	Behavior Modification Therapy (BMT)	Behavior Management Flow Chart (BMFC)
S0071	C0072	Behavior Modification Therapy (BMT)	Behavior Management Flow Chart (BMFC)
S0071	C0073	Behavior Modification Therapy (BMT)	Behavior Management Flow Chart (BMFC)
S0071	C0074	Behavior Modification Therapy (BMT)	Behavior Management Flow Chart (BMFC)
S0071	C0075	Behavior Modification Therapy (BMT)	Behavior Management Flow Chart (BMFC)
S0071	C0076	Behavior Modification Therapy (BMT)	Behavior Management Flow Chart (BMFC)
S0071	C0077	Behavior Modification Therapy (BMT)	Behavior Management Flow Chart (BMFC)
S0071	C0078	Behavior Modification Therapy (BMT)	Behavior Management Flow Chart (BMFC)
S0075	C0091	Behavior Modification Therapy (BMT)	Self-Observation via Video Tape
S0084	C0092	Self-Monitoring (SM)	Self-Management Training
S0084	C0093	Self-Monitoring (SM)	Self-Management Training
S0096	C0100	Behavior Modification Therapy (BMT)	Check-in, Check-out (CICO)
S0096	C0101	Behavior Modification Therapy (BMT)	Check-in, Check-out (CICO)
S0096	C0102	Behavior Modification Therapy (BMT)	Check-in, Check-out (CICO)
S0096	C0103	Behavior Modification Therapy (BMT)	Check-in, Check-out (CICO)
S0096	C0104	Behavior Modification Therapy (BMT)	Check-in, Check-out (CICO)
S0096	C0105	Behavior Modification Therapy (BMT)	Check-in, Check-out (CICO)
S0101	C0106	Parent Behavior Training (PBT)	Acceptance and Commitment Therapy (ACT) + Parent-Child Interaction Therapy (PCIT)
S0108	C0107	Parent Behavior Training (PBT)	Parent-Child Interaction Therapy (PCIT)
S0113	C0109	Behavior Modification Therapy (BMT)	Academic and Behavior Combined Support (ABC Support)
S0113	C0110	Behavior Modification Therapy (BMT)	Academic and Behavior Combined Support (ABC Support)
S0113	C0111	Behavior Modification Therapy (BMT)	Academic and Behavior Combined Support (ABC Support)
S0113	C0112	Behavior Modification Therapy (BMT)	Academic and Behavior Combined Support (ABC Support)
S0113	C0113	Behavior Modification Therapy (BMT)	Academic and Behavior Combined Support (ABC Support)
S0113	C0114	Behavior Modification Therapy (BMT)	Academic and Behavior Combined Support (ABC Support)
S0116	C0115	Parent Behavior Training (PBT)	Parent–Child Interaction Therapy (PCIT)
S0120	C0116	Parent Behavior Training (PBT)	home-based consultation
S0120	C0117	Parent Behavior Training (PBT)	home-based consultation
S0120	C0118	Parent Behavior Training (PBT)	home-based consultation
S0120	C0119	Parent Behavior Training (PBT)	home-based consultation

S0122	C0120	Parent Behavior Training (PBT)	Training of Mothers and Teachers
S0122	C0121	Parent Behavior Training (PBT)	Training of Mothers and Teachers
S0122	C0122	Parent Behavior Training (PBT)	Training of Mothers and Teachers
S0123	C0126	Social Skills Training (SST)	Social stories (SS)
S0123	C0128	Social Skills Training (SST)	Social stories (SS)
S0123	C0131	Social Skills Training (SST)	Social stories (SS)
S0123	C0132	Social Skills Training (SST)	Social stories (SS)
S0124	C0142	Self-Monitoring (SM)	self-monitoring procedures
S0124	C0143	Self-Monitoring (SM)	self-monitoring procedures
S0125	C0145	Behavior Modification Therapy (BMT)	Talk/Mand" procedure
S0129	C0146	Mindfulness Therapy	MindUP
S0129	C0147	Mindfulness Therapy	MindUP
S0129	C0148	Mindfulness Therapy	MindUP
S0129	C0149	Mindfulness Therapy	MindUP
S0129	C0150	Mindfulness Therapy	MindUP
S0129	C0151	Mindfulness Therapy	MindUP
S0129	C0152	Mindfulness Therapy	MindUP
S0129	C0153	Mindfulness Therapy	MindUP
S0129	C0154	Mindfulness Therapy	MindUP
S0129	C0155	Mindfulness Therapy	MindUP
S0129	C0156	Mindfulness Therapy	MindUP
S0134	C0157	Social Skills Training (SST)	Functional Communication Training (FCT)
S0134	C0158	Social Skills Training (SST)	Functional Communication Training (FCT)
S0136	C0160	Behavior Modification Therapy (BMT)	Prevent Teach Reinforce for Families (PTR-F)
S0136	C0161	Behavior Modification Therapy (BMT)	Prevent Teach Reinforce for Families (PTR-F)
S0136	C0162	Behavior Modification Therapy (BMT)	Prevent Teach Reinforce for Families (PTR-F)
S0138	C0163	Parent Behavior Training (PBT)	HOT DOCS
S0138	C0164	Parent Behavior Training (PBT)	HOT DOCS
S0138	C0165	Parent Behavior Training (PBT)	HOT DOCS
S0147	C0323	Parent-Child Interaction Therapy (PCIT)	Parent-Child Interaction Therapy (PCIT)
S0147	C0324	Parent-Child Interaction Therapy (PCIT)	Parent-Child Interaction Therapy (PCIT)

S0153	C0166	Behavior Modification Therapy (BMT)	Check-in, Check-out (CICO)
S0153	C0167	Behavior Modification Therapy (BMT)	Check-in, Check-out (CICO)
S0153	C0168	Behavior Modification Therapy (BMT)	Check-in, Check-out (CICO)
S0154	C0169	Behavior Modification Therapy (BMT)	Check-in, Check-out (CICO)
S0154	C0170	Behavior Modification Therapy (BMT)	Check-in, Check-out (CICO)
S0154	C0171	Behavior Modification Therapy (BMT)	Check-in, Check-out (CICO)
S0154	C0172	Behavior Modification Therapy (BMT)	Check-in, Check-out (CICO)
S0155	C0173	Cognitive-Behavioral Therapy (CBT)	COPE Teen program
S0155	C0174	Cognitive-Behavioral Therapy (CBT)	COPE Teen program
S0155	C0175	Cognitive-Behavioral Therapy (CBT)	COPE Teen program
S0156	C0176	Behavior Modification Therapy (BMT)	Self-Observation via Video Tape
S0156	C0177	Behavior Modification Therapy (BMT)	Self-Observation via Video Tape
S0156	C0178	Behavior Modification Therapy (BMT)	Self-Observation via Video Tape
S0159	C0180	Behavior Modification Therapy (BMT)	self-evaluation package
S0159	C0181	Behavior Modification Therapy (BMT)	self-evaluation package
S0159	C0182	Behavior Modification Therapy (BMT)	self-evaluation package
S0159	C0183	Behavior Modification Therapy (BMT)	self-evaluation package in group setting
S0159	C0184	Behavior Modification Therapy (BMT)	self-evaluation package in group setting
S0159	C0185	Behavior Modification Therapy (BMT)	self-evaluation package in group setting
S0159	C0186	Behavior Modification Therapy (BMT)	self-evaluation package in group setting
S0159	C0187	Behavior Modification Therapy (BMT)	self-evaluation package in group setting
S0159	C0188	Behavior Modification Therapy (BMT)	self-evaluation package in group setting
S0159	C0189	Behavior Modification Therapy (BMT)	self-evaluation package in group setting
S0159	C0190	Behavior Modification Therapy (BMT)	self-evaluation package in group setting
S0165	C0191	Parent Behavior Training (PBT)	Parent–Child Interaction Therapy (PCIT)
S0167	C0325	Parent-Child Interaction Therapy (PCIT)	Parent-Child Interaction Therapy (PCIT)
S0170	C0192	Social Skills Training (SST)	Social stories (SS)
S0170	C0193	Social Skills Training (SST)	Social stories (SS)
S0170	C0194	Social Skills Training (SST)	Social stories (SS)
S0170	C0195	Social Skills Training (SST)	Social stories (SS)
S0173	C0196	Self-Monitoring (SM)	Self-Monitoring with App

S0173	C0197	Self-Monitoring (SM)	Self-Monitoring with App
S0173	C0198	Self-Monitoring (SM)	Self-Monitoring with App
S0173	C0199	Self-Monitoring (SM)	Self-Monitoring with App
S0180	C0200	Behavior Modification Therapy (BMT)	Role-playing with reinforcement
S0180	C0201	Behavior Modification Therapy (BMT)	Role-playing with reinforcement
S0192	C0205	Behavior Management Skills Program (BMSP)	Stress Inoculation Therapy (SIT)
S0192	C0206	Behavior Management Skills Program (BMSP)	Stress Inoculation Therapy (SIT)
S0192	C0207	Behavior Management Skills Program (BMSP)	Stress Inoculation Therapy (SIT)
S0192	C0208	Behavior Management Skills Program (BMSP)	Stress Inoculation Therapy (SIT)
S0197	C0211	Behavior Modification Therapy (BMT)	Classroom-Based Time-In/Time-Out
S0197	C0212	Behavior Modification Therapy (BMT)	Classroom-Based Time-In/Time-Out
S0197	C0213	Behavior Modification Therapy (BMT)	Classroom-Based Time-In/Time-Out
S0198	C0214	Parent Behavior Training (PBT)	Parent-Child Interaction Therapy (PCIT)
S0198	C0215	Parent Behavior Training (PBT)	Parent-Child Interaction Therapy (PCIT)
S0198	C0216	Parent Behavior Training (PBT)	Parent-Child Interaction Therapy (PCIT)
S0202	C0217	Behavior Modification Therapy (BMT)	Coping Power (CP) + Check-in/Check-out (CICO)
S0202	C0218	Behavior Modification Therapy (BMT)	Coping Power (CP) + Check-in/Check-out (CICO)
S0202	C0219	Behavior Modification Therapy (BMT)	Coping Power (CP) + Check-in/Check-out (CICO)
S0207	C0220	Social Skills Training (SST)	Social Skills Training Instruction and Parental Involvment
S0207	C0221	Social Skills Training (SST)	Social Skills Training Instruction and Parental Involvment
S0207	C0222	Social Skills Training (SST)	Social Skills Training Instruction and Parental Involvment
S0207	C0224	Social Skills Training (SST)	Social Skills Training Instruction and Parental Involvment
S0214	C0225	Behavior Modification Therapy (BMT)	Good Behavior Game (GBG)
S0214	C0226	Behavior Modification Therapy (BMT)	Good Behavior Game (GBG)
S0214	C0227	Behavior Modification Therapy (BMT)	Good Behavior Game (GBG)
S0233	C0228	Self-Monitoring (SM)	video feedback and self-managment
S0233	C0229	Self-Monitoring (SM)	video feedback and self-managment
S0234	C0230	Conjoint Behavioral Consultation (CBC)	Conjoint Behavioral Consultation (CBC)
S0234	C0231	Conjoint Behavioral Consultation (CBC)	Conjoint Behavioral Consultation (CBC)
S0234	C0232	Conjoint Behavioral Consultation (CBC)	Conjoint Behavioral Consultation (CBC)
S0246	C0234	Haptotherapy	Haptotherapy for Children and Parents

S0248	C0235	Self-Monitoring (SM)	Self-Modeling (Videotaping)
S0248	C0236	Self-Monitoring (SM)	Self-Modeling (Videotaping)
S0248	C0237	Self-Monitoring (SM)	Self-Modeling (Videotaping)
S0248	C0238	Self-Monitoring (SM)	Self-Modeling (Videotaping)
S0251	C0239	Mindfulness Therapy	mindfulness-infused behavioral parent training
S0251	C0240	Mindfulness Therapy	mindfulness-infused behavioral parent training
S0251	C0241	Mindfulness Therapy	mindfulness-infused behavioral parent training
S0253	C0243	Behavior Modification Therapy (BMT)	contingency management strategy
S0253	C0244	Behavior Modification Therapy (BMT)	contingency management strategy
S0253	C0245	Behavior Modification Therapy (BMT)	contingency management strategy
S0255	C0246	Behavior Modification Therapy (BMT)	Response Cost-Based Treatment Package
S0255	C0247	Behavior Modification Therapy (BMT)	Response Cost-Based Treatment Package
S0255	C0248	Behavior Modification Therapy (BMT)	Response Cost-Based Treatment Package
S0255	C0249	Behavior Modification Therapy (BMT)	Response Cost-Based Treatment Package
S0258	C0250	Cognitive-Behavioral Therapy (CBT)	Functional Analytic Psychotherapy (FAP)
S0258	C0251	Cognitive-Behavioral Therapy (CBT)	Functional Analytic Psychotherapy (FAP)
S0258	C0252	Cognitive-Behavioral Therapy (CBT)	Functional Analytic Psychotherapy (FAP)
S0258	C0253	Cognitive-Behavioral Therapy (CBT)	Functional Analytic Psychotherapy (FAP)
S0262	C0255	Behavior Modification Therapy (BMT) + Social Skills Training (SST)	Check-in, Check-out (CICO) + Social Skills
S0262	C0256	Behavior Modification Therapy (BMT) + Social Skills Training (SST)	Check-in, Check-out (CICO) + Social Skills
S0262	C0257	Behavior Modification Therapy (BMT) + Social Skills Training (SST)	Check-in, Check-out (CICO) + Social Skills
S0262	C0258	Behavior Modification Therapy (BMT) + Social Skills Training (SST)	Check-in, Check-out (CICO) + Social Skills
S0262	C0259	Behavior Modification Therapy (BMT) + Social Skills Training (SST)	Check-in, Check-out (CICO) + Social Skills
S0267	C0263	Self-Monitoring (SM)	Video Self-Modeling (VSM)
S0267	C0264	Self-Monitoring (SM)	Video Self-Modeling (VSM)
S0267	C0265	Self-Monitoring (SM)	Video Self-Modeling (VSM)
S0282	C0266	Parent Behavior Training	Theraplay
S0282	C0267	Parent Behavior Training	Theraplay
S0282	C0268	Parent Behavior Training	Theraplay

S0282	C0269	Parent Behavior Training	Theraplay
S0293	C0270	Mindfulness Therapy	Meditation on the Soles of the Feet (SoF)
S0293	C0271	Mindfulness Therapy	Meditation on the Soles of the Feet (SoF)
S0294	C0273	Mindfulness Therapy	Meditation on the Soles of the Feet (SoF)
S0294	C0274	Mindfulness Therapy	Meditation on the Soles of the Feet (SoF)
S0294	C0275	Mindfulness Therapy	Meditation on the Soles of the Feet (SoF)
S0296	C0276	Mindfulness Therapy	Surfind the Urge
S0296	C0277	Mindfulness Therapy	Surfind the Urge
S0296	C0278	Mindfulness Therapy	Surfind the Urge
S0300	C0279	Cognitive-Behavioral Therapy (CBT)	ZIPPER strategy
S0300	C0280	Cognitive-Behavioral Therapy (CBT)	ZIPPER strategy
S0300	C0281	Cognitive-Behavioral Therapy (CBT)	ZIPPER strategy
S0304	C0282	Behavior Modification Therapy (BMT)	First Step to Sucess
S0304	C0283	Behavior Modification Therapy (BMT)	First Step to Sucess
S0304	C0284	Behavior Modification Therapy (BMT)	First Step to Sucess
S0304	C0285	Behavior Modification Therapy (BMT)	First Step to Sucess
S0313	C0286	Behavior Modification Therapy (BMT)	Adlerian Play Therapy
S0313	C0287	Behavior Modification Therapy (BMT)	Adlerian Play Therapy
S0313	C0288	Behavior Modification Therapy (BMT)	Adlerian Play Therapy
S0313	C0289	Behavior Modification Therapy (BMT)	Adlerian Play Therapy
S0319	C352	Cognitive-Behavioral Therapy (CBT)	Cognitive Behavior Modification Treatment Package (Verbal Self-Instruction; Self Monitoring)
S0329	C0291	Behavior Modification Therapy (BMT)	Color Wheel System
S0329	C0292	Behavior Modification Therapy (BMT)	Color Wheel System
S0329	C0293	Behavior Modification Therapy (BMT)	Color Wheel System
S0329	C0294	Behavior Modification Therapy (BMT)	Color Wheel System
S0329	C0295	Behavior Modification Therapy (BMT)	Color Wheel System
S0329	C0296	Behavior Modification Therapy (BMT)	Color Wheel System
S0329	C0297	Behavior Modification Therapy (BMT)	Color Wheel System
S0329	C0298	Behavior Modification Therapy (BMT)	Color Wheel System
S0329	C0299	Behavior Modification Therapy (BMT)	Color Wheel System
S0331	C0300	Social Skills Training (SST)	In Vivo Social Skills Training (SST)

S0336	C0301	Social Skills Training (SST)	Functional Communication Training (FCT)
S0336	C0302	Social Skills Training (SST)	Functional Communication Training (FCT)
S0336	C0303	Social Skills Training (SST)	Functional Communication Training (FCT)
S0336	C0304	Social Skills Training (SST)	Functional Communication Training (FCT)
S0336	C0305	Social Skills Training (SST)	Functional Communication Training (FCT)
S0336	C0306	Social Skills Training (SST)	Functional Communication Training (FCT)
S0341	C0307	Behavior Modification Therapy (BMT)	Daily Report Card (DRC)
S0342	C0308	Behavior Modification Therapy (BMT)	behavior modification interventions
S0342	C0309	Behavior Modification Therapy (BMT)	behavior modification interventions
S0345	C0310	Behavior Modification Therapy (BMT)	Contingency Contracting
S0345	C0311	Behavior Modification Therapy (BMT)	Contingency Contracting
S0345	C0312	Behavior Modification Therapy (BMT)	Contingency Contracting
S0345	C0313	Behavior Modification Therapy (BMT)	Contingency Contracting
S0345	C0314	Behavior Modification Therapy (BMT)	Contingency Contracting
S0345	C0315	Behavior Modification Therapy (BMT)	Contingency Contracting
S0358	C0316	Behavior Modification Therapy (BMT)	behavior modification interventions
S0359	C0317	Behavior Modification Therapy (BMT)	School-Home Note (SHN)
S0359	C0318	Behavior Modification Therapy (BMT)	School-Home Note (SHN)
S0359	C0319	Behavior Modification Therapy (BMT)	Behavioral Contract (BC)
S0359	C0320	Behavior Modification Therapy (BMT)	Behavioral Contract (BC)
S0359	C0321	Behavior Modification Therapy (BMT)	Behavioral Contract (BC)
S0359	C0322	Behavior Modification Therapy (BMT)	Behavioral Contract (BC)
S0362	C0341	Self-Monitoring (SM)	I-Connect
S0362	C0342	Self-Monitoring (SM)	I-Connect
S0362	C0343	Self-Monitoring (SM)	I-Connect
S0363	C0344	relationship-based intervention	Banking Time
S0363	C0345	relationship-based intervention	Banking Time
S0363	C0346	relationship-based intervention	Banking Time
S0363	C0347	relationship-based intervention	Banking Time
S0363	C0348	relationship-based intervention	Banking Time

S0363	C0349	relationship-based intervention	Banking Time

Supplement #3I – Child Characteristics Part 7

study_id	child_id	treatment_provider	treatment_length	num_sessions_intervention	treatment_target
S0005	C0001	clinician	unclear	8	child
S0007	C0002	researcher	3 weeks	Unclear	child
S0007	C0003	researcher	3 weeks	Unclear	child
S0007	C0004	researcher	3 weeks	Unclear	child
S0007	C0005	researcher	3 weeks	Unclear	child
S0013	C0006	clinician	5 months	11	parents and child
S0016	C0013	clinician	3 weeks	12	child
S0016	C0014	clinician	3 weeks	12	child
S0016	C0015	clinician	3 weeks	12	child
S0017	C0016	teacher	unclear	Unclear	child
S0017	C0017	teacher	unclear	Unclear	child
S0017	C0018	teacher	unclear	Unclear	child
S0017	C0019	teacher	unclear	Unclear	child
S0017	C0020	teacher	unclear	Unclear	child
S0017	C0021	teacher	unclear	Unclear	child
S0024	C0025	clinician	10 weeks	Unclear	child
S0024	C0026	clinician	11 weeks	Unclear	child
S0024	C0027	clinician	10 weeks	Unclear	child
S0026	C0028	teacher	2 weeks	Unclear	child
S0026	C0029	teacher	2 weeks	Unclear	child
S0026	C0030	teacher	2 weeks	Unclear	child
S0028	C0031	teacher	6 weeks	5	teacher
S0028	C0032	teacher	6 weeks	5	teacher
S0028	C0033	teacher	6 weeks	5	teacher
S0028	C0034	teacher	6 weeks	5	teacher
S0028	C0035	teacher	6 weeks	5	teacher
S0028	C0036	teacher	6 weeks	5	teacher
S0037	C0039	clinician	unclear	Unclear	child

S0037	C0041	clinician	unclear	Unclear	child
S0037	C0042	clinician	unclear	Unclear	child
S0040	C0044	social worker	12 weeks	Unclear	parents and child
S0047	C0045	teacher	unclear	Unclear	child
S0047	C0046	teacher	unclear	Unclear	child
S0047	C0047	teacher	unclear	Unclear	child
S0048	C0048	teacher	6 weeks	Unclear	child
S0048	C0049	teacher	6 weeks	Unclear	child
S0048	C0050	teacher	6 weeks	Unclear	child
S0048	C0051	teacher	6 weeks	Unclear	child
S0048	C0052	teacher	6 weeks	Unclear	child
S0048	C0053	teacher	6 weeks	Unclear	child
S0048	C0054	teacher	6 weeks	Unclear	child
S0048	C0055	teacher	6 weeks	Unclear	child
S0048	C0056	teacher	6 weeks	Unclear	child
S0048	C0057	teacher	6 weeks	Unclear	child
S0048	C0058	teacher	6 weeks	Unclear	child
S0048	C0059	teacher	6 weeks	Unclear	child
S0050	C0060	teacher	unclear	Unclear	child
S0050	C0061	teacher	unclear	Unclear	child
S0050	C0062	teacher	unclear	Unclear	child
S0051	C0063	intervention coach & teacher	unclear	Unclear	child
S0062	C0064	researcher	7 weeks	5	parents and child
S0062	C0065	researcher	7 weeks	5	parents and child
S0062	C0066	researcher	7 weeks	4	parents and child
S0070	C0068	researcher	10 weeks	Unclear	parents and child
S0070	C0069	researcher	10 weeks	Unclear	parents and child
S0070	C0070	researcher	10 weeks	Unclear	parents and child
S0071	C0071	researcher	unclear	8	parents and child
S0071	C0072	researcher	unclear	8	parents and child
S0071	C0073	researcher	unclear	8	parents and child

S0071	C0074	researcher	unclear	8	parents and child
S0071	C0075	researcher	unclear	8	parents and child
S0071	C0076	researcher	unclear	8	parents and child
S0071	C0077	researcher	unclear	8	parents and child
S0071	C0078	researcher	unclear	8	parents and child
S0075	C0091	school psychologist	4 weeks	20 sessions	child
S0084	C0092	doctoral student	unclear - 7 weeks	35	child
S0084	C0093	doctoral student	unclear - 7 weeks	35	child
S0096	C0100	teacher	unclear	Unclear	child
S0096	C0101	teacher	unclear	Unclear	child
S0096	C0102	teacher	unclear	Unclear	child
S0096	C0103	teacher	unclear	Unclear	child
S0096	C0104	teacher	unclear	Unclear	child
S0096	C0105	teacher	unclear	Unclear	child
S0101	C0106	researcher	3 month	11	parents and child
S0108	C0107	clinician	29 weeks	29	parents and child
S0113	C0109	teacher	unclear	Unclear	child
S0113	C0110	teacher	unclear	Unclear	child
S0113	C0111	teacher	unclear	Unclear	child
S0113	C0112	teacher	unclear	Unclear	child
S0113	C0113	teacher	unclear	Unclear	child
S0113	C0114	teacher	unclear	Unclear	child
S0116	C0115	clinician	10 sessions	10	both (parents and child)
S0120	C0116	parent	13 weeks	18	parents and child
S0120	C0117	parent	13 weeks	17	parents and child
S0120	C0118	parent	13 weeks	22	parents and child
S0120	C0119	parent	8 weeks	11	parents and child
S0122	C0120	parent	6 weeks	7	parents and teacher
S0122	C0121	parent	6 weeks	7	parents and teacher
S0122	C0122	parent	6 weeks	7	parents and teacher
S0123	C0126	parent	8 weeks	Unclear	parents and teacher

S0123	C0128	parent	8 weeks	Unclear	parents and child
S0123	C0131	parent	8 weeks	Unclear	parents and child
S0123	C0132	parent	8 weeks	Unclear	parents and child
S0124	C0142	researcher	unclear	Unclear	child
S0124	C0143	researcher	unclear	Unclear	child
S0125	C0145	teacher	unclear	Unclear	child
S0129	C0146	teacher	15 weeks	29	child
S0129	C0147	teacher	15 weeks	29	child
S0129	C0148	teacher	15 weeks	29	child
S0129	C0149	teacher	15 weeks	29	child
S0129	C0150	teacher	15 weeks	29	child
S0129	C0151	teacher	15 weeks	29	child
S0129	C0152	teacher	15 weeks	29	child
S0129	C0153	teacher	15 weeks	29	child
S0129	C0154	teacher	15 weeks	29	child
S0129	C0155	teacher	15 weeks	29	child
S0129	C0156	teacher	15 weeks	29	child
S0134	C0157	parent	3 weeks	Unclear	child
S0134	C0158	parent	8 weeks	Unclear	child
S0136	C0160	parent	2 month	Unclear	parents and child
S0136	C0161	parent	2 month	Unclear	parents and child
S0136	C0162	parent	2 month	Unclear	parents and child
S0138	C0163	parent	13 weeks	13	parents and child
S0138	C0164	parent	13 weeks	13	parents and child
S0138	C0165	parent	13 weeks	13	parents and child
S0147	C0323	parent	17 weeks	17	parents and child
S0147	C0324	parent	19 weeks	19	parents and child
S0153	C0166	teacher	12 weeks	Unclear	child
S0153	C0167	teacher	12 weeks	Unclear	child
S0153	C0168	teacher	12 weeks	Unclear	child
S0154	C0169	teacher	9 weeks	13	child

S0154	C0170	teacher	7 weeks	16	child
S0154	C0171	teacher	9 weeks	16	child
S0154	C0172	teacher	8 weeks	18	child
S0155	C0173	researcher	unclear	7	child
S0155	C0174	researcher	unclear	7	child
S0155	C0175	researcher	unclear	7	child
S0156	C0176	researcher	5 days	5	child
S0156	C0177	researcher	5 days	5	child
S0156	C0178	researcher	5 days	5	child
S0159	C0180	researcher	40 days	40	child
S0159	C0181	researcher	35 days	35	child
S0159	C0182	researcher	30 days	30	child
S0159	C0183	researcher	26 days	26	child
S0159	C0184	researcher	16 days	16	child
S0159	C0185	researcher	14 day	14	child
S0159	C0186	researcher	9 days	9	child
S0159	C0187	researcher	4 day	4	child
S0159	C0188	researcher	8 day	8	child
S0159	C0189	researcher	7 days	7	child
S0159	C0190	researcher	4 days	4	child
S0165	C0191	clinician	unclear	18	both (parents and child)
S0167	C0325	parent	unclear	Unclear	parents and child
S0170	C0192	teacher	unclear	Unclear	child
S0170	C0193	teacher	unclear	Unclear	child
S0170	C0194	teacher	unclear	Unclear	child
S0170	C0195	teacher	unclear	Unclear	child
S0173	C0196	teacher	unclear	Unclear	child
S0173	C0197	teacher	unclear	Unclear	child
S0173	C0198	teacher	unclear	Unclear	child
S0173	C0199	teacher	unclear	Unclear	child
S0180	C0200	researcher	unclear	Unclear	child

S0180	C0201	researcher	unclear	Unclear	child
S0192	C0205	researcher	6 weeks	12	child
S0192	C0206	researcher	6 weeks	12	child
S0192	C0207	researcher	6 weeks	12	child
S0192	C0208	researcher	6 weeks	12	child
S0197	C0211	teacher	unclear	Unclear	child
S0197	C0212	teacher	unclear	Unclear	child
S0197	C0213	teacher	unclear	Unclear	child
S0198	C0214	parent	unclear	16	parents and child
S0198	C0215	parent	unclear	21	parents and child
S0198	C0216	parent	unclear	18	parents and child
S0202	C0217	researcher + teacher	unclear	Unclear	child
S0202	C0218	researcher + teacher	unclear	Unclear	child
S0202	C0219	researcher + teacher	unclear	Unclear	child
S0207	C0220	researcher	4 weeks	8	child
S0207	C0221	researcher	4 weeks	8	child
S0207	C0222	researcher	4 weeks	8	child
S0207	C0224	researcher	4 weeks	8	child
S0214	C0225	teacher	7 weeks	Unclear	child
S0214	C0226	teacher	7 weeks	Unclear	child
S0214	C0227	teacher	7 weeks	Unclear	child
S0233	C0228	researcher	unclear	Unclear	child
S0233	C0229	researcher	unclear	Unclear	child
S0234	C0230	teacher and caregiver	6 weeks	6	child
S0234	C0231	teacher and caregiver	5 weeks	5	child
S0234	C0232	teacher and caregiver	4 weeks	4	child
S0246	C0234	clinician	7 months	24	parents and child
S0248	C0235	clinician	unclear	Unclear	child
S0248	C0236	clinician	unclear	Unclear	child
S0248	C0237	clinician	unclear	Unclear	child
S0248	C0238	clinician	unclear	Unclear	child

S0251	C0239	parent	unclear	Unclear	parents and child
S0251	C0240	parent	unclear	Unclear	parents and child
S0251	C0241	parent	unclear	Unclear	parents and child
S0253	C0243	teacher	35 days	Unclear	child
S0253	C0244	teacher	35 days	Unclear	child
S0253	C0245	teacher	35 days	Unclear	child
S0255	C0246	teacher	unclear	Unclear	child
S0255	C0247	teacher	unclear	Unclear	child
S0255	C0248	teacher	unclear	Unclear	child
S0255	C0249	teacher	unclear	Unclear	child
S0258	C0250	parent	unclear	12	parents and child
S0258	C0251	parent	unclear	12	parents and child
S0258	C0252	parent	unclear	12	parents and child
S0258	C0253	parent	unclear	12	parents and child
S0262	C0255	teacher	7 weeks	Unclear	child
S0262	C0256	teacher	5 weeks	Unclear	child
S0262	C0257	teacher	6 weeks	Unclear	child
S0262	C0258	teacher	6 weeks	Unclear	child
S0262	C0259	teacher	5 weeks	Unclear	child
S0267	C0263	researcher	unclear	Unclear	child
S0267	C0264	researcher	unclear	Unclear	child
S0267	C0265	researcher	unclear	Unclear	child
S0282	C0266	clinician	15 weeks	15	parents and child
S0282	C0267	clinician	15 weeks	15	parents and child
S0282	C0268	clinician	15 weeks	15	parents and child
S0282	C0269	clinician	15 weeks	15	parents and child
S0293	C0270	parent	17 weeks	119	child
S0293	C0271	parent	22 weeks	154	child
S0294	C0273	clinician	unclear	Unclear	child
S0294	C0274	clinician	unclear	Unclear	child
S0294	C0275	clinician	unclear	Unclear	child

S0296	C0276	clinician	unclear	Unclear	child
S0296	C0277	clinician	unclear	Unclear	child
S0296	C0278	clinician	unclear	Unclear	child
S0300	C0279	clinician	6 days	Unclear	child
S0300	C0280	clinician	6 days	Unclear	child
S0300	C0281	clinician	6 days	Unclear	child
S0304	C0282	teacher	unclear	Unclear	child
S0304	C0283	teacher	unclear	Unclear	child
S0304	C0284	teacher	unclear	Unclear	child
S0304	C0285	teacher	unclear	Unclear	child
S0313	C0286	clinician	14 weeks	28	child
S0313	C0287	clinician	14 weeks	28	child
S0313	C0288	clinician	14 weeks	28	child
S0313	C0289	clinician	14 weeks	28	child
S0319	C352	clinician	4 weeks	16	child
S0329	C0291	teacher	unclear	Unclear	child
S0329	C0292	teacher	unclear	Unclear	child
S0329	C0293	teacher	unclear	Unclear	child
S0329	C0294	teacher	unclear	Unclear	child
S0329	C0295	teacher	unclear	Unclear	child
S0329	C0296	teacher	unclear	Unclear	child
S0329	C0297	teacher	unclear	Unclear	child
S0329	C0298	teacher	unclear	Unclear	child
S0329	C0299	teacher	unclear	Unclear	child
S0331	C0300	clinician	3 weeks	Unclear	child
S0336	C0301	parent	unclear	Unclear	child
S0336	C0302	parent	unclear	Unclear	child
S0336	C0303	parent	unclear	Unclear	child
S0336	C0304	parent	unclear	Unclear	child
S0336	C0305	parent	unclear	Unclear	child
S0336	C0306	parent	unclear	Unclear	child

S0341	C0307	clinician	8 weeks	39	child
S0342	C0308	teacher	unclear	40	child
S0342	C0309	teacher	unclear	40	child
S0345	C0310	clinician	unclear	3	child
S0345	C0311	clinician	unclear	4	child
S0345	C0312	clinician	unclear	5	child
S0345	C0313	clinician	unclear	6	child
S0345	C0314	clinician	unclear	7	child
S0345	C0315	clinician	unclear	8	child
S0358	C0316	mother	unclear	12	child
S0359	C0317	teacher	unclear	Unclear	child
S0359	C0318	teacher	unclear	Unclear	child
S0359	C0319	teacher	unclear	Unclear	child
S0359	C0320	teacher	unclear	Unclear	child
S0359	C0321	teacher	unclear	Unclear	child
S0359	C0322	teacher	unclear	Unclear	child
S0362	C0341	teacher	unclear	13	child
S0362	C0342	teacher	unclear	12	child
S0362	C0343	teacher	unclear	14	child
S0363	C0344	teacher	3-8 weeks	9	child
S0363	C0345	teacher	3-8 weeks	9	child
S0363	C0346	teacher	3-8 weeks	9	child
S0363	C0347	teacher	3-8 weeks	8	child
S0363	C0348	teacher	3-8 weeks	8	child
S0363	C0349	teacher	3-8 weeks	7	child

Supplement #3J – Child Characteristics Part 8

study _id	child_id	provider_training
S0005	C0001	NR
S0007	C0002	NR
S0007	C0003	NR
S0007	C0004	NR
S0007	C0005	NR
S0013	C0006	NR
S0016	C0013	NR
S0016	C0014	NR
S0016	C0015	NR
S0017	C0016	Teachers and teachers aides received training in behavior management strategies
S0017	C0017	Teachers and teachers aides received training in behavior management strategies
S0017	C0018	Teachers and teachers aides received training in behavior management strategies
S0017	C0019	Teachers and teachers aides received training in behavior management strategies
S0017	C0020	Teachers and teachers aides received training in behavior management strategies
S0017	C0021	Teachers and teachers aides received training in behavior management strategies
S0024	C0025	NR
S0024	C0026	NR
S0024	C0027	NR
S0026	C0028	NR
S0026	C0029	NR
S0026	C0030	NR
S0028	C0031	NR
S0028	C0032	NR
S0028	C0033	NR
S0028	C0034	NR
S0028	C0035	NR

S0028	C0036	NR
S0037	C0039	NR
S0037	C0041	NR
S0037	C0042	NR
S0040	C0044	trained in behavioral assessment and intervention strategies
S0047	C0045	Teachers were instructed to give one warning in the form of the following statement, 'Do (this) or you owe me a TO,' with the specific instruction inserted in the statement regarding rule violation (e.g., 'Sit in your chair or you owe me a TO,'). They were further trained to notify other adults in the room that the target student owed a TO and that all interaction with the student should be limited. Teachers were instructed to ignore disruptive behavior commonly following refused requests. TO began when the student went to the designated TO spot in the classroom with release contingent on appropriate behavior and was not to exceed three minutes. Following TO, the teacher was instructed to reinforce the next occurrence of appropriate behavior so that the student experienced a difference between time-in and TO. Understanding of intervention was determined by the teacher’s ability to explain DTO procedures and model the intervention accurately during training sessions with the researcher
S0047	C0046	Teachers were instructed to give one warning in the form of the following statement, 'Do (this) or you owe me a TO,' with the specific instruction inserted in the statement regarding rule violation (e.g., 'Sit in your chair or you owe me a TO,'). They were further trained to notify other adults in the room that the target student owed a TO and that all interaction with the student should be limited. Teachers were instructed to ignore disruptive behavior commonly following refused requests. TO began when the student went to the designated TO spot in the classroom with release contingent on appropriate behavior and was not to exceed three minutes. Following TO, the teacher was instructed to reinforce the next occurrence of appropriate behavior so that the student experienced a difference between time-in and TO. Understanding of intervention was determined by the teacher’s ability to explain DTO procedures and model the intervention accurately during training sessions with the researcher
S0047	C0047	Teachers were instructed to give one warning in the form of the following statement, 'Do (this) or you owe me a TO,' with the specific instruction inserted in the statement regarding rule violation (e.g., 'Sit in your chair or you owe me a TO,'). They were further trained to notify other adults in the room that the target student owed a TO and that all interaction with the student should be limited. Teachers were instructed to ignore disruptive behavior commonly following refused requests. TO began when the student went to the designated TO spot in the classroom with release contingent on appropriate behavior and was not to exceed three minutes. Following TO, the teacher was instructed to reinforce the next occurrence of appropriate behavior so that the student experienced a difference between time-in and TO. Understanding of intervention was determined by the teacher’s ability to explain DTO procedures and model the intervention accurately during training sessions with the researcher
S0048	C0048	Material Provided; Training Sessions; CICO training
S0048	C0049	Material Provided; Training Sessions; CICO training
S0048	C0050	Material Provided; Training Sessions; CICO training
S0048	C0051	Material Provided; Training Sessions; CICO training
S0048	C0052	Material Provided; Training Sessions; CICO training
S0048	C0053	Material Provided; Training Sessions; CICO training
S0048	C0054	Material Provided; Training Sessions; CICO training
S0048	C0055	Material Provided; Training Sessions; CICO training
S0048	C0056	Material Provided; Training Sessions; CICO training
S0048	C0057	Material Provided; Training Sessions; CICO training
S0048	C0058	Material Provided; Training Sessions; CICO training

S0048	C0059	Material Provided; Training Sessions; CICO training
S0050	C0060	NR
S0050	C0061	NR
S0050	C0062	NR
S0051	C0063	coach coordinated the implementation process and ran the program in the classroom; demonstrating the program and training the teacher in how to apply it; providing the teacher with initial supervision as she began to take over program implementation
S0062	C0064	six months of classes that focused on Solution-Focused Family Therapy
S0062	C0065	six months of classes that focused on Solution-Focused Family Therapy
S0062	C0066	six months of classes that focused on Solution-Focused Family Therapy
S0070	C0068	NR
S0070	C0069	NR
S0070	C0070	NR
S0071	C0071	NR
S0071	C0072	NR
S0071	C0073	NR
S0071	C0074	NR
S0071	C0075	NR
S0071	C0076	NR
S0071	C0077	NR
S0071	C0078	NR
S0075	C0091	NR
S0084	C0092	NR
S0084	C0093	NR
S0096	C0100	providers were trained by research staff through detailed instruction, modeling, and the use of checklists, with fidelity monitored through intermittent observations
S0096	C0101	providers were trained by research staff through detailed instruction, modeling, and the use of checklists, with fidelity monitored through intermittent observations
S0096	C0102	providers were trained by research staff through detailed instruction, modeling, and the use of checklists, with fidelity monitored through intermittent observations
S0096	C0103	providers were trained by research staff through detailed instruction, modeling, and the use of checklists, with fidelity monitored through intermittent observations
S0096	C0104	providers were trained by research staff through detailed instruction, modeling, and the use of checklists, with fidelity monitored through intermittent observations

S0096	C0105	providers were trained by research staff through detailed instruction, modeling, and the use of checklists, with fidelity monitored through intermittent observations
S0101	C0106	NR
S0108	C0107	40 hours of a PCIT-Japan initial workshop
S0113	C0109	Two on-site training sessions, each lasting about 60 minutes
S0113	C0110	Two on-site training sessions, each lasting about 60 minutes
S0113	C0111	Two on-site training sessions, each lasting about 60 minutes
S0113	C0112	Two on-site training sessions, each lasting about 60 minutes
S0113	C0113	Two on-site training sessions, each lasting about 60 minutes
S0113	C0114	Two on-site training sessions, each lasting about 60 minutes
S0116	C0115	NR
S0120	C0116	NR
S0120	C0117	NR
S0120	C0118	NR
S0120	C0119	NR
S0122	C0120	NR
S0122	C0121	NR
S0122	C0122	NR
S0123	C0126	5h training
S0123	C0128	5h training
S0123	C0131	5h training
S0123	C0132	5h training
S0124	C0142	NR
S0124	C0143	NR
S0125	C0145	NR
S0129	C0146	4h intensive training
S0129	C0147	4h intensive training
S0129	C0148	4h intensive training
S0129	C0149	4h intensive training

S0129	C0150	4h intensive training
S0129	C0151	4h intensive training
S0129	C0152	4h intensive training
S0129	C0153	4h intensive training
S0129	C0154	4h intensive training
S0129	C0155	4h intensive training
S0129	C0156	4h intensive training
S0134	C0157	written instruction; procedural demonstrations; prescriptive feedback; coaching sessions
S0134	C0158	written instruction; procedural demonstrations; prescriptive feedback; coaching sessions
S0136	C0160	written and verbal instructions; modeling, role-playing; in-the-moment feedback using bug-in-ear technology; and follow-up emails
S0136	C0161	written and verbal instructions; modeling, role-playing; in-the-moment feedback using bug-in-ear technology; and follow-up emails
S0136	C0162	written and verbal instructions; modeling, role-playing; in-the-moment feedback using bug-in-ear technology; and follow-up emails
S0138	C0163	Modeling; Live Coaching; Immediate Feedback
S0138	C0164	Modeling; Live Coaching; Immediate Feedback
S0138	C0165	Modeling; Live Coaching; Immediate Feedback
S0147	C0323	trained through live coaching sessions with a trained therapist
S0147	C0324	trained through live coaching sessions with a trained therapist
S0153	C0166	6h of CICO training
S0153	C0167	6h of CICO training
S0153	C0168	6h of CICO training
S0154	C0169	12 h training
S0154	C0170	12 h training
S0154	C0171	12 h training
S0154	C0172	12 h training
S0155	C0173	completed an online instructor training for the COPE program
S0155	C0174	completed an online instructor training for the COPE program
S0155	C0175	completed an online instructor training for the COPE program
S0156	C0176	NR
S0156	C0177	NR

S0156	C0178	NR
S0159	C0180	NR
S0159	C0181	NR
S0159	C0182	NR
S0159	C0183	NR
S0159	C0184	NR
S0159	C0185	NR
S0159	C0186	NR
S0159	C0187	NR
S0159	C0188	NR
S0159	C0189	NR
S0159	C0190	NR
S0165	C0191	NR
S0167	C0325	didactic teaching and live coaching
S0170	C0192	training on Social Stories
S0170	C0193	training on Social Stories
S0170	C0194	training on Social Stories
S0170	C0195	training on Social Stories
S0173	C0196	training on App (App = MoBeGo)
S0173	C0197	training on App (App = MoBeGo)
S0173	C0198	training on App (App = MoBeGo)
S0173	C0199	training on App (App = MoBeGo)
S0180	C0200	NR
S0180	C0201	NR
S0192	C0205	NR
S0192	C0206	NR
S0192	C0207	NR
S0192	C0208	NR
S0197	C0211	individual sessions on intervention

S0197	C0212	individual sessions on intervention
S0197	C0213	individual sessions on intervention
S0198	C0214	didactic instructions; real-time coaching; homework assignments; progress monitoring; graduation criteria
S0198	C0215	didactic instructions; real-time coaching; homework assignments; progress monitoring; graduation criteria
S0198	C0216	didactic instructions; real-time coaching; homework assignments; progress monitoring; graduation criteria
S0202	C0217	NR
S0202	C0218	NR
S0202	C0219	NR
S0207	C0220	NR
S0207	C0221	NR
S0207	C0222	NR
S0207	C0224	NR
S0214	C0225	NR
S0214	C0226	NR
S0214	C0227	NR
S0233	C0228	NR
S0233	C0229	NR
S0234	C0230	NR
S0234	C0231	NR
S0234	C0232	NR
S0246	C0234	NR
S0248	C0235	NR
S0248	C0236	NR
S0248	C0237	NR
S0248	C0238	NR
S0251	C0239	researcher provided training; 3 weekly sessions
S0251	C0240	researcher provided training; 3 weekly sessions
S0251	C0241	researcher provided training; 3 weekly sessions
S0253	C0243	NR

S0253	C0244	NR
S0253	C0245	NR
S0255	C0246	teachers were given a handout explaining the intervention and were instructed on how to use it
S0255	C0247	teachers were given a handout explaining the intervention and were instructed on how to use it
S0255	C0248	teachers were given a handout explaining the intervention and were instructed on how to use it
S0255	C0249	teachers were given a handout explaining the intervention and were instructed on how to use it
S0258	C0250	mothers attended therapy with clinician and then implemented intervention with children
S0258	C0251	mothers attended therapy with clinician and then implemented intervention with children
S0258	C0252	mothers attended therapy with clinician and then implemented intervention with children
S0258	C0253	mothers attended therapy with clinician and then implemented intervention with children
S0262	C0255	teachers received a training session
S0262	C0256	teachers received a training session
S0262	C0257	teachers received a training session
S0262	C0258	teachers received a training session
S0262	C0259	teachers received a training session
S0267	C0263	NR
S0267	C0264	NR
S0267	C0265	NR
S0282	C0266	NR
S0282	C0267	NR
S0282	C0268	NR
S0282	C0269	NR
S0293	C0270	mothers were trainined by experienced SoF trainer
S0293	C0271	mothers were trainined by experienced SoF trainer
S0294	C0273	NR
S0294	C0274	NR
S0294	C0275	NR
S0296	C0276	NR
S0296	C0277	NR

S0296	C0278	NR
S0300	C0279	NR
S0300	C0280	NR
S0300	C0281	NR
S0304	C0282	NR
S0304	C0283	NR
S0304	C0284	NR
S0304	C0285	NR
S0313	C0286	NR
S0313	C0287	NR
S0313	C0288	NR
S0313	C0289	NR
S0319	C352	NR
S0329	C0291	researcher provided an overview of the color wheel system
S0329	C0292	researcher provided an overview of the color wheel system
S0329	C0293	researcher provided an overview of the color wheel system
S0329	C0294	researcher provided an overview of the color wheel system
S0329	C0295	researcher provided an overview of the color wheel system
S0329	C0296	researcher provided an overview of the color wheel system
S0329	C0297	researcher provided an overview of the color wheel system
S0329	C0298	researcher provided an overview of the color wheel system
S0329	C0299	researcher provided an overview of the color wheel system
S0331	C0300	NR
S0336	C0301	parents were coached by clinician
S0336	C0302	parents were coached by clinician
S0336	C0303	parents were coached by clinician
S0336	C0304	parents were coached by clinician
S0336	C0305	parents were coached by clinician
S0336	C0306	parents were coached by clinician

S0341	C0307	NR
S0342	C0308	training in behavior modification principles
S0342	C0309	training in behavior modification principles
S0345	C0310	masters degree
S0345	C0311	masters degree
S0345	C0312	masters degree
S0345	C0313	masters degree
S0345	C0314	masters degree
S0345	C0315	masters degree
S0358	C0316	received instructions from the experimenters; included immediate response to aggressive or disobedient behavior with time-out; specific verbal cues; and minimal explanations; The mother was also cued by the experimenter when necessary and received social approval or corrections based on her adherence to the instructions
S0359	C0317	trained in precision-based approach (PBA)
S0359	C0318	trained in precision-based approach (PBA)
S0359	C0319	trained in precision-based approach (PBA)
S0359	C0320	trained in precision-based approach (PBA)
S0359	C0321	trained in precision-based approach (PBA)
S0359	C0322	trained in precision-based approach (PBA)
S0362	C0341	30-min face-to-face training in group setting; individual coaching; performance feedback
S0362	C0342	30-min face-to-face training in group setting; individual coaching; performance feedback
S0362	C0343	30-min face-to-face training in group setting; individual coaching; performance feedback
S0363	C0344	initial workshop; modeling and demonstration of techniques; practice and role-playing exercises; and ongoing coaching and feedback to ensure fidelity to the intervention.
S0363	C0345	initial workshop; modeling and demonstration of techniques; practice and role-playing exercises; and ongoing coaching and feedback to ensure fidelity to the intervention.
S0363	C0346	initial workshop; modeling and demonstration of techniques; practice and role-playing exercises; and ongoing coaching and feedback to ensure fidelity to the intervention.
S0363	C0347	initial workshop; modeling and demonstration of techniques; practice and role-playing exercises; and ongoing coaching and feedback to ensure fidelity to the intervention.
S0363	C0348	initial workshop; modeling and demonstration of techniques; practice and role-playing exercises; and ongoing coaching and feedback to ensure fidelity to the intervention.
S0363	C0349	initial workshop; modeling and demonstration of techniques; practice and role-playing exercises; and ongoing coaching and feedback to ensure fidelity to the intervention.

Supplement #3K – Child Characteristics Part 9

study_id	child_id	freq_assessment_cat	behavior_type	freq_assessment_symptom
S0005	C0001	disruptive behavior; Rumination	Irretable and angry behavior	frequency and intensity of anger episodes; intensitiy of complaints
S0007	C0002	disruptive behavior	Aggressive behavior	average externalizing behavior scale response (EBS)
S0007	C0003	disruptive behavior	Antisocial behavior	average externalizing behavior scale response (EBS)
S0007	C0004	disruptive behavior	Antisocial behavior	average externalizing behavior scale response (EBS)
S0007	C0005	disruptive behavior	Antisocial behavior	average externalizing behavior scale response (EBS)
S0013	C0006	disruptive behavior	Other	disruptive behavior; lying and stealing; aggression
S0016	C0013	compliance	Defiant and oppositional behavior	percentage of compliance (in hospital unit and in classroom)
S0016	C0014	compliance	Defiant and oppositional behavior	percentage of compliance (in hospital unit and in classroom)
S0016	C0015	compliance	Defiant and oppositional behavior	percentage of compliance (in hospital unit and in classroom)
S0017	C0016	externalizing problems	Defiant and oppositional behavior	observation of externalizing problems
S0017	C0017	externalizing problems	Defiant and oppositional behavior	observation of externalizing problems
S0017	C0018	externalizing problems	Defiant and oppositional behavior	observation of externalizing problems
S0017	C0019	externalizing problems	Defiant and oppositional behavior	observation of externalizing problems
S0017	C0020	externalizing problems	Defiant and oppositional behavior	observation of externalizing problems
S0017	C0021	externalizing problems	Defiant and oppositional behavior	observation of externalizing problems
S0024	C0025	percentage of routine compliance	Defiant and oppositional behavior	compliance during home routine
S0024	C0026	percentage of routine compliance	Defiant and oppositional behavior	compliance during home routine
S0024	C0027	percentage of routine compliance	Defiant and oppositional behavior	compliance during home routine
S0026	C0028	Frequency of Aggressive Behavior	Aggressive behavior	aggressive behavior during classroom observations
S0026	C0029	Frequency of Aggressive Behavior	Aggressive behavior	aggressive behavior during classroom observations
S0026	C0030	Frequency of Aggressive Behavior	Aggressive behavior	aggressive behavior during classroom observations
S0028	C0031	Percentage of Intervals of Disruptive Behaviors	Irretable and angry behavior	disruptive behavior
S0028	C0032	Percentage of Intervals of Disruptive Behaviors	Defiant and oppositional behavior	disruptive behavior
S0028	C0033	Percentage of Intervals of Disruptive Behaviors	Defiant and oppositional behavior	disruptive behavior

S0028	C0034	Percentage of Intervals of Disruptive Behaviors	Irretable and angry behavior	disruptive behavior
S0028	C0035	Percentage of Intervals of Disruptive Behaviors	Defiant and oppositional behavior	disruptive behavior
S0028	C0036	Percentage of Intervals of Disruptive Behaviors	Defiant and oppositional behavior	disruptive behavior
S0037	C0039	disruptive behavior	Aggressive behavior	hostile tone
S0037	C0041	disruptive behavior	Aggressive behavior	hostile tone
S0037	C0042	disruptive behavior	Annoying others	hostile tone
S0040	C0044	percentage of compliant responses	Defiant and oppositional behavior	getting dressed; and tidying up
S0047	C0045	disruptive behavior	Defiant and oppositional behavior	disruptive behavior
S0047	C0046	disruptive behavior	Defiant and oppositional behavior	disruptive behavior
S0047	C0047	disruptive behavior	Defiant and oppositional behavior	disruptive behavior
S0048	C0048	externalizing behaviors	Defiant and oppositional behavior	externalizing behaviors
S0048	C0049	externalizing behaviors	Defiant and oppositional behavior	externalizing behaviors
S0048	C0050	externalizing behaviors	Defiant and oppositional behavior	externalizing behaviors
S0048	C0051	externalizing behaviors	Defiant and oppositional behavior	externalizing behaviors
S0048	C0052	externalizing behaviors	Defiant and oppositional behavior	externalizing behaviors
S0048	C0053	externalizing behaviors	Defiant and oppositional behavior	externalizing behaviors
S0048	C0054	externalizing behaviors	Defiant and oppositional behavior	externalizing behaviors
S0048	C0055	externalizing behaviors	Defiant and oppositional behavior	externalizing behaviors
S0048	C0056	externalizing behaviors	Defiant and oppositional behavior	externalizing behaviors
S0048	C0057	externalizing behaviors	Defiant and oppositional behavior	externalizing behaviors
S0048	C0058	externalizing behaviors	Defiant and oppositional behavior	externalizing behaviors
S0048	C0059	externalizing behaviors	Defiant and oppositional behavior	externalizing behaviors
S0050	C0060	problem behavior	Defiant and oppositional behavior	problem behavior
S0050	C0061	problem behavior	Defiant and oppositional behavior	problem behavior
S0050	C0062	problem behavior	Defiant and oppositional behavior	problem behavior

S0051	C0063	disruptive behavior	Defiant and oppositional behavior	intervals with problem behavior
S0062	C0064	Presenting Problems	Defiant and oppositional behavior	Presenting Problems
S0062	C0065	Presenting Problems	Aggressive behavior	Presenting Problems
S0062	C0066	Presenting Problems	Defiant and oppositional behavior	Presenting Problems
S0070	C0068	Compliance	Defiant and oppositional behavior	Compliance
S0070	C0069	Compliance	Defiant and oppositional behavior	Compliance
S0070	C0070	Compliance	Defiant and oppositional behavior	Compliance
S0071	C0071	Compliance	Defiant and oppositional behavior	Compliance
S0071	C0072	Compliance	Defiant and oppositional behavior	Compliance
S0071	C0073	Compliance	Defiant and oppositional behavior	Compliance
S0071	C0074	Compliance	Defiant and oppositional behavior	Compliance
S0071	C0075	Compliance	Defiant and oppositional behavior	Compliance
S0071	C0076	Compliance	Defiant and oppositional behavior	Compliance
S0071	C0077	Compliance	Defiant and oppositional behavior	Compliance
S0071	C0078	Compliance	Defiant and oppositional behavior	Compliance
S0075	C0091	disruptive behavior	Annoying others	frequency of inappropriate response; fight/no fight
S0084	C0092	disruptive verbal behavior	Annoying others	frequency of appropriate and inappropriate verbalization
S0084	C0093	disruptive verbal behavior	Annoying others	frequency of appropriate and inappropriate verbalization
S0096	C0100	problem behavior	Defiant and oppositional behavior	problem behavior
S0096	C0101	problem behavior	Defiant and oppositional behavior	problem behavior
S0096	C0102	problem behavior	Defiant and oppositional behavior	problem behavior
S0096	C0103	problem behavior	Annoying others	problem behavior
S0096	C0104	problem behavior	Defiant and oppositional behavior	problem behavior
S0096	C0105	problem behavior	Annoying others	problem behavior
S0101	C0106	frequency of aggression and defiance	Aggressive behavior	aggressive behavior and defiance
S0108	C0107	EBCI rating	Aggressive behavior	EBCI rating

S0113	C0109	students disruptive behavior during reading instruction	Other	students disruptive behavior during reading instruction
S0113	C0110	students disruptive behavior during reading instruction	Other	students disruptive behavior during reading instruction
S0113	C0111	students disruptive behavior during reading instruction	Other	students disruptive behavior during reading instruction
S0113	C0112	students disruptive behavior during reading instruction	Other	students disruptive behavior during reading instruction
S0113	C0113	students disruptive behavior during reading instruction	Other	students disruptive behavior during reading instruction
S0113	C0114	students disruptive behavior during reading instruction	Other	students disruptive behavior during reading instruction
S0116	C0115	disruptive behavior	Aggressive behavior	ECBI-scores
S0120	C0116	Average Frequency of Inappropriate Behavior	Aggressive behavior	Inappropriate Behavior
S0120	C0117	Average Frequency of Inappropriate Behavior	Aggressive behavior	Inappropriate Behavior
S0120	C0118	Average Frequency of Inappropriate Behavior	Aggressive behavior	Inappropriate Behavior
S0120	C0119	Average Frequency of Inappropriate Behavior	Aggressive behavior	Inappropriate Behavior
S0122	C0120	Interval of aggression and oppositional behavior	Aggressive behavior	Interval of aggression and oppositional behavior
S0122	C0121	Interval of aggression and oppositional behavior	Aggressive behavior	Interval of aggression and oppositional behavior
S0122	C0122	Interval of aggression and oppositional behavior	Aggressive behavior	Interval of aggression and oppositional behavior
S0123	C0126	observation of target behavior	Annoying others	inappropriate social behavior
S0123	C0128	observation of target behavior	Aggressive behavior	conflicts and behavioral problems
S0123	C0131	observation of target behavior	Aggressive behavior	hitting and pushing sibilings
S0123	C0132	observation of target behavior	Irretable and angry behavior	frustration behaviors
S0124	C0142	observation of negative interactions	Other	negative interactions
S0124	C0143	observation of negative interactions	Aggressive behavior	negative interactions
S0125	C0145	disruptive behavior	Annoying others	disruptive behavior
S0129	C0146	disruptive and off-task behavior	Defiant and oppositional behavior	disruptive and off-task behavior
S0129	C0147	disruptive and off-task behavior	Defiant and oppositional behavior	disruptive and off-task behavior
S0129	C0148	disruptive and off-task behavior	Defiant and oppositional behavior	disruptive and off-task behavior
S0129	C0149	disruptive and off-task behavior	Defiant and oppositional behavior	disruptive and off-task behavior
S0129	C0150	disruptive and off-task behavior	Defiant and oppositional behavior	disruptive and off-task behavior
S0129	C0151	disruptive and off-task behavior	Defiant and oppositional behavior	disruptive and off-task behavior
S0129	C0152	disruptive and off-task behavior	Defiant and oppositional behavior	disruptive and off-task behavior
S0129	C0153	disruptive and off-task behavior	Defiant and oppositional behavior	disruptive and off-task behavior

S0129	C0154	disruptive and off-task behavior	Defiant and oppositional behavior	disruptive and off-task behavior
S0129	C0155	disruptive and off-task behavior	Defiant and oppositional behavior	disruptive and off-task behavior
S0129	C0156	disruptive and off-task behavior	Defiant and oppositional behavior	disruptive and off-task behavior
S0134	C0157	Percentage of intervals with problem behavior	Aggressive behavior	Percentage of intervals with problem behavior
S0134	C0158	Percentage of intervals with problem behavior	Aggressive behavior	Percentage of intervals with problem behavior
S0136	C0160	percentage of compliant responses	Irretable and angry behavior	percentage of compliant responses
S0136	C0161	percentage of compliant responses	Irretable and angry behavior	percentage of compliant responses
S0136	C0162	percentage of compliant responses	Defiant and oppositional behavior	percentage of compliant responses
S0138	C0163	EBCI rating	Other	EBCI rating
S0138	C0164	EBCI rating	Other	EBCI rating
S0138	C0165	EBCI rating	Other	EBCI rating
S0147	C0323	EBCI rating	Defiant and oppositional behavior	EBCI rating
S0147	C0324	EBCI rating	Defiant and oppositional behavior	EBCI rating
S0153	C0166	Interval with Problem Behavior	Defiant and oppositional behavior	Interval with Problem Behavior
S0153	C0167	Interval with Problem Behavior	Defiant and oppositional behavior	Interval with Problem Behavior
S0153	C0168	Interval with Problem Behavior	Other	Interval with Problem Behavior
S0154	C0169	Interval with Problem Behavior	Defiant and oppositional behavior	Interval with Problem Behavior
S0154	C0170	Interval with Problem Behavior	Defiant and oppositional behavior	Interval with Problem Behavior
S0154	C0171	Interval with Problem Behavior	Irretable and angry behavior	Interval with Problem Behavior
S0154	C0172	Interval with Problem Behavior	Irretable and angry behavior	Interval with Problem Behavior
S0155	C0173	Number and Intensity of Aggressive Behavior	Aggressive behavior	Aggression
S0155	C0174	Number and Intensity of Aggressive Behavior	Aggressive behavior	Aggression
S0155	C0175	Number and Intensity of Aggressive Behavior	Aggressive behavior	Aggression
S0156	C0176	disruptive behavior	Defiant and oppositional behavior	Percentage of Inappropriate behavior
S0156	C0177	disruptive behavior	Defiant and oppositional behavior	Percentage of Inappropriate behavior
S0156	C0178	disruptive behavior	Defiant and oppositional behavior	Percentage of Inappropriate behavior
S0159	C0180	interactions	Irretable and angry behavior	number of interactions (inappropriate and appropriate) per hour

S0159	C0181	interactions	Irretable and angry behavior	number of interactions (inappropriate and appropriate) per hour
S0159	C0182	interactions	Aggressive behavior	number of interactions (inappropriate and appropriate) per hour
S0159	C0183	interactions	Irretable and angry behavior	number of interactions (inappropriate and appropriate) per hour
S0159	C0184	interactions	Aggressive behavior	number of interactions (inappropriate and appropriate) per hour
S0159	C0185	interactions	Aggressive behavior	number of interactions (inappropriate and appropriate) per hour
S0159	C0186	interactions	Aggressive behavior	number of interactions (inappropriate and appropriate) per hour
S0159	C0187	interactions	Irretable and angry behavior	number of interactions (inappropriate and appropriate) per hour
S0159	C0188	interactions	Irretable and angry behavior	number of interactions (inappropriate and appropriate) per hour
S0159	C0189	interactions	Other	number of interactions (inappropriate and appropriate) per hour
S0159	C0190	interactions	Irretable and angry behavior	number of interactions (inappropriate and appropriate) per hour
S0165	C0191	disruptive behavior	Aggressive behavior	ECBI-scores and Problem Scale T-scores
S0167	C0325	ECBI rating	Other	EBCI rating
S0170	C0192	Percentage of Maladaptive Behavior during structured play	Aggressive behavior	Percentage of Maladaptive Behavior during structured play
S0170	C0193	Percentage of Maladaptive Behavior during structured play	Aggressive behavior	Percentage of Maladaptive Behavior during structured play
S0170	C0194	Percentage of Maladaptive Behavior during structured play	Aggressive behavior	Percentage of Maladaptive Behavior during structured play
S0170	C0195	Percentage of Maladaptive Behavior during structured play	Aggressive behavior	Percentage of Maladaptive Behavior during structured play
S0173	C0196	Interval with respectful behavior	Defiant and oppositional behavior	Interval with respectful behavior
S0173	C0197	Interval with respectful behavior	Defiant and oppositional behavior	Interval with respectful behavior
S0173	C0198	Interval with respectful behavior	Defiant and oppositional behavior	Interval with respectful behavior
S0173	C0199	Interval with respectful behavior	Irretable and angry behavior	Interval with respectful behavior
S0180	C0200	occurance of target behavior	Defiant and oppositional behavior	occurance of target behavior
S0180	C0201	occurance of target behavior	Defiant and oppositional behavior	occurance of target behavior
S0192	C0205	Percentage of aggressive behavior	Aggressive behavior	physical and verbal aggression
S0192	C0206	Percentage of aggressive behavior	Aggressive behavior	physical and verbal aggression
S0192	C0207	Percentage of aggressive behavior	Aggressive behavior	physical and verbal aggression
S0192	C0208	Percentage of aggressive behavior	Defiant and oppositional behavior	physical and verbal aggression
S0197	C0211	Percentage of compliance	Defiant and oppositional behavior	Percentage of compliance
S0197	C0212	Percentage of compliance	Defiant and oppositional behavior	Percentage of compliance

S0197	C0213	Percentage of compliance	Defiant and oppositional behavior	Percentage of compliance
S0198	C0214	EBCI rating	Aggressive behavior	EBCI rating
S0198	C0215	EBCI rating	Aggressive behavior	EBCI rating
S0198	C0216	EBCI rating	Aggressive behavior	EBCI rating
S0202	C0217	disruptive behavior	Defiant and oppositional behavior	disruptive behavior
S0202	C0218	disruptive behavior	Irretable and angry behavior	disruptive behavior
S0202	C0219	disruptive behavior	Defiant and oppositional behavior	disruptive behavior
S0207	C0220	number of aggressive behaviors per 30 minutes	Aggressive behavior	number of aggressive behaviors per 30 minutes
S0207	C0221	number of aggressive behaviors per 30 minutes	Aggressive behavior	number of aggressive behaviors per 30 minutes
S0207	C0222	number of aggressive behaviors per 30 minutes	Aggressive behavior	number of aggressive behaviors per 30 minutes
S0207	C0224	number of aggressive behaviors per 30 minutes	Aggressive behavior	number of aggressive behaviors per 30 minutes
S0214	C0225	Percentage of intervals with disruptive behavior	Other	disruptive behavior
S0214	C0226	Percentage of intervals with disruptive behavior	Defiant and oppositional behavior	disruptive behavior
S0214	C0227	Percentage of intervals with disruptive behavior	Defiant and oppositional behavior	disruptive behavior
S0233	C0228	Percentage of intervals of aggression	Aggressive behavior	Aggression
S0233	C0229	Percentage of intervals of aggression	Aggressive behavior	Aggression
S0234	C0230	Frequency of target behavior	Defiant and oppositional behavior	Frequency of target behavior
S0234	C0231	Frequency of target behavior	Aggressive behavior	Frequency of target behavior
S0234	C0232	Frequency of target behavior	Aggressive behavior	Frequency of target behavior
S0246	C0234	Daily ourbursts	Irretable and angry behavior	Daily ourbursts
S0248	C0235	Percentage of Disruptive Intervals	Annoying others	Percentage of Disruptive Intervals
S0248	C0236	Percentage of Disruptive Intervals	Annoying others	Percentage of Disruptive Intervals
S0248	C0237	Percentage of Disruptive Intervals	Annoying others	Percentage of Disruptive Intervals
S0248	C0238	Percentage of Disruptive Intervals	Annoying others	Percentage of Disruptive Intervals
S0251	C0239	Child challenging behavior	Aggressive behavior	Child challenging behavior
S0251	C0240	Child challenging behavior	Defiant and oppositional behavior	Child challenging behavior
S0251	C0241	Child challenging behavior	Irretable and angry behavior	Child challenging behavior
S0253	C0243	cooperative behavior	Defiant and oppositional behavior	cooperative behavior

S0253	C0244	cooperative behavior	Defiant and oppositional behavior	cooperative behavior
S0253	C0245	cooperative behavior	Defiant and oppositional behavior	cooperative behavior
S0255	C0246	rate of aggressions per hour	Aggressive behavior	rate of aggressions per hour
S0255	C0247	rate of aggressions per hour	Aggressive behavior	rate of aggressions per hour
S0255	C0248	rate of aggressions per hour	Aggressive behavior	rate of aggressions per hour
S0255	C0249	rate of aggressions per hour	Aggressive behavior	rate of aggressions per hour
S0258	C0250	frequency of disruptive behavior	Defiant and oppositional behavior	disruptive behavior
S0258	C0251	frequency of disruptive behavior	Defiant and oppositional behavior	disruptive behavior
S0258	C0252	frequency of disruptive behavior	Aggressive behavior	disruptive behavior
S0258	C0253	frequency of disruptive behavior	Annoying others	disruptive behavior
S0262	C0255	Percentage of negative social engagement	Aggressive behavior	Percentage of negative social engagement
S0262	C0256	Percentage of negative social engagement	Aggressive behavior	Percentage of negative social engagement
S0262	C0257	Percentage of negative social engagement	Aggressive behavior	Percentage of negative social engagement
S0262	C0258	Percentage of negative social engagement	Aggressive behavior	Percentage of negative social engagement
S0262	C0259	Percentage of negative social engagement	Aggressive behavior	Percentage of negative social engagement
S0267	C0263	Frquency of aggression	Aggressive behavior	Aggression
S0267	C0264	Frquency of aggression	Aggressive behavior	Aggression
S0267	C0265	Frquency of aggression	Aggressive behavior	Aggression
S0282	C0266	Aggressive and rule-breaking behavior	Aggressive behavior	Aggressive and rule-breaking behavior
S0282	C0267	Aggressive and rule-breaking behavior	Aggressive behavior	Aggressive and rule-breaking behavior
S0282	C0268	Aggressive and rule-breaking behavior	Defiant and oppositional behavior	Aggressive and rule-breaking behavior
S0282	C0269	Aggressive and rule-breaking behavior	Aggressive behavior	Aggressive and rule-breaking behavior
S0293	C0270	Number of aggressive incidents	Aggressive behavior	Aggression
S0293	C0271	Number of aggressive incidents	Aggressive behavior	Aggression
S0294	C0273	Number of Problem Behaviors	Antisocial behavior	Bullying and fire setting
S0294	C0274	Number of Problem Behaviors	Aggressive behavior	Aggression and cruelty
S0294	C0275	Number of Problem Behaviors	Aggressive behavior	Aggresion and non-compliance
S0296	C0276	Frequency of verbal and physical aggression	Annoying others	verbal and physical aggression
S0296	C0277	Frequency of verbal and physical aggression	Annoying others	verbal and physical aggression

S0296	C0278	Frequency of verbal and physical aggression	Annoying others	verbal and physical aggression
S0300	C0279	Percentage of intervals of angry behavior or aggression	Aggressive behavior	Aggression
S0300	C0280	Percentage of intervals of angry behavior or aggression	Aggressive behavior	Aggression
S0300	C0281	Percentage of intervals of angry behavior or aggression	Aggressive behavior	Aggression
S0304	C0282	Frequency of problem behavior	Aggressive behavior	problem behavior
S0304	C0283	Frequency of problem behavior	Aggressive behavior	problem behavior
S0304	C0284	Frequency of problem behavior	Aggressive behavior	problem behavior
S0304	C0285	Frequency of problem behavior	Aggressive behavior	problem behavior
S0313	C0286	Percentage of calling out behavior and maintaining body boundaries	Aggressive behavior	Percentage of calling out behavior and maintaining body boundaries
S0313	C0287	Percentage of calling out behavior and maintaining body boundaries	Aggressive behavior	Percentage of calling out behavior and maintaining body boundaries
S0313	C0288	Percentage of calling out behavior and maintaining body boundaries	Aggressive behavior	Percentage of calling out behavior and maintaining body boundaries
S0313	C0289	Percentage of calling out behavior and maintaining body boundaries	Aggressive behavior	Percentage of calling out behavior and maintaining body boundaries
S0319	C352	Frequency of Behavior	Aggressive behavior	Frequency of Fighting, Hitting, Swearing
S0329	C0291	Percentage of Disruptive Behavior	Defiant and oppositional behavior	Percentage of Disruptive Behavior
S0329	C0292	Percentage of Disruptive Behavior	Defiant and oppositional behavior	Percentage of Disruptive Behavior
S0329	C0293	Percentage of Disruptive Behavior	Defiant and oppositional behavior	Percentage of Disruptive Behavior
S0329	C0294	Percentage of Disruptive Behavior	Defiant and oppositional behavior	Percentage of Disruptive Behavior
S0329	C0295	Percentage of Disruptive Behavior	Defiant and oppositional behavior	Percentage of Disruptive Behavior
S0329	C0296	Percentage of Disruptive Behavior	Defiant and oppositional behavior	Percentage of Disruptive Behavior
S0329	C0297	Percentage of Disruptive Behavior	Defiant and oppositional behavior	Percentage of Disruptive Behavior
S0329	C0298	Percentage of Disruptive Behavior	Defiant and oppositional behavior	Percentage of Disruptive Behavior
S0329	C0299	Percentage of Disruptive Behavior	Defiant and oppositional behavior	Percentage of Disruptive Behavior
S0331	C0300	Percentage appropriate interactions	Aggressive behavior	appropriate interactions
S0336	C0301	Percentage of intervals of destructive behavior	Aggressive behavior	destructive behavior
S0336	C0302	Percentage of intervals of destructive behavior	Aggressive behavior	destructive behavior
S0336	C0303	Percentage of intervals of destructive behavior	Aggressive behavior	destructive behavior
S0336	C0304	Percentage of intervals of destructive behavior	Aggressive behavior	destructive behavior

S0336	C0305	Percentage of intervals of destructive behavior	Aggressive behavior	destructive behavior
S0336	C0306	Percentage of intervals of destructive behavior	Aggressive behavior	destructive behavior
S0341	C0307	Average number of rule violations and teasing peers	Defiant and oppositional behavior	Average number of rule violations and teasing peers
S0342	C0308	disruptive behavior	Defiant and oppositional behavior	Percentage of inappropriate/ appropriate/ desirable behavior
S0342	C0309	disruptive behavior	Defiant and oppositional behavior	Percentage of inappropriate/ appropriate/ desirable behavior
S0345	C0310	Frequency of verbal abusiveness	Defiant and oppositional behavior	verbal aggression
S0345	C0311	Frequency of verbal abusiveness	Defiant and oppositional behavior	verbal aggression
S0345	C0312	Frequency of verbal abusiveness	Defiant and oppositional behavior	verbal aggression
S0345	C0313	Frequency of verbal abusiveness	Defiant and oppositional behavior	verbal aggression
S0345	C0314	Frequency of verbal abusiveness	Defiant and oppositional behavior	verbal aggression
S0345	C0315	Frequency of verbal abusiveness	Defiant and oppositional behavior	verbal aggression
S0358	C0316	disruptive behavior	Defiant and oppositional behavior	Percentage of intervals with yelling/ aggressive behavior/ instruction followed
S0359	C0317	frequency of disruptive behavior	Defiant and oppositional behavior	disruptive behavior
S0359	C0318	frequency of disruptive behavior	Defiant and oppositional behavior	disruptive behavior
S0359	C0319	frequency of disruptive behavior	Defiant and oppositional behavior	disruptive behavior
S0359	C0320	frequency of disruptive behavior	Defiant and oppositional behavior	disruptive behavior
S0359	C0321	frequency of disruptive behavior	Defiant and oppositional behavior	disruptive behavior
S0359	C0322	frequency of disruptive behavior	Defiant and oppositional behavior	disruptive behavior
S0362	C0341	Percentage of Treatment Fidelity and Student on-task behavior	Defiant and oppositional behavior	On-task behavior
S0362	C0342	Percentage of Treatment Fidelity and Student on-task behavior	Defiant and oppositional behavior	On-task behavior
S0362	C0343	Percentage of Treatment Fidelity and Student on-task behavior	Defiant and oppositional behavior	On-task behavior
S0363	C0344	Score on Teacher Assessment Report (TAR)	Other	problem behavior
S0363	C0345	Score on Teacher Assessment Report (TAR)	Other	problem behavior
S0363	C0346	Score on Teacher Assessment Report (TAR)	Other	problem behavior
S0363	C0347	Score on Teacher Assessment Report (TAR)	Other	problem behavior
S0363	C0348	Score on Teacher Assessment Report (TAR)	Other	problem behavior

S0363	C0349	Score on Teacher Assessment Report (TAR)	Other	problem behavior

Supplement #3L – Child Characteristics Part 10

study_id	child_id	total_observations	involvement_setting	outcome_var_type	reporter_type	mother_age	father_age
S0005	C0001	27	parents involved	Frequency	parents report	NR	NR
S0007	C0002	33	court involved	Score	parents report	NR	NR
S0007	C0003	45	court involved	Score	parents report	NR	NR
S0007	C0004	32	court involved	Score	parents report	NR	NR
S0007	C0005	33	court involved	Score	parents report	NR	NR
S0013	C0006	unclear	parents involved	Frequency	parents report	NR	NR
S0016	C0013	24	hospital unit; classroom	Percentage	observation	NR	NR
S0016	C0014	24	hospital unit; classroom	Percentage	observation	NR	NR
S0016	C0015	24	hospital unit; classroom	Percentage	observation	NR	NR
S0017	C0016	34	school involved	Percentage	observation	NR	NR
S0017	C0017	44	school involved	Percentage	observation	NR	NR
S0017	C0018	42	school involved	Percentage	observation	NR	NR
S0017	C0019	44	school involved	Percentage	observation	NR	NR
S0017	C0020	54	school involved	Percentage	observation	NR	NR
S0017	C0021	59	school involved	Percentage	observation	NR	NR
S0024	C0025	22	parents and school involved	Percentage	observation	NR	NR
S0024	C0026	24	foster family and school involved	Percentage	observation	NR	NR
S0024	C0027	12	parents and school involved	Percentage	observation	NR	NR
S0026	C0028	17	school involved	Frequency	observation	NR	NR
S0026	C0029	17	school involved	Frequency	observation	NR	NR
S0026	C0030	17	school involved	Frequency	observation	NR	NR
S0028	C0031	33	school involved	Percentage	teacher report	NR	NR
S0028	C0032	33	school involved	Percentage	teacher report	NR	NR
S0028	C0033	33	school involved	Percentage	teacher report	NR	NR
S0028	C0034	33	school involved	Percentage	teacher report	NR	NR
S0028	C0035	33	school involved	Percentage	teacher report	NR	NR

S0028	C0036	33	school involved	Percentage	teacher report	NR	NR
S0037	C0039	unclear	hospital unit	Frequency	observation	NR	NR
S0037	C0041	unclear	hospital unit	Frequency	observation	NR	NR
S0037	C0042	unclear	hospital unit	Frequency	observation	NR	NR
S0040	C0044	14	parents involved	Percentage	observation	NR	NR
S0047	C0045	15	school involved	Percentage	observation	NR	NR
S0047	C0046	12	school involved	Percentage	observation	NR	NR
S0047	C0047	16	school involved	Percentage	observation	NR	NR
S0048	C0048	unclear	school involved	Frequency	observation	NR	NR
S0048	C0049	unclear	school involved	Frequency	observation	NR	NR
S0048	C0050	unclear	school involved	Frequency	observation	NR	NR
S0048	C0051	unclear	school involved	Frequency	observation	NR	NR
S0048	C0052	unclear	school involved	Frequency	observation	NR	NR
S0048	C0053	unclear	school involved	Frequency	observation	NR	NR
S0048	C0054	unclear	school involved	Frequency	observation	NR	NR
S0048	C0055	unclear	school involved	Frequency	observation	NR	NR
S0048	C0056	unclear	school involved	Frequency	observation	NR	NR
S0048	C0057	unclear	school involved	Frequency	observation	NR	NR
S0048	C0058	unclear	school involved	Frequency	observation	NR	NR
S0048	C0059	unclear	school involved	Frequency	observation	NR	NR
S0050	C0060	16	school involved	Percentage	observation	NR	NR
S0050	C0061	21	school involved	Percentage	observation	NR	NR
S0050	C0062	42	school involved	Percentage	observation	NR	NR
S0051	C0063	51	school involved	Percentage	observations (f.a.); teacher report (social skills rating)	NR	NR
S0062	C0064	64	parents involved	Frequency	parents report	51	51
S0062	C0065	57	parents involved	Frequency	parents report	42	45
S0062	C0066	49	parents involved	Frequency	parents report	29	30
S0070	C0068	8	parents involved	Percentage	observation	NR	NR

S0070	C0069	9	parents involved	Percentage	observation	NR	NR
S0070	C0070	10	parents involved	Percentage	observation	NR	NR
S0071	C0071	7	parents involved	Percentage	observation	m = 41	NR
S0071	C0072	7	parents involved	Percentage	observation	m = 41	NR
S0071	C0073	7	parents involved	Percentage	observation	m = 41	NR
S0071	C0074	8	parents involved	Percentage	observation	m = 41	NR
S0071	C0075	8	parents involved	Percentage	observation	m = 41	NR
S0071	C0076	9	parents involved	Percentage	observation	m = 41	NR
S0071	C0077	9	parents involved	Percentage	observation	m = 41	NR
S0071	C0078	9	parents involved	Percentage	observation	m = 41	NR
S0075	C0091	45	school involved	Frequency	observations	NR	NR
S0084	C0092	40	school involved	Frequency	observations	NR	NR
S0084	C0093	40	school involved	Frequency	observations	NR	NR
S0096	C0100	14	school involved	Percentage	observation	NR	NR
S0096	C0101	17	school involved	Percentage	observation	NR	NR
S0096	C0102	26	school involved	Percentage	observation	NR	NR
S0096	C0103	20	school involved	Percentage	observation	NR	NR
S0096	C0104	15	school involved	Percentage	observation	NR	NR
S0096	C0105	13	school involved	Percentage	observation	NR	NR
S0101	C0106	unclear	parents involved	Frequency	observation	NR	NR
S0108	C0107	31	parents involved	Score	parents report	38	40
S0113	C0109	21	school involved	Percentage	observation	NR	NR
S0113	C0110	25	school involved	Percentage	observation	NR	NR
S0113	C0111	23	school involved	Percentage	observation	NR	NR
S0113	C0112	24	school involved	Percentage	observation	NR	NR
S0113	C0113	25	school involved	Percentage	observation	NR	NR
S0113	C0114	23	school involved	Percentage	observation	NR	NR
S0116	C0115	9	parents involved	Score	parent-report	NR	NR
S0120	C0116	52	parents involved	Frequency	observation	40	NR

S0120	C0117	52	parents involved	Frequency	observation	40	NR
S0120	C0118	62	parents involved	Frequency	observation	27	NR
S0120	C0119	62	parents involved	Frequency	observation	NR	NR
S0122	C0120	12	parents and school involved	Percentage	observation	22,0 - 48,0	NR
S0122	C0121	14	parents and school involved	Percentage	observation	22,0 - 48,0	NR
S0122	C0122	16	parents and school involved	Percentage	observation	22,0 - 48,0	NR
S0123	C0126	27	parents involved	Frequency	parents report	m = 36,3 (SD = 8,7)	m = 36,3 (SD = 8,7)
S0123	C0128	25	parents involved	Frequency	parents report	m = 36,3 (SD = 8,7)	m = 36,3 (SD = 8,7)
S0123	C0131	26	parents involved	Frequency	parents report	m = 36,3 (SD = 8,7)	m = 36,3 (SD = 8,7)
S0123	C0132	25	parents involved	Frequency	parents report	m = 36,3 (SD = 8,7)	m = 36,3 (SD = 8,7)
S0124	C0142	23	school involved	Percentage	observation	NR	NR
S0124	C0143	24	school involved	Percentage	observation	NR	NR
S0125	C0145	37	school involved	Percentage	observation	NR	NR
S0129	C0146	43	school involved	Percentage	observation	NR	NR
S0129	C0147	43	school involved	Percentage	observation	NR	NR
S0129	C0148	43	school involved	Percentage	observation	NR	NR
S0129	C0149	43	school involved	Percentage	observation	NR	NR
S0129	C0150	43	school involved	Percentage	observation	NR	NR
S0129	C0151	43	school involved	Percentage	observation	NR	NR
S0129	C0152	43	school involved	Percentage	observation	NR	NR
S0129	C0153	43	school involved	Percentage	observation	NR	NR
S0129	C0154	43	school involved	Percentage	observation	NR	NR
S0129	C0155	43	school involved	Percentage	observation	NR	NR
S0129	C0156	43	school involved	Percentage	observation	NR	NR
S0134	C0157	17	parents involved	Percentage	observation	NR	NR
S0134	C0158	29	parents involved	Percentage	observation	NR	NR
S0136	C0160	14	parents involved	Percentage	observation	35	34
S0136	C0161	19	parents involved	Percentage	observation	37	37

S0136	C0162	22	parents involved	Percentage	observation	40	41
S0138	C0163	40	parents involved	Score	parents report	33	NR
S0138	C0164	34	parents involved	Score	parents report	29	NR
S0138	C0165	36	parents involved	Score	parents report	44	NR
S0147	C0323	17	parents involved	Score	parent-report	m = 37,75	NR
S0147	C0324	19	parents involved	Score	parent-report	m = 37,75	NR
S0153	C0166	52	school involved	Percentage	observation	NR	NR
S0153	C0167	52	school involved	Percentage	observation	NR	NR
S0153	C0168	52	school involved	Percentage	observation	NR	NR
S0154	C0169	14	school involved	Percentage	observation	NR	NR
S0154	C0170	19	school involved	Percentage	observation	NR	NR
S0154	C0171	16	school involved	Percentage	observation	NR	NR
S0154	C0172	26	school involved	Percentage	observation	NR	NR
S0155	C0173	10	parents involved	Score	parents report	NR	NR
S0155	C0174	16	parents involved	Score	parents report	NR	NR
S0155	C0175	20	parents involved	Score	parents report	NR	NR
S0156	C0176	20	school involved	Percentage	observations	NR	NR
S0156	C0177	19	school involved	Percentage	observations	NR	NR
S0156	C0178	22	school involved	Percentage	observations	NR	NR
S0159	C0180	45	school involved	Frequency	observations	NR	NR
S0159	C0181	45	school involved	Frequency	observations	NR	NR
S0159	C0182	45	school involved	Frequency	observations	NR	NR
S0159	C0183	31	school involved	Frequency	observations	NR	NR
S0159	C0184	21	school involved	Frequency	observations	NR	NR
S0159	C0185	23	school involved	Frequency	observations	NR	NR
S0159	C0186	16	school involved	Frequency	observations	NR	NR
S0159	C0187	11	school involved	Frequency	observations	NR	NR
S0159	C0188	20	school involved	Frequency	observations	NR	NR
S0159	C0189	17	school involved	Frequency	observations	NR	NR

S0159	C0190	12	school involved	Frequency	observations	NR	NR
S0165	C0191	23	parents involved	Score	parent-report	NR	NR
S0167	C0325	7	parents involved	Score	parent-report	NR	m = 41
S0170	C0192	48	school involved	Percentage	observation	NR	NR
S0170	C0193	72	school involved	Percentage	observation	NR	NR
S0170	C0194	72	school involved	Percentage	observation	NR	NR
S0170	C0195	73	school involved	Percentage	observation	NR	NR
S0173	C0196	12	school involved	Percentage	observation	NR	NR
S0173	C0197	11	school involved	Percentage	observation	NR	NR
S0173	C0198	13	school involved	Percentage	observation	NR	NR
S0173	C0199	15	school involved	Percentage	observation	NR	NR
S0180	C0200	68	school involved	Percentage	observation	NR	NR
S0180	C0201	50	school involved	Percentage	observation	NR	NR
S0192	C0205	unclear	treatment facility involved	Percentage	observation	NR	NR
S0192	C0206	unclear	treatment facility involved	Percentage	observation	NR	NR
S0192	C0207	unclear	treatment facility involved	Percentage	observation	NR	NR
S0192	C0208	unclear	treatment facility involved	Percentage	observation	NR	NR
S0197	C0211	37	school involved	Percentage	observation	NR	NR
S0197	C0212	25	school involved	Percentage	observation	NR	NR
S0197	C0213	36	school involved	Percentage	observation	NR	NR
S0198	C0214	17	parents involved	Score	parents report	NR	38
S0198	C0215	24	parents involved	Score	parents report	39	NR
S0198	C0216	20	parents involved	Score	parents report	25	NR
S0202	C0217	10	school involved	Percentage	teacher report	NR	NR
S0202	C0218	10	school involved	Percentage	teacher report	NR	NR
S0202	C0219	10	school involved	Percentage	teacher report	NR	NR
S0207	C0220	21	parents and school involved	Frequency	observation	NR	NR
S0207	C0221	27	parents and school involved	Frequency	observation	NR	NR
S0207	C0222	18	parents and school involved	Frequency	observation	NR	NR

S0207	C0224	12	parents and school involved	Frequency	observation	NR	NR
S0214	C0225	25	school involved	Percentage	observation	NR	NR
S0214	C0226	25	school involved	Percentage	observation	NR	NR
S0214	C0227	20	school involved	Percentage	observation	NR	NR
S0233	C0228	78	school involved	Percentage	observation	NR	NR
S0233	C0229	78	school involved	Percentage	observation	NR	NR
S0234	C0230	41	caregiver and school involved	Frequency	observation	NR	NR
S0234	C0231	41	caregiver and school involved	Frequency	observation	NR	NR
S0234	C0232	36	caregiver and school involved	Frequency	observation	NR	35
S0246	C0234	16	parents involved	Frequency	parents report	NR	NR
S0248	C0235	25	school involved	Percentage	observation	NR	NR
S0248	C0236	24	school involved	Percentage	observation	NR	NR
S0248	C0237	36	school involved	Percentage	observation	NR	NR
S0248	C0238	33	school involved	Percentage	observation	NR	NR
S0251	C0239	14	parents involved	Percentage	parents report	34	NR
S0251	C0240	23	parents involved	Percentage	parents report	31	NR
S0251	C0241	22	parents involved	Percentage	parents report	34	NR
S0253	C0243	35	school involved	Percentage	observation	NR	NR
S0253	C0244	35	school involved	Percentage	observation	NR	NR
S0253	C0245	35	school involved	Percentage	observation	NR	NR
S0255	C0246	11	school involved	Percentage	teacher report	NR	NR
S0255	C0247	28	school involved	Percentage	teacher report	NR	NR
S0255	C0248	34	school involved	Percentage	teacher report	NR	NR
S0255	C0249	32	school involved	Percentage	teacher report	NR	NR
S0258	C0250	15	parents involved	Frequency	parents report	m = 32 (SD = 3)	NR
S0258	C0251	12	parents involved	Frequency	parents report	m = 32 (SD = 3)	NR
S0258	C0252	12	parents involved	Frequency	parents report	m = 32 (SD = 3)	NR
S0258	C0253	12	parents involved	Frequency	parents report	m = 32 (SD = 3)	NR

S0262	C0255	unclear	school involved	Percentage	observation	NR	NR
S0262	C0256	unclear	school involved	Percentage	observation	NR	NR
S0262	C0257	unclear	school involved	Percentage	observation	NR	NR
S0262	C0258	unclear	school involved	Percentage	observation	NR	NR
S0262	C0259	unclear	school involved	Percentage	observation	NR	NR
S0267	C0263	23	school involved	Frequency	observation	NR	NR
S0267	C0264	22	school involved	Frequency	observation	NR	NR
S0267	C0265	22	school involved	Frequency	observation	NR	NR
S0282	C0266	17	parents involved	Score	parents report	37	42
S0282	C0267	17	parents involved	Score	parents report	30	39
S0282	C0268	17	parents involved	Score	parents report	32	38
S0282	C0269	17	parents involved	Score	parents report	40	52
S0293	C0270	20	parents involved	Frequency	parents report	NR	NR
S0293	C0271	26	parents involved	Frequency	parents report	NR	NR
S0294	C0273	33	school involved	Frequency	teacher report	NR	NR
S0294	C0274	36	school involved	Frequency	teacher report	NR	NR
S0294	C0275	40	school involved	Frequency	teacher report	NR	NR
S0296	C0276	35	parents involved	Frequency	parents report	NR	NR
S0296	C0277	35	parents involved	Frequency	parents report	NR	NR
S0296	C0278	35	parents involved	Frequency	parents report	NR	NR
S0300	C0279	28	school involved	Percentage	observation	NR	NR
S0300	C0280	27	school involved	Percentage	observation	NR	NR
S0300	C0281	26	school involved	Percentage	observation	NR	NR
S0304	C0282	60	school involved	Frequency	observation	NR	NR
S0304	C0283	60	school involved	Frequency	observation	NR	NR
S0304	C0284	60	school involved	Frequency	observation	NR	NR
S0304	C0285	60	school involved	Frequency	observation	NR	NR
S0313	C0286	28	school involved	Percentage	observation	NR	NR
S0313	C0287	29	school involved	Percentage	observation	NR	NR

S0313	C0288	28	school involved	Percentage	observation	NR	NR
S0313	C0289	28	school involved	Percentage	observation	NR	NR
S0319	C352	29	treatment facility involved	Frequency	observation	NR	NR
S0329	C0291	20	school involved	Percentage	observation	NR	NR
S0329	C0292	20	school involved	Percentage	observation	NR	NR
S0329	C0293	20	school involved	Percentage	observation	NR	NR
S0329	C0294	20	school involved	Percentage	observation	NR	NR
S0329	C0295	20	school involved	Percentage	observation	NR	NR
S0329	C0296	20	school involved	Percentage	observation	NR	NR
S0329	C0297	20	school involved	Percentage	observation	NR	NR
S0329	C0298	20	school involved	Percentage	observation	NR	NR
S0329	C0299	20	school involved	Percentage	observation	NR	NR
S0331	C0300	23	no involvment	Percentage	observation	NR	NR
S0336	C0301	30	parents involved	Percentage	observation	NR	NR
S0336	C0302	25	parents involved	Percentage	observation	NR	NR
S0336	C0303	27	parents involved	Percentage	observation	NR	NR
S0336	C0304	11	parents involved	Percentage	observation	NR	NR
S0336	C0305	27	parents involved	Percentage	observation	NR	NR
S0336	C0306	16	parents involved	Percentage	observation	NR	NR
S0341	C0307	8	parents and school involved	Score	observation	NR	NR
S0342	C0308	60	school involved	Percentage	observations	NR	NR
S0342	C0309	60	school involved	Percentage	observations	NR	NR
S0345	C0310	unclear	parents involved	Frequency	parents report	NR	NR
S0345	C0311	unclear	parents involved	Frequency	parents report	NR	NR
S0345	C0312	unclear	parents involved	Frequency	parents report	NR	NR
S0345	C0313	unclear	parents involved	Frequency	parents report	NR	NR
S0345	C0314	unclear	parents involved	Frequency	parents report	NR	NR
S0345	C0315	unclear	parents involved	Frequency	parents report	NR	NR
S0358	C0316	28	parents involved	Percentage	observations	NR	NR

S0359	C0317	13	parents and school involved	Frequency	observation	NR	NR
S0359	C0318	13	parents and school involved	Frequency	observation	NR	NR
S0359	C0319	13	parents and school involved	Frequency	observation	NR	NR
S0359	C0320	13	parents and school involved	Frequency	observation	NR	NR
S0359	C0321	13	parents and school involved	Frequency	observation	NR	NR
S0359	C0322	13	parents and school involved	Frequency	observation	NR	NR
S0362	C0341	13	school involved	Percentage	observation	NR	NR
S0362	C0342	12	school involved	Percentage	observation	NR	NR
S0362	C0343	14	school involved	Percentage	observation	NR	NR
S0363	C0344	9	school involved	Score	teacher report	NR	NR
S0363	C0345	9	school involved	Score	teacher report	NR	NR
S0363	C0346	9	school involved	Score	teacher report	NR	NR
S0363	C0347	8	school involved	Score	teacher report	NR	NR
S0363	C0348	8	school involved	Score	teacher report	NR	NR
S0363	C0349	7	school involved	Score	teacher report	NR	NR

Supplement #3M – Child Characteristics Part 11

study _id	child_ id	family_involvement
S0005	C0001	Parental sessions; Parental training, Home Observatiosand Record-Keeping; Behavior Reinforcment; Feedback
S0007	C0002	Mother filled out EBS scales
S0007	C0003	Mother filled out EBS scales
S0007	C0004	Mother filled out EBS scales
S0007	C0005	Mother filled out EBS scales
S0013	C0006	Parents trained in PCIT and ACT, recorded behaviors daily
S0016	C0013	NR
S0016	C0014	NR
S0016	C0015	NR
S0017	C0016	NR
S0017	C0017	NR
S0017	C0018	NR
S0017	C0019	NR
S0017	C0020	NR
S0017	C0021	NR
S0024	C0025	Families actively participated in developing and implementing behavioral interventions alongside teachers and a pediatric mental health consultant. Parents were involved in identifying target behaviors, setting goals, and applying interventions at home to ensure consistency across settings. Regular communication and collaboration between families, educators, and the consultant facilitated the ongoing evaluation and adjustment of intervention strategies
S0024	C0026	Families actively participated in developing and implementing behavioral interventions alongside teachers and a pediatric mental health consultant. Parents were involved in identifying target behaviors, setting goals, and applying interventions at home to ensure consistency across settings. Regular communication and collaboration between families, educators, and the consultant facilitated the ongoing evaluation and adjustment of intervention strategies
S0024	C0027	Families actively participated in developing and implementing behavioral interventions alongside teachers and a pediatric mental health consultant. Parents were involved in identifying target behaviors, setting goals, and applying interventions at home to ensure consistency across settings. Regular communication and collaboration between families, educators, and the consultant facilitated the ongoing evaluation and adjustment of intervention strategies
S0026	C0028	NR
S0026	C0029	NR
S0026	C0030	NR
S0028	C0031	NR
S0028	C0032	NR

S0028	C0033	NR
S0028	C0034	NR
S0028	C0035	NR
S0028	C0036	NR
S0037	C0039	NR
S0037	C0041	NR
S0037	C0042	NR
S0040	C0044	The mother was trained as a participant-observer and kept detailed records in ABC diaries
S0047	C0045	NR
S0047	C0046	NR
S0047	C0047	NR
S0048	C0048	NR
S0048	C0049	NR
S0048	C0050	NR
S0048	C0051	NR
S0048	C0052	NR
S0048	C0053	NR
S0048	C0054	NR
S0048	C0055	NR
S0048	C0056	NR
S0048	C0057	NR
S0048	C0058	NR
S0048	C0059	NR
S0050	C0060	parents completed pre and post assessment and feedback on social validity
S0050	C0061	parents completed pre and post assessment and feedback on social validity
S0050	C0062	parents completed pre and post assessment and feedback on social validity
S0051	C0063	daily note to parents to encourage home-school collaboration and consistency
S0062	C0064	Joint sessions with child; setting goals together: comunication and interactions; Daily reports; providing feedback
S0062	C0065	Joint sessions with child; setting goals together: comunication and interactions; Daily reports; providing feedback

S0062	C0066	Joint sessions with child; setting goals together: comunication and interactions; Daily reports; providing feedback
S0070	C0068	didactic instuctions for parents
S0070	C0069	didactic instuctions for parents
S0070	C0070	didactic instuctions for parents
S0071	C0071	parents got parenting training
S0071	C0072	parents got parenting training
S0071	C0073	parents got parenting training
S0071	C0074	parents got parenting training
S0071	C0075	parents got parenting training
S0071	C0076	parents got parenting training
S0071	C0077	parents got parenting training
S0071	C0078	parents got parenting training
S0075	C0091	NR
S0084	C0092	NR
S0084	C0093	NR
S0096	C0100	NR
S0096	C0101	NR
S0096	C0102	NR
S0096	C0103	NR
S0096	C0104	NR
S0096	C0105	NR
S0101	C0106	parents were actively involved in the intervention by participating in therapy sessions, learning and applying behavior management techniques
S0108	C0107	mother was activily involved in attending weekly sessions; practicing learning skills; completing homework; father did not participate in intervention
S0113	C0109	NR
S0113	C0110	NR
S0113	C0111	NR
S0113	C0112	NR
S0113	C0113	NR
S0113	C0114	NR

S0116	C0115	psychoeducation; Child Directed Interaction (CDI) training; Parent-Directed Interaction (PDI); Emotional-Regulation Strategies; In-Home Coaching; Reinforcment Systems
S0120	C0116	parental training; home visits; interactions
S0120	C0117	parental training; home visits; interactions
S0120	C0118	parental training; home visits; interactions
S0120	C0119	parental training; home visits; interactions
S0122	C0120	mothers participated in group workshops and individual sessions
S0122	C0121	mothers participated in group workshops and individual sessions
S0122	C0122	mothers participated in group workshops and individual sessions
S0123	C0126	participated in sessions; evaluated training; engaged in the intervention
S0123	C0128	participated in sessions; evaluated training; engaged in the intervention
S0123	C0131	participated in sessions; evaluated training; engaged in the intervention
S0123	C0132	participated in sessions; evaluated training; engaged in the intervention
S0124	C0142	NR
S0124	C0143	NR
S0125	C0145	NR
S0129	C0146	NR
S0129	C0147	NR
S0129	C0148	NR
S0129	C0149	NR
S0129	C0150	NR
S0129	C0151	NR
S0129	C0152	NR
S0129	C0153	NR
S0129	C0154	NR
S0129	C0155	NR
S0129	C0156	NR
S0134	C0157	NR
S0134	C0158	NR

S0136	C0160	NR
S0136	C0161	NR
S0136	C0162	NR
S0138	C0163	all parents actively engaged in the intervention
S0138	C0164	all parents actively engaged in the intervention
S0138	C0165	all parents actively engaged in the intervention
S0147	C0323	parents were actively involved in the intervention by participating in therapy sessions, learning and applying behavior management techniques, and practicing at home
S0147	C0324	parents were actively involved in the intervention by participating in therapy sessions, learning and applying behavior management techniques, and practicing at home
S0153	C0166	involved in checking and signing the daily report card
S0153	C0167	involved in checking and signing the daily report card
S0153	C0168	involved in checking and signing the daily report card
S0154	C0169	involved in checking and signing the daily report card
S0154	C0170	involved in checking and signing the daily report card
S0154	C0171	involved in checking and signing the daily report card
S0154	C0172	involved in checking and signing the daily report card
S0155	C0173	actively involved in study; responsible for observing and recording childs behavior
S0155	C0174	actively involved in study; responsible for observing and recording childs behavior
S0155	C0175	actively involved in study; responsible for observing and recording childs behavior
S0156	C0176	NR
S0156	C0177	NR
S0156	C0178	NR
S0159	C0180	NR
S0159	C0181	NR
S0159	C0182	NR
S0159	C0183	NR
S0159	C0184	NR
S0159	C0185	NR
S0159	C0186	NR

S0159	C0187	NR
S0159	C0188	NR
S0159	C0189	NR
S0159	C0190	NR
S0165	C0191	actively involved in the therapy sessions
S0167	C0325	parents were actively involved in the intervention by participating in therapy sessions, learning and applying behavior management techniques, and practicing at home
S0170	C0192	NR
S0170	C0193	NR
S0170	C0194	NR
S0170	C0195	NR
S0173	C0196	NR
S0173	C0197	NR
S0173	C0198	NR
S0173	C0199	NR
S0180	C0200	NR
S0180	C0201	NR
S0192	C0205	NR
S0192	C0206	NR
S0192	C0207	NR
S0192	C0208	NR
S0197	C0211	NR
S0197	C0212	NR
S0197	C0213	NR
S0198	C0214	NR
S0198	C0215	NR
S0198	C0216	NR
S0202	C0217	NR
S0202	C0218	NR

S0202	C0219	NR
S0207	C0220	NR
S0207	C0221	NR
S0207	C0222	NR
S0207	C0224	NR
S0214	C0225	NR
S0214	C0226	NR
S0214	C0227	NR
S0233	C0228	NR
S0233	C0229	NR
S0234	C0230	caregivers were involved in the intervention process, but their participation varied. For instance, some attended meetings via phone due to scheduling conflicts
S0234	C0231	caregivers were involved in the intervention process, but their participation varied. For instance, some attended meetings via phone due to scheduling conflicts
S0234	C0232	caregivers were involved in the intervention process, but their participation varied. For instance, some attended meetings via phone due to scheduling conflicts
S0246	C0234	active participation in therapy; daily monitoring; supporting the child's progress
S0248	C0235	NR
S0248	C0236	NR
S0248	C0237	NR
S0248	C0238	NR
S0251	C0239	mothers provided treatment to children
S0251	C0240	mothers provided treatment to children
S0251	C0241	mothers provided treatment to children
S0253	C0243	NR
S0253	C0244	NR
S0253	C0245	NR
S0255	C0246	NR
S0255	C0247	NR
S0255	C0248	NR
S0255	C0249	NR
S0258	C0250	NR

S0258	C0251	NR
S0258	C0252	NR
S0258	C0253	NR
S0262	C0255	NR
S0262	C0256	NR
S0262	C0257	NR
S0262	C0258	NR
S0262	C0259	NR
S0267	C0263	NR
S0267	C0264	NR
S0267	C0265	NR
S0282	C0266	parents were actively involved in the intervention, participating in the theraplay sessions with their children and receiving additional parenting training aimed at improving the parent-child relationship and supporting the child's emotional needs
S0282	C0267	parents were actively involved in the intervention, participating in the theraplay sessions with their children and receiving additional parenting training aimed at improving the parent-child relationship and supporting the child's emotional needs
S0282	C0268	parents were actively involved in the intervention, participating in the theraplay sessions with their children and receiving additional parenting training aimed at improving the parent-child relationship and supporting the child's emotional needs
S0282	C0269	parents were actively involved in the intervention, participating in the theraplay sessions with their children and receiving additional parenting training aimed at improving the parent-child relationship and supporting the child's emotional needs
S0293	C0270	mothers were actively involved as they were responsible for implementing the mindfulness-based strategy with their children at home and during community outings
S0293	C0271	mothers were actively involved as they were responsible for implementing the mindfulness-based strategy with their children at home and during community outings
S0294	C0273	NR
S0294	C0274	NR
S0294	C0275	NR
S0296	C0276	involved in the initial training but also in the daily implementation, monitoring, and reinforcement of the mindfulness practice
S0296	C0277	involved in the initial training but also in the daily implementation, monitoring, and reinforcement of the mindfulness practice
S0296	C0278	involved in the initial training but also in the daily implementation, monitoring, and reinforcement of the mindfulness practice
S0300	C0279	NR
S0300	C0280	NR
S0300	C0281	NR
S0304	C0282	NR

S0304	C0283	NR
S0304	C0284	NR
S0304	C0285	NR
S0313	C0286	there were numerous attempts to ivolve parents into therapy but it was largely unsuccessful
S0313	C0287	there were numerous attempts to ivolve parents into therapy but it was largely unsuccessful
S0313	C0288	there were numerous attempts to ivolve parents into therapy but it was largely unsuccessful
S0313	C0289	there were numerous attempts to ivolve parents into therapy but it was largely unsuccessful
S0319	C352	history of physical abuse, neglect and abondonment
S0329	C0291	NR
S0329	C0292	NR
S0329	C0293	NR
S0329	C0294	NR
S0329	C0295	NR
S0329	C0296	NR
S0329	C0297	NR
S0329	C0298	NR
S0329	C0299	NR
S0331	C0300	Bob's mother suffered from alcoholism and was physically abusive; his father was passive and dominated by his wife
S0336	C0301	parents were treatment provider and coached via telehealth from clinician
S0336	C0302	parents were treatment provider and coached via telehealth from clinician
S0336	C0303	parents were treatment provider and coached via telehealth from clinician
S0336	C0304	parents were treatment provider and coached via telehealth from clinician
S0336	C0305	parents were treatment provider and coached via telehealth from clinician
S0336	C0306	parents were treatment provider and coached via telehealth from clinician
S0341	C0307	Child has history of taking Focalin XR; Intuniv; adderall; and Vyvanse but all medication was discontinued before the start of intervention
S0342	C0308	NR
S0342	C0309	NR
S0345	C0310	active participation in contracting and data reporting
S0345	C0311	active participation in contracting and data reporting

S0345	C0312	active participation in contracting and data reporting
S0345	C0313	active participation in contracting and data reporting
S0345	C0314	active participation in contracting and data reporting
S0345	C0315	active participation in contracting and data reporting
S0358	C0316	actively involved
S0359	C0317	teacher communicated with parents via WeChat (messenger App) to coordinate the reinforcment of behaviors at home
S0359	C0318	teacher communicated with parents via WeChat (messenger App) to coordinate the reinforcment of behaviors at home
S0359	C0319	teacher communicated with parents via WeChat (messenger App) to coordinate the reinforcment of behaviors at home
S0359	C0320	teacher communicated with parents via WeChat (messenger App) to coordinate the reinforcment of behaviors at home
S0359	C0321	teacher communicated with parents via WeChat (messenger App) to coordinate the reinforcment of behaviors at home
S0359	C0322	teacher communicated with parents via WeChat (messenger App) to coordinate the reinforcment of behaviors at home
S0362	C0341	Consent was obtained from each target student's parent or guardian before the children participated
S0362	C0342	Consent was obtained from each target student's parent or guardian before the children participated
S0362	C0343	Consent was obtained from each target student's parent or guardian before the children participated
S0363	C0344	Consent was obtained from each target student's parent or guardian before the children participated
S0363	C0345	Consent was obtained from each target student's parent or guardian before the children participated
S0363	C0346	Consent was obtained from each target student's parent or guardian before the children participated
S0363	C0347	Consent was obtained from each target student's parent or guardian before the children participated
S0363	C0348	Consent was obtained from each target student's parent or guardian before the children participated
S0363	C0349	Consent was obtained from each target student's parent or guardian before the children participated

Supplement #3N – Child Characteristics Part 12

study_id	child_id	Comments
S0005	C0001	
S0007	C0002	child commited crime previously: assult
S0007	C0003	child commited crime previously: destruction of property
S0007	C0004	child commited crime previously: destruction of property
S0007	C0005	child commited crime previously: solicitation of drugs
S0013	C0006	
S0016	C0013	
S0016	C0014	
S0016	C0015	
S0017	C0016	
S0017	C0017	
S0017	C0018	
S0017	C0019	
S0017	C0020	
S0017	C0021	
S0024	C0025	
S0024	C0026	
S0024	C0027	
S0026	C0028	
S0026	C0029	
S0026	C0030	
S0028	C0031	
S0028	C0032	
S0028	C0033	
S0028	C0034	
S0028	C0035	
S0028	C0036	
S0037	C0039	

S0037	C0041	
S0037	C0042	
S0040	C0044	
S0047	C0045	This study may have been excluded during data extraction
S0047	C0046	This study may have been excluded during data extraction
S0047	C0047	This study may have been excluded during data extraction
S0048	C0048	The research team identified participants from another study were these students were not responsive to the effects of the Strong Start curriculum
S0048	C0049	The research team identified participants from another study were these students were not responsive to the effects of the Strong Start curriculum
S0048	C0050	The research team identified participants from another study were these students were not responsive to the effects of the Strong Start curriculum
S0048	C0051	The research team identified participants from another study were these students were not responsive to the effects of the Strong Start curriculum
S0048	C0052	The research team identified participants from another study were these students were not responsive to the effects of the Strong Start curriculum
S0048	C0053	The research team identified participants from another study were these students were not responsive to the effects of the Strong Start curriculum
S0048	C0054	The research team identified participants from another study were these students were not responsive to the effects of the Strong Start curriculum
S0048	C0055	The research team identified participants from another study were these students were not responsive to the effects of the Strong Start curriculum
S0048	C0056	The research team identified participants from another study were these students were not responsive to the effects of the Strong Start curriculum
S0048	C0057	The research team identified participants from another study were these students were not responsive to the effects of the Strong Start curriculum
S0048	C0058	The research team identified participants from another study were these students were not responsive to the effects of the Strong Start curriculum
S0048	C0059	The research team identified participants from another study were these students were not responsive to the effects of the Strong Start curriculum
S0050	C0060	
S0050	C0061	
S0050	C0062	
S0051	C0063	
S0062	C0064	
S0062	C0065	
S0062	C0066	
S0070	C0068	
S0070	C0069	
S0070	C0070	
S0071	C0071	
S0071	C0072	
S0071	C0073	

S0071	C0074	
S0071	C0075	
S0071	C0076	
S0071	C0077	
S0071	C0078	
S0075	C0091	
S0084	C0092	
S0084	C0093	
S0096	C0100	
S0096	C0101	
S0096	C0102	
S0096	C0103	
S0096	C0104	
S0096	C0105	
S0101	C0106	
S0108	C0107	
S0113	C0109	
S0113	C0110	
S0113	C0111	
S0113	C0112	
S0113	C0113	
S0113	C0114	
S0116	C0115	Both parents have a history of depression; Mother is currently on antidepressants
S0120	C0116	Amy and John are twins
S0120	C0117	Amy and John are twins
S0120	C0118	
S0120	C0119	
S0122	C0120	low SES
S0122	C0121	low SES
S0122	C0122	low SES
S0123	C0126	

S0123	C0128	
S0123	C0131	
S0123	C0132	
S0124	C0142	Child mainly has internalizing problems
S0124	C0143	
S0125	C0145	
S0129	C0146	
S0129	C0147	
S0129	C0148	
S0129	C0149	
S0129	C0150	
S0129	C0151	
S0129	C0152	
S0129	C0153	
S0129	C0154	
S0129	C0155	
S0129	C0156	
S0134	C0157	
S0134	C0158	
S0136	C0160	
S0136	C0161	
S0136	C0162	
S0138	C0163	
S0138	C0164	
S0138	C0165	
S0147	C0323	
S0147	C0324	
S0153	C0166	
S0153	C0167	
S0153	C0168	
S0154	C0169	

S0154	C0170	
S0154	C0171	
S0154	C0172	
S0155	C0173	
S0155	C0174	
S0155	C0175	
S0156	C0176	
S0156	C0177	
S0156	C0178	
S0159	C0180	
S0159	C0181	
S0159	C0182	
S0159	C0183	
S0159	C0184	
S0159	C0185	
S0159	C0186	
S0159	C0187	
S0159	C0188	
S0159	C0189	
S0159	C0190	
S0165	C0191	
S0167	C0325	Dyad 1 withdrew from the study after three weeks of baseline and four weeks of intervention due to stress with scheduling and other personal matters
S0170	C0192	
S0170	C0193	
S0170	C0194	
S0170	C0195	
S0173	C0196	
S0173	C0197	
S0173	C0198	
S0173	C0199	
S0180	C0200	

S0180	C0201	
S0192	C0205	
S0192	C0206	
S0192	C0207	
S0192	C0208	
S0197	C0211	
S0197	C0212	
S0197	C0213	
S0198	C0214	
S0198	C0215	
S0198	C0216	
S0202	C0217	
S0202	C0218	
S0202	C0219	
S0207	C0220	
S0207	C0221	
S0207	C0222	
S0207	C0224	
S0214	C0225	
S0214	C0226	
S0214	C0227	
S0233	C0228	
S0233	C0229	
S0234	C0230	
S0234	C0231	
S0234	C0232	
S0246	C0234	
S0248	C0235	
S0248	C0236	
S0248	C0237	
S0248	C0238	

S0251	C0239	
S0251	C0240	
S0251	C0241	
S0253	C0243	
S0253	C0244	
S0253	C0245	
S0255	C0246	
S0255	C0247	
S0255	C0248	
S0255	C0249	
S0258	C0250	
S0258	C0251	
S0258	C0252	
S0258	C0253	
S0262	C0255	
S0262	C0256	
S0262	C0257	
S0262	C0258	
S0262	C0259	
S0267	C0263	
S0267	C0264	
S0267	C0265	
S0282	C0266	All participants lost a sibling due to cancer
S0282	C0267	All participants lost a sibling due to cancer
S0282	C0268	All participants lost a sibling due to cancer
S0282	C0269	All participants lost a sibling due to cancer
S0293	C0270	
S0293	C0271	
S0294	C0273	
S0294	C0274	
S0294	C0275	

S0296	C0276	
S0296	C0277	
S0296	C0278	
S0300	C0279	
S0300	C0280	
S0300	C0281	
S0304	C0282	
S0304	C0283	
S0304	C0284	
S0304	C0285	
S0313	C0286	low SES
S0313	C0287	low SES
S0313	C0288	low SES
S0313	C0289	low SES
S0319	C352	
S0329	C0291	
S0329	C0292	
S0329	C0293	
S0329	C0294	
S0329	C0295	
S0329	C0296	
S0329	C0297	
S0329	C0298	
S0329	C0299	
S0331	C0300	
S0336	C0301	
S0336	C0302	
S0336	C0303	
S0336	C0304	
S0336	C0305	
S0336	C0306	

S0341	C0307	
S0342	C0308	
S0342	C0309	
S0345	C0310	
S0345	C0311	
S0345	C0312	
S0345	C0313	
S0345	C0314	
S0345	C0315	
S0358	C0316	
S0359	C0317	
S0359	C0318	
S0359	C0319	
S0359	C0320	
S0359	C0321	
S0359	C0322	
S0362	C0341	
S0362	C0342	
S0362	C0343	
S0363	C0344	
S0363	C0345	
S0363	C0346	
S0363	C0347	
S0363	C0348	
S0363	C0349	
